# Integration of expert knowledge in the definition of Swiss pear core collection

**DOI:** 10.1038/s41598-019-44871-3

**Published:** 2019-06-20

**Authors:** J. Urrestarazu, C. Kägi, A. Bühlmann, J. Gassmann, L. G. Santesteban, J. E. Frey, M. Kellerhals, C. Miranda

**Affiliations:** 1 Department of Agronomy, Biotechnology and Food Science, Public University of Navarre, 31006 Pamplona, Spain; 2 Federal Office for Agriculture, 3003 Bern, Switzerland; 3 Agroscope, 8820 Wädenswil, Switzerland

**Keywords:** Genetics, Plant sciences

## Abstract

Core collections (CCs) constitute a key tool for the characterization and management of genetic resources (GR). When the institutions involved in GR preservation decide to define a CC, they frequently prefer to select accessions based not only on strictly objective criteria, but also to add others following expert knowledge considerations (popularity, prestige, role in breeding history, or presence of phenotypic features of interest). The aim of this study was to evaluate the implications of approaches that combine formal analytical procedures and expert knowledge on the efficiency of CC definition through a case study to establish a pear CC from the Swiss National Pear Inventory. The CC had to represent a maximum of the genetic diversity, not to exceed 150 accessions, and required to include a priority set (SPPS) with 86 genotypes selected based on expert knowledge. In total, nine strategies were evaluated, resulting of combining compositions of the dataset sampled, sampling sizes and methods. The CCs sampled by mixed approaches provided similar scores, irrespective of the approach considered, and obtained similar efficiency in optimizing the genetic diversity retained. Therefore, mixed approaches can be an appropriate choice for applications involving genetic conservation in tree germplasm collections.

## Introduction

One of the major challenges genebank managers face to is the necessity of increasing the accessibility of their collections to a broad panel of potential users such as plant breeders, geneticists and farmers^1^. However, the sheer size of many of these genebanks is often a barrier hampering their characterization and evaluation, limiting thus their further effective use^2–4^. As a consequence, the core collection (CC) concept (i.e., a limited set of accessions derived from an existing germplasm collection chosen to represent the genetic spectrum of the whole collection), was proposed long time ago as an approach to overcome this limitation^5^. Since a core collection is smaller in size compared to the whole collection, it enables characterization and management operations to be handled more efficiently and effectively^2,3^.

Before molecular identification became available, CC selections were established focusing on morphology, eco-geography and/or passport information^6–8^. Later on, the widespread introduction of molecular markers for characterization, switched CC selection to be based either exclusively on genetic diversity^9–11^, or in combination with morphological and agronomical data^4,12,13^. In that context, many theoretical and practical considerations on how to define CCs have been accumulated in the last three decades, dealing with three interconnected issues highlighted by Odong^3^: (i) CC can be defined differently depending on their purpose; (ii) there are different statistical methods to define them, and (iii) there is no consensus on how the quality of a CC should be measured. An additional aspect to take into account when defining a CC is the balance between representing total diversity and the usefulness of the core to the potential users^14^. However, institutions in charge of preserving traditional plant material often prefer favoring the inclusion of some varieties following criteria different from those that arise solely from statistical considerations, and that can be globally covered by the term ‘expert knowledge’. For instance, it is sensible that those institutions prefer including in CC cultivars which have played an important role in breeding history, are popular, prestigious or emblematic among local growers and/or consumers, are used as a standard in research in a given species, or exhibit some phenotypic features of interest. Some of these conditions arise on the unneglectable role that genetic resources play in an ecological and cultural dimension, beyond the strict maximization of diversity conservation^15,16^. Therefore, there is a need of evaluating how building a CC based on mixed approaches for entry selection, i.e. combining formal analytical procedures maximizing the diversity with some additional ‘pragmatic’ considerations, affects the efficiency of the core collection. To the best of our knowledge, despite its relevance, this issue has not been addressed to date.

In this context, the main aim of this study was to evaluate the implications that using mixed approaches that combine formal analytical procedures and ‘expert knowledge’ have on the efficiency of CC definition. This study is based in a real study case, where this question arose while intending to establish a CC out of 1198 pear (*Pyrus communis* L.) accessions from the Swiss National Pear Inventory coordinated by the Federal Office for Agriculture (FOAG). All accessions had been characterized using a common set of 16 SSR markers, but there was a clear interest of institutions and germplasm curators to consider the inclusion of some entries in the CC on the basis of ‘expert knowledge’ considerations. At the same time, this study also allowed evaluating the genetic diversity at the Swiss national-level for this species for the first time, as well as shedding light into the distribution of this material into population subdivisions.

## Results

### Characterization of the germplasm of the swiss national pear inventory

#### SSR polymorphism and genetic diversity

All the SSR markers considered in this study were polymorphic. Due to complex scoring and unreliable microsatellite profiles using marker CH05c06, we decided to exclude it from the study. A pairwise comparison of multilocus SSR profiles among the 1,198 accessions allowed identifying 186 groups of accessions sharing identical SSR profiles (Supplementary Table S1), leading to the identification of 457 SSR duplicated accessions (35% of redundancy). A total of 412 alleles were identified in the 841 unique genotypes across the 15 SSR markers (average number of alleles per locus = 27.4; *N*_*E*_ = 7.73), 78.6% and 50.0% occurring at frequencies below 5% and 1%, respectively. Further information about the genetic diversity of the Swiss National Pear Inventory is provided in Supplementary Text S1.

#### Genetic structure

**Figure 1:**
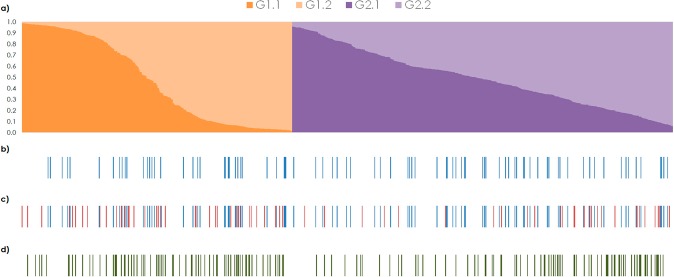
Graphical display of the results of the STRUCTURE analysis. Each genotype is represented by a vertical bar partitioned into *K* = 4 segments representing the inferred membership fraction in the four subgroups inferred (**a**). Eighty-six genotypes included in SPPS are highlighted by colored blue bars (**b**). One hundred and fifty genotypes sampled in A-OMin_All_ are highlighted by bars: the genotypes included in SPPS are indicated in blue, and the genotypes sampled by the analytical method are indicated in red (**c**). One hundred and fifty genotypes sampled in A-FNK_All_ are highlighted by green bars (**d**).

The analysis of the 841 unique genotypes using STRUCTURE revealed that the rate of change Δ*K* over the range of *K* values showed a clear maximum for *K* = 2 (Δ*K* = 604.8; Supplementary Fig. S1a). This clustering reflected an asymmetric division of the germplasm in two main groups, one with 349 genotypes (G1) and a second with 492 (G2). A secondary peak at *K* = 4 (Δ*K* = 82.1) was identified, suggesting that the diversity could be sub-structured. Thus, a second-level (nested) application of the STRUCTURE software was applied separately on each of the two main groups defined in the first analysis. For the first group (G1), the results indicated a subdivision at *K*_G1_ = 2 (Δ*K*_G1_ = 443.4) (Supplementary Fig. S1b), whereas the second (G2) was partitioned at *K*_G2_ = 2 (Δ*K*_G2-2_ = 100.4) and *K*_G2_ = 3 (Δ*K*_G2-3_ = 63.2) (Supplementary Fig. S1c). To analyze the robustness of the groups indicated above, simulations were examined focusing on the mean assignation probability (*qI*) and the proportion of genotypes strongly assigned (*qI* ≥ 0.80) to each partitioning level^17–19^. Results about the genetic structure for Swiss National Pear Inventory are detailed in Supplementary Text S1, including the definition of the partitioning levels from STRUCTURE, the level of intra-group variability as well as the degree of differentiation between the groups inferred. In summary, four subgroups were adopted as the most suitable level of subdivision for the Swiss National Pear Inventory (Fig. 1a). The minimum spanning networks (MSN) based on Bruvo’s distance (Supplementary Fig. S2) were consistent with the results obtained with the Bayesian clustering method supporting the existence of the above mentioned genetic groups. As described in Supplementary Text S1, some interesting associations between the clustering of the genotypes in subgroups and the particular usage/aptitude of the cultivars were revealed.

### Comparison of the different strategies used to define CCs

#### Primary characteristics of the swiss pear priority set (SPPS) in terms of genetic diversity

The core collection was required to include 86 ‘Priority’ genotypes, selected by the collection stakeholders on the basis of their ‘expert knowledge’, mainly based on their historical relevance or exceptional pomological features. These 86 ‘Priority’ genotypes were hereafter referred as the Swiss Pear Priority Set (SPPS). Genetic characteristics of the SPPS, namely those selected according to ‘expert knowledge’ considerations, were compared with subsets of the same size sampled by the ASLS (A-86) or the M-method (M-86). A comparison was performed at three levels, including distance-based criteria, allelic diversity parameters, and the distribution of the sampled genotypes on the inferred genetic structure of the whole population.

The formal analytical methods, as expected, optimized some genetic distances better than the ‘expert knowledge’ approach. For *E-E*, irrespective of the genetic distance used (*D*_*B*_ or *D*_*CE*_), A-86 and M-86 maximized distance 2.5–7.0% more than SPPS, and these two subsets were much more efficient in optimizing *E-NE*. A-86 particularly outperformed the other strategies, as *E-NE* was 18.9% higher than in the SPPS. However, the SPPS was 8.7–9.4% more efficient in optimizing *A-NE* distance. These results indicate that SPPS contains a higher level of redundancy. The differential pattern of genetic relatedness between subsets was noticeable on heat maps (Supplementary Fig. S3), as the plot displaying all pairwise comparisons between genotypes included in the SPPS showed greater color heterogeneity than those obtained for A-86 and M-86, evidencing that some of the SPPS genotypes were moderately similar to each other.

For the parameters directly accounting for the retained allelic variation (*C*_*V*_, *N*_*E*_ and *H*_*E*_) remarkable differences were also found between subsets (Table 1). SPPS retained two third of the alleles present in the whole population, whereas both A-86 and M-86 captured ca. 90%. The lower efficiency of SPPS in retaining the allelic diversity was also reflected in its lower number of effective alleles (N_E_) (Table 1). *N*_*E*_, by definition, positively correlates with *H*_*E*_^20^; accordingly, *H*_*E*_ for A-86 and M-86 was slightly higher than that found in SPPS (Table 1).

**Table 1:** Genetic parameters of core subsets selected by different methods at 86 genotypes sample.

Strategy ID	*Bruvo genetic distance*			*D*_*CE*_	*S*_*H*_	*H*_*E*_	*N*_*E*_	*C*_*V*_ *(%)*
	*E-E*	*E-NE*	*A-NE*	*E-E*				
Swiss National Pear Inventory	0.591	—	—	0.834	4.967	0.830	7.73	100.00
Priority subset (SPPS)^a^	0.603	0.403	0.385	0.842	4.928	0.832	7.74	66.67
A-86^b^	0.648	0.497	0.425	0.878	5.222	0.873	10.28	89.25
M-86^c^	0.630	0.464	0.422	0.864	5.158	0.857	9.21	91.00

Footnotes:

*E-E*: Average entry to entry distance, *E-NE*: average distance between each entry and the nearest entry, *A-NE*: Average distance between each genotype of the collection and the nearest entry, *D*_*CE*_: average genetic distance of Cavalli-Sforza and Edwards, *S*_*H*_: Shannon-Weaver diversity index, *H*_*E*_: Nei diversity index, *C*_*V*_: allelic coverage in percentage.

^a^Priority subset selected by “expert knowledge” considerations (SPPS, i.e. Swiss Pear Priority Set).

^b^Each parameter was optimized by performing 80 independent runs with equal weight given to each of the parameters (CV, average and minimum DCE, SH, and HE).

^c^Each parameter was optimized by performing 200 independent runs.

Despite the fact that SPPS was selected with no consideration for genetic data, this subset sampled a rather balanced proportion of the four genetic groups inferred in the structure (Fig. 1a,b), although one genetic group (G1.1) was slightly underrepresented (7% of the group size vs. 11–12% sampled in the remaining groups). However, the formal analytical methods sampled the genetic groups inferred in a rather unbalanced way, overrepresenting G1.2 (14–16% of the genotypes assigned) and underrepresenting G2.1 (3–4%). This was mainly a consequence of the allelic variability contained within the genetic groups inferred (Supplementary Text S1), the highest for G1.2 and the lowest in G2.1, especially for the number of exclusive alleles, which decreased the chance to sample genotypes from G2.1 when using analytical methods.

#### Comparison of CCs sampled using purely analytical procedures

**Figure 2:**
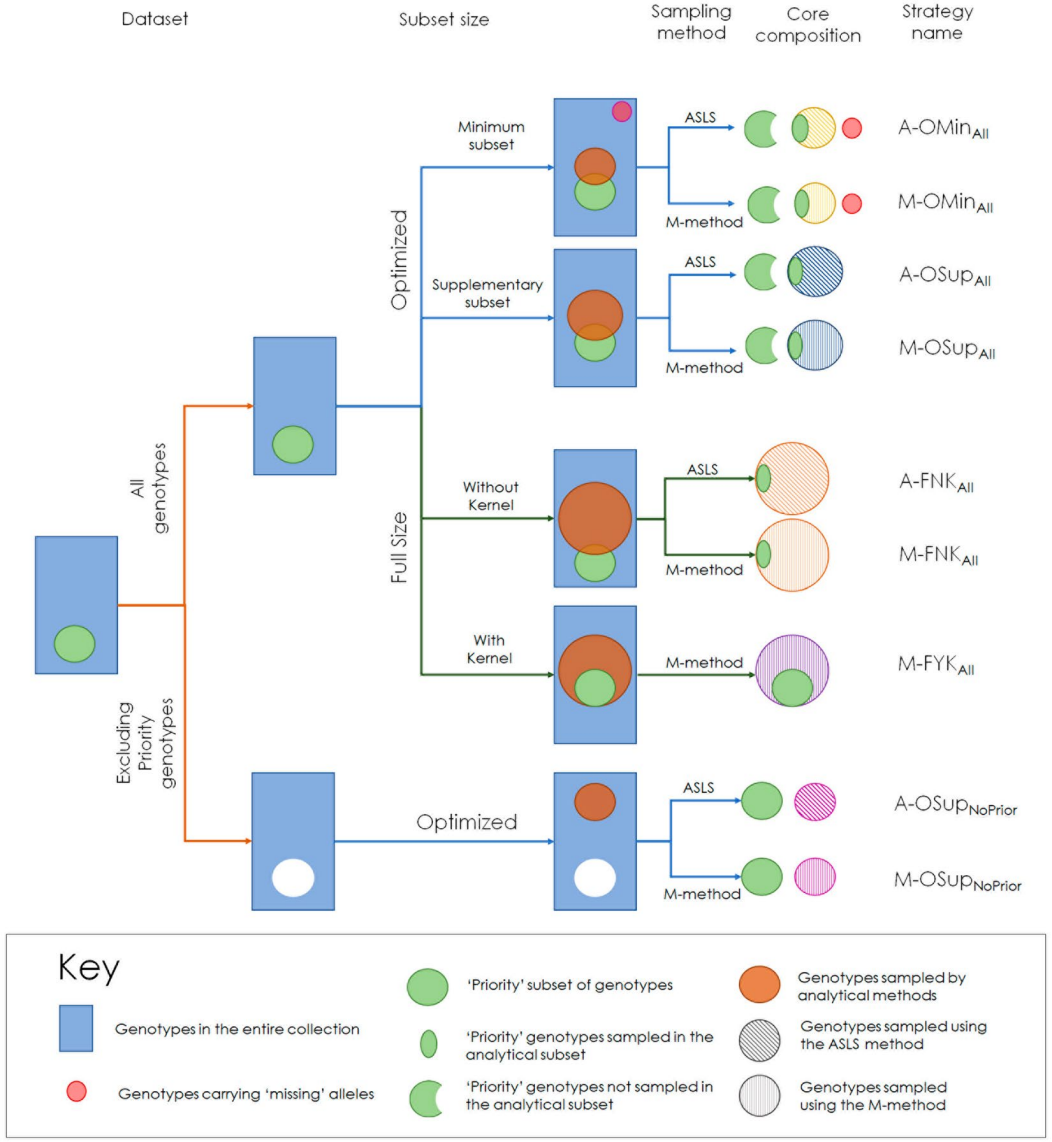
Graphical summary of the nine sampling strategies used after combining different compositions of the dataset sampled, subset sampling sizes and sampling methods.

**Figure 3:**
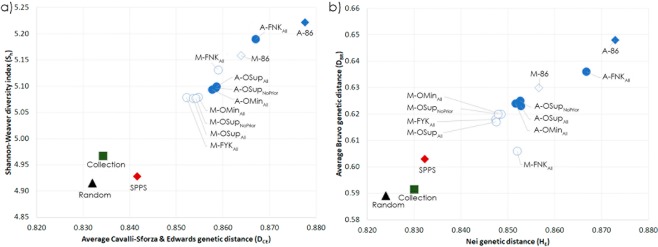
Comparison of the different core collections obtained through all strategies tested in the study according to (**a**) Shannon-Weaver diversity index (*S*_*H*_) and average Cavalli-Sforza and Edwards genetic distance (*D*_*CE*_) and (**b**) average Bruvo genetic distance (*D*_*B*_) and Nei diversity index (*H*_*E*_).

Nine CCs, generated by combining different compositions of the dataset sampled (complete dataset or sampling only within the genotypes not included in SPPS), subset sizes (optimized subsets or full-size subsets) and sampling methods (M-method or ASLS method), were evaluated within this study. The nine corresponding sampling strategies used and their acronyms are described in Fig. 2 and Table 2. The CC sampled by the ASLS method (A-FNK_All_, Table 3, Fig. 3) clearly outperformed that sampled by the M-strategy (M-FNK_All_) in optimizing *E-E* and *E-NE* distances and the number of effective alleles per locus (*N*_*E*_), whereas the M-FNK_All_ method was more efficient in optimizing *A-NE* distance. The effectiveness was increased in a range from 2.6% for *A-NE* to 9.2% for *E-NE*. For *D*_*CE*_, the rest of the allele diversity parameters (*S*_*H*_, *H*_*E*_) and the allele coverage (*C*_*V*_), the efficiency gains of the ASLS method were more modest, with scores 0.9% to 1.7% higher than those obtained for the M-strategy. The genotypes sampled in A-FNK_All_ and A-OMin_All_ are represented using a minimum spanning network in Supplementary Fig. S4.

**Table 2:** Acronyms and characteristics of the sampling strategies evaluated in this study to define core collections.

Strategy ID	Sampling method	Sampling size	Dataset sampled
A-OMin_All_	**A**SLS	**O**ptimized, **Min**imal subset	**All** genotypes
M-OMin_All_	**M**-Strategy	**O**ptimized, **Min**imal subset	**All** genotypes
A-OSup_All_	**A**SLS	**O**ptimized, **Sup**plementary subset	**All** genotypes
M-OSup_All_	**M**-Strategy	**O**ptimized, **Sup**plementary subset	**All** genotypes
A-FNK_All_	**A**SLS	**F**ull size, **N**o **k**ernel defined	**All** genotypes
M-FNK_All_	**M**-Strategy	**F**ull size, **N**o **k**ernel defined	**All** genotypes
M-FYK_All_	**M**-Strategy	**F**ull size, **k**ernel defined	**All** genotypes
A-OSup_NoPrior_	**A**SLS	**O**ptimized, **Sup**plementary subset	‘**No**n-**Prior**ity’ genotypes
M-OSup_NoPrior_	**M**-Strategy	**O**ptimized, **Sup**plementary subset	‘**No**n-**Prior**ity’ genotypes

**Table 3:** Genetic parameters of core subsets selected by purely analytical procedures and by mixed procedures (analytical + ‘expert knowledge’).

Strategy ID	*Bruvo genetic distance*			*D*_*CE*_	*S*_*H*_	*H*_*E*_	*N*_*E*_	*C*_*V*_ *(%)*
	*E-E*	*E-NE*	*A-NE*	*E-E*				
**Swiss National Pear Inventory**	0.591	—	—	0.834	4.967	0.830	7.73	100.00
**Random core**	0.589	0.354	0.362	0.932	4.915	0.824	7.46	74.30
**Purely analytical procedures**								
A-FNK_All_	0.636	0.463	0.394	0.867	5.190	0.867	9.80	97.00
M-FNK_All_	0.606	0.424	0.384	0.859	5.131	0.852	9.05	98.00
**Mixed procedures (analytical + ‘expert knowledge’)**								
A-OMin_All_	0.624	0.388	0.367	0.858	5.093	0.852	8.85	90.00
A-OSup_All_	0.623	0.396	0.367	0.859	5.100	0.853	9.00	88.50
A-OSup_NoPrior_	0.625	0.385	0.368	0.858	5.097	0.853	8.91	87.50
M-OMin_All_	0.620	0.390	0.367	0.855	5.079	0.849	8.68	89.50
M-OSup_All_	0.617	0.385	0.366	0.854	5.077	0.847	8.69	89.00
M-OSup_NoPrior_	0.620	0.384	0.366	0.854	5.077	0.848	8.68	89.00
M-FYK_All_	0.618	0.392	0.364	0.852	5.079	0.847	8.74	90.00

Footnotes:

*E-E*: Average entry to entry distance, *E-NE*: average distance between each entry and the nearest entry, *A-NE*: Average distance between each genotype of the collection and the nearest entry, *D*_*CE*_: average genetic distance of Cavalli-Sforza and Edwards, *S*_*H*_: Shannon-Weaver diversity index, *H*_*E*_: Nei diversity index, *C*_*V*_: allelic coverage in percentage.

#### Comparison of CCs sampled with mixed approaches

The CCs sampled by mixed approaches (Table 3; Fig. 3) provided similar scores, differing 1–3%, for Bruvo distance criteria. Irrespective of the mixed approach considered, the maximization of *D*_*CE*_, *S*_*H*_, *H*_*E*_ and *N*_*E*_ was essentially identical, and all strategies retained a high number of alleles (*C*_*V*_ > 87.5%).

When the CCs defined using mixed strategies were compared to A-FNK_All_, the latter clearly outperformed them at optimizing *E-NE* (19.2% more efficiently), though mixed strategies were 6.8–7.6% more efficient in optimizing *A-NE* distance. For the remaining criteria, the efficiency of A-FNK_All_ was similar to the mixed strategies for *D*_*CE*_, *S*_*H*_ and *H*_*E*_ (<2%), and higher for *N*_*E*_ (11.4%). A similar pattern was observed for M-FNK_All_, but overall efficiency was closer to that of mixed strategies. The highest differences in efficiency were observed for *E-NE*, *A-NE* and *N*_*E*_ (3–9% more efficient), whereas for the other criteria, efficiency was essentially identical (<1%). The mixed strategies retained ca. 90% of the alleles present in Swiss National Pear Inventory, while a nearly total recovery (*C*_*V*_ = 97–98%) was obtained when using the formal analytical strategies.

The distribution of the sampled genotypes in the genetic structure of the collection was assessed for the best performing analytical strategy (A-FNK_All_) and for one of the mixed strategies (A-OMin_All_). A-FNK_All_ showed a rather unbalanced distribution over the structure (Fig. 1d), skewed in favor of G1.2 and G2.2, both in absolute number and proportion of genotypes sampled from each subgroup. In fact, 26% and 23% of the genotypes assigned to G1.2 and G2.2, respectively, were included in A-FNK_All_, while only 8% and 15% were sampled from G2.1 and G1.1, respectively. A similar bias appeared for the genotypes sampled using the A-OMin_All_ strategy but the SPPS subset partially compensated this bias when included in the CC (Fig. 1c), resulting in a more balanced representation of the four subgroups in the CC. Thus, it can be considered that mixed strategies provided a more balanced and exhaustive representation of the genetic structure of the collection than the CCs developed exclusively by formal analytical strategies.

## Discussion

The high level of diversity in pear germplasm found at the Swiss national-level agreed with results obtained in other European countries, such as Spain^17^, Italy^21^ and Sweden^22^. Despite the fact that the allelic variation found at the Swiss National Pear Inventory was very large, the underlying population structure is weak. Using a Bayesian clustering method allowed identifying two genetic groups reflecting major divisions of the germplasm, which after applying a nested-approach of the same method were further subdivided in two subgroups each, revealing moderate, but significant, differentiation among them. Remarkable differences between the inferred groups and subgroups were revealed at the allelic level, pointing out a further evidence of a division of the germplasm in different partitioning levels.

The large diversity found in pear, that occurs in species with a complex history like apple^18^, is consistent with the weak bottleneck reported in connection with the domestication of perennial fruit tree species^23,24^. The mode of reproduction, altogether with human-mediated activities, have played a key role in the genetic variation and population structure that can be found nowadays in most of the fruit tree species^23^. Vegetative propagation methods adopted since ancient times favored the dispersal of germplasms across different regions, thus contributing to the diversification of the existing genepool in multiple regions through unintentional crosses or human-mediated activities (selection and breeding). In addition to this spatially and temporally dynamic process, the self-incompatibility system presumably has been a decisive factor in encouraging high levels of diversity in *Pyrus* spp.

Core collections in this study were sampled with the objective of obtaining generalist CCs. For that reason, all accessions in the entire collection should be maximally represented, agreeing with what Odong *et al*.^3^ defined as a CC-I collection. Ideally, each accession in the whole collection should be represented in the core by an entry that is most similar to itself. CCs have been validated following the indications by Odong *et al*.^3^ that is, using preferably distance-based indices and criteria not used in the selection phase, supplemented by other classic indices suited to the evaluation of generalist collections such as Shannon Index (*S*_*H*_) and allele coverage (*C*_*V*_).

In the CCs sampled by purely analytical methods, the classic indices provided virtually identical results, something expected, as both *S*_*H*_ and *C*_*V*_ were included in the sampling strategies. However, core subsets generated using the formal analytical strategies considered, highlighted a trade-off in the effectiveness of optimizing genetic distances, so that the decision must be determined in terms of fitness-for-use. If our CCs had been selected with the aim to represent the extreme values in the collection, optimizing *E-NE* should have been the objective^3^ and, therefore, A-FNK_All_ would be the best-suited strategy. However, as the CCs were designed to represent the accessions in the collection, *A-NE* is the distance to optimize, and M-FNK_All_ the most suited analytical strategy.

The differences between strategies in the CCs sampled by mixed methods were nearly negligible (<3% for all the criteria considered), revealing a certain “buffer” effect of the SPPS subset. Such effect could be expected, as the SPPS subset accounts for ca. 57% of the final CCs, and the genotypes in it were rarely included in the optimized subsets sampled by analytical methods. However, an additional optimized subset with low sampling intensity (7–8% of the genotypes in the whole collection) was sufficient to efficiently offset the shortcomings of the SPPS subset and represent the genetic diversity found in the whole collection. Small core subsets have previously showed high efficiency optimizing the retained diversity^10,25–27^. Moreover, the development of core collections in other species^12,28–30^ has evidenced that core sets maximized for diversity using a set of specific attributes (molecular or phenotypic), at the same time, maximize unknown diversity. The CCs selected by mixed strategies have optimized diversity parameters as efficiently as the least efficient of the purely analytical strategies evaluated as benchmarks.

The greatest differences between purely analytical and mixed approaches appeared in the distance criteria. As *A-NE* is the distance to optimize in generalist CCs, mixed strategies clearly outperformed the purely analytical ones. At the same time, a trade-off between *A-NE* and allele recovery was observed. The Swiss National Pear Inventory shows a particularly high number of rare alleles, with ca. 17% of the alleles being present in just one genotype. Thus, increasing *C*_*V*_ from 87–90% of mixed strategies to 97–98% or purely analytical strategies resulted in an increased redundancy (and *A-NE* distance) due to the higher number of genotypes just providing those unique alleles. Selection of a core collection that fulfills all genetic criteria is impracticable because of the inter-relationships of the evaluated parameters^2^. Considering all analyzed criteria and the purpose of the CC, the best results for the Swiss National Pear Inventory collection were obtained using the mixed strategies. In collections with lower presence of rare/unique alleles, the balance between *A-NE* and *C*_*V*_ probably would have been smaller. In any case, it seems reasonable to consider that CC sampling strategies such as those tested in this study (combining expert knowledge and optimized subsets using SSR data) are capable of generating CCs that are similarly efficient to those obtained by purely analytical methods.

## Conclusion

The balance between representing diversity and the usefulness of the CC to the intended use or user is highly relevant when defining a core collection. Core sets maximized for diversity using molecular attributes can, at the same time, maximize unknown diversity. However, institutions in charge of preserving traditional plant material often prefer favoring the inclusion of some cultivars due to other reasons, i.e., historic value or exhibiting some phenotypic features of interest. We have presented a case study using the Swiss National Pear Inventory, testing the efficiency of CCs sampled using a mixed approach in which part of the genotypes were selected by ‘expert knowledge’ and then supplemented with a highly optimized subset using SSR data. The final CCs selected by this approach, obtained similar (and sometimes higher) efficiency in optimizing the genetic diversity retained within the CC when compared to CCs sampled by purely analytical methods. The results obtained in this study show that mixed approaches could be appropriate choices for applications involving genetic conservation in fruit tree germplasm collections.

## Materials and Methods

### Plant material

**Figure 4:**
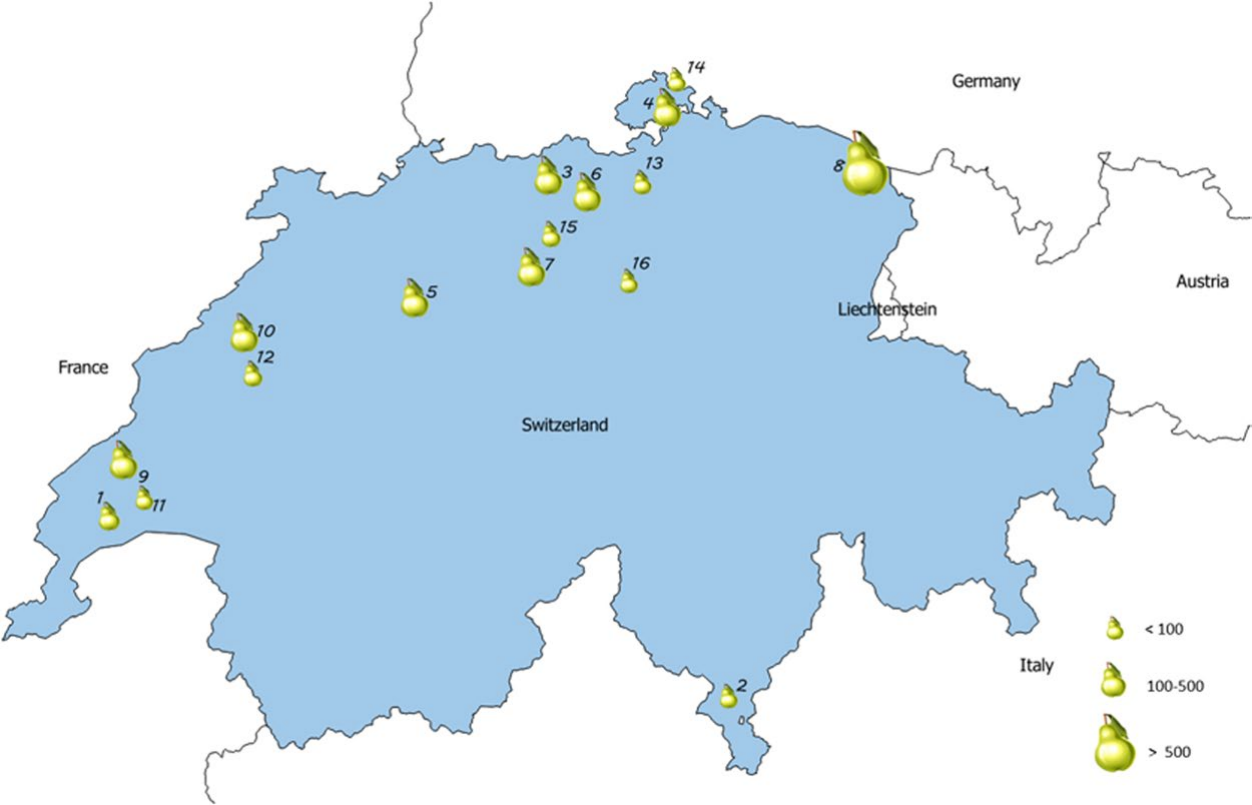
Geographic location of the Swiss pear germplasm collections included in this study: 1, Arboretum national du vallon de l′Aubonne; 2, Collezione d’introduzione Manno; 3, Duplikatsammlung Bözberg-Vierlinden; 4, Dupliatsammlung Griesbach SH; 5, Einführungssammlung Birnen Inforama Oeschberg; 6, Einführungssammlung ProSpecieRara Baden-Münzlishausen; 7, Einführungssammlung ProSpecieRara Büron; 8, Einführungssammlung Riedern Roggwil; 9, Parcelle basse tige d’Aclens (VD); 10, Parcelle basse tige de Pierre-à-Bot (NE); 11, Parcelle primaire haute tige d’Aclens (VD); 12, Parcelle primaire haute tige de Pierre-à-Bot (NE); 13, Primärsammlung Höri; 14, Primärsammlung Obst “Hofen”; 15, Primärsammlung ProSpecieRara Dürrenäsch; 16, Primärsammlung ProSpecieRara Knonau.

A total of 1198 pear accessions of the Swiss National Pear Inventory was used in this study (Supplementary Table S1). This germplasm was collected by several NGOs from all over Switzerland since around 1970 and is nowadays conserved in duplicates in almost 30 collections, 16 of which provided material for DNA analyses (Fig. 4). All accessions were characterized using a set of 16 SSR markers^31^ (Supplementary Text S2).

### Genetic diversity analysis

The multilocus SSR profiles of all accessions were compared. The number of alleles per locus (*A*), the number of rare alleles per locus (*B*, number of alleles with a frequency below 5% and 1%), the number of the effective alleles (*N*_*E*_), and the observed (*H*_*O*_) and expected heterozygosity (*H*_*E*_) were calculated using the SPAGeDi version 1.3 software package^32^.

STRUCTURE version 2.3.4^33^ was used to estimate the number of hypothetical groups (*K*) and to quantify the membership probability of each genotype to the identified groups. The clustering was performed under the admixture model, and correlated allelic frequencies for *K* values ranging from one to 10, with five independent runs each, with a burn-in phase of 2 × 10^5^ iterations, and a sampling phase of 5 × 10^5^ replicates. The analysis was run using the recessive allele approach^34^, encoding the genotypes following the recommendations given for polyploid species in the software manual and as used in previous studies^17–19,35–37^. Structure Harvester ver. 0.6.93^38^ was applied to estimate the most suitable *K* value according to the Δ*K* method defined by Evanno *et al*.^39^. Genotypes were assigned to the groups for which they had the highest assignation probability (*qI*), considering a strong membership coefficient of an accession to a particular group if *qI* ≥ 0.80^17,18,37,40^. When the results suggested sub-structuring of the diversity above the selected *K* value, a second level (nested) STRUCTURE analysis was performed for each group separately^17,18,37,41,42^. The results were graphically displayed using DISTRUCT 1.1^43^. Genetic differentiation between groups defined by STRUCTURE was examined through an analysis of molecular variance (AMOVA) using Genodive v2.0b23^44^.

### Definition of CCs

The Swiss Pear CC should maximize the representativeness of the genetic diversity contained in the Swiss National Pear Inventory, without exceeding the size of 150 accessions, due to management and budgetary reasons. Additionally, the collection was required to include the 86 genotypes of the SPPS, mainly based on their historical relevance or exceptional pomological features. Therefore, the final collection would be selected using a mixed approach, combining the SPPS and a representative subset of genotypes, selected from the SSR data, while keeping the final core within the required size limits.

#### Tested strategies

In total, nine sampling strategies (Fig. 2) were evaluated, that resulted of combining different compositions of the dataset sampled, subset sizes and sampling methods:

- **Dataset composition**. Two strategies differing in the accessions included in the dataset were used:
  i. Complete database: All the genotypes in the Swiss National Pear Inventory were included in the sampling database.
  ii. Non-priority database: The genotypes corresponding to SPPS were removed from the database prior to the sampling procedure.
- **Sampling sizes**. Sampling strategies were performed at two size criteria:
  i. Optimized sizes. Since the accessions of the SPPS set were chosen according to criteria different from the marker profile, it was expected that the subset selected from SSR data would include only part of the SPPS genotypes. Therefore, first we assessed the subset size needed not to exceed the required CC size, when the subset is supplemented by the missing SPPS accessions (Supplementary Fig. S5). Then, two sampling size strategies were performed:
    *Minimal subset*: The core subset was sampled for 60 individuals (≈7% of the collection), as that size was near the lowest threshold (5%) in the range of CC size considered optimal in fruit tree core collection^2^. In this strategy, as the sum of the subset and priority accessions did not reach the required size, additional genotypes were selected manually until reaching it. The criterion used was to include those genotypes carrying missing alleles in descending order of Bruvo’s genetic distance^45^ to the closest accession already included in the core.
    *Supplementary subset*: The core subset was sampled for the highest size that, supplemented with the missing SPPS genotypes, resulted in a core with 150 individuals.
  ii. Full size subsets. In this strategy, core subsets with 150 genotypes were sampled:
    *With kernel*: If the software allowed it, it was specified that the SPPS genotypes were to be compulsorily included in the subset, forming what is known as a kernel^46^. When a kernel is defined, the sampling procedure focuses on maximizing diversity for alleles not included in the kernel.
    *Without kernel*: Subsets that were selected using exclusively marker data, as a benchmark to compare the performance of formal analytical procedures to ‘mixed’ strategies involving SPPS accessions selected by criteria other than their microsatellite profiles (‘expert knowledge’).
- **Sampling methods**. Two different sampling methods were used:
  i. The maximizing method (M-method) implemented in Mstrat^46^, which examines all possible core subsets and select those that maximize the number of alleles for one sample size. The program allows to specify accessions that will always be included in the core subset (kernel), in this case maximization focuses on complementing alleles not included in the kernel accessions. The Shannon-Weaver (*S*_*H*_) diversity index was used as a second criterion to classify core subsets. One hundred replicates and 200 iterations of Mstrat were generated independently when this sampling strategy was used.
  ii. The advanced stochastic local search method (ASLS method) implemented in Core Hunter II^20,47^. The software is able to select core subsets using diverse allocation strategies by optimizing many parameters simultaneously, whereby the best solution among all replicas is reported. The allocation strategy used involved optimizing the following five measures simultaneously with equal weight assigned to each one: average and minimum Cavalli-Sforza and Edwards genetic distance (*D*_*CE*_) between core entries, allelic coverage or number of alleles (*C*_*V*_), Shannon-Weaver diversity index (*S*_*H*_), and Nei diversity index (*H*_*E*_). For this method, 80 independent runs were performed.

The acronyms of the nine sampling strategies tested in this study are specified in Fig. 2 and Table 2. Additionally, a random core subset with 150 genotypes was selected using the ‘sample’ function in R, where samples were selected arbitrarily without replacement of genotypes.

### Evaluation of the diversity retained through the different strategies

The evaluation and comparison between CCs obtained from the different approaches were conducted focusing on distance-based criteria, parameters associated to allelic variability and the graphical distribution of the genotypes selected on the genetic structure inferred for the whole collection.

Three genetic distance-based criteria were considered when evaluating the quality of the defined CCs through formal analytical procedures *versus* mixed approaches: (i) the average genetic distance between all the entries of each CC (*E-E*), (ii) the average distance between each entry and the nearest neighboring entry for each CC (*E-NE*), and (iii) the average distance between each genotype of the entire collection and the nearest entry in each CC (*A-NE*). *E-E* was assessed for the Bruvo (*D*_*B*_) and Cavalli-Sforza and Edwards (*D*_*CE*_) genetic distances^45,48^, whereas the two latter were assessed only for *D*_*B*_. For the *E-E* and *E-NE*, the larger the value the higher the quality of the CC, the opposite is true for *A-NE*^3^. Additionally, the parameters used to optimize core subsets by the ASLS method (*D*_*CE*_, *C*_*V*_, *S*_*H*_ and *H*_*E*_) were also used to evaluate the quality of the CCs. Lastly, the pairwise Bruvo genetic distances among the genotypes sampled in each strategy were represented using heat maps to graphically display relatedness.

The distribution of the genotypes of the CCs on the underlying genetic structure of the whole Swiss National Pear Inventory was evaluated through two approaches. First, making use of the clustering obtained through the Bayesian model-based method, the distribution and representativeness of the genotypes included in each CC on the inferred genetic groups were examined. Second, Minimum Spanning Network (MSN) plots were drawn based on the Bruvo’s distance^45^ using the ‘poppr’ R-package^49^ and compared for different strategies.

## Supplementary information

Table S1

Supplementary Figures and Texts
