# Supplementary material for: Integration of expert knowledge in the definition of Swiss pear core collection
Source: Sci Rep. 2019 Jun 20;9:8934. doi: 10.1038/s41598-019-44871-3 (PMC6586639; doi:10.1038/s41598-019-44871-3)
Supplement: Supplementary file 1 — Table S1 [file 41598_2019_44871_MOESM1_ESM.pdf]

SUPPLEMENTARY INFORMATION

Integration of expert knowledge in the definition of Swiss pear core collection

Urrestarazu J[1]*, Kägi C[2]*, Bühlmann A[3], Gassmann J[3], Santesteban LG[1], Frey JE[3], Kellerhals M[3], and Miranda C[1]

*These authors contributed equally to this work

Corresponding author: Jorge Urrestarazu (jorge.urrestarazu@unavarra.es)
Department of Agronomy, Biotechnology and Food Science, Public University of Navarre, 31006 Pamplona, Spain. Phone number: +34 948169127

[1] Department of Agronomy, Biotechnology and Food Science, Public University of Navarre, 31006 Pamplona, Spain.

[2] Federal Office for Agriculture, 3003 Bern, Switzerland.

[3] Agroscope, 8820 Wädenswil, Switzerland.

Supplementary Table S1. List of 1198 accessions analyzed in this study classified according to their duplicated group (SSR profile). The probability of assignment for each genotype to the genetic groups inferred with STRUCTURE is provided.

Accession name	Accession code	Location	Gene group	Group	*qI* max	Subgroup	*qI* max nested	*qI* G1	*qI* G2	*qI* G1.1	*qI* G1.2	*qI* G2.1	*qI* G2.2
Zuckerbirne	14-003-973	Büron	1	**G1**	**0.972**	**G1.2**	**0.972**	**0.972**	0.028	0.037	**0.963**	--	--
Zuckerbirli	14-003-950	Büron	1	**G1**	**0.972**	**G1.2**	**0.972**	**0.972**	0.028	0.037	**0.963**	--	--
Poire d'Adge	14-003-2514	Aubonne	2	**G2**	0.693	**G2.1**	0.693	0.307	0.693	--	--	0.517	0.483
Poire Beurré d'Adze	14-003-49	Pierre-à-bot	2	**G2**	0.693	**G2.1**	0.693	0.307	0.693	--	--	0.517	0.483
Brühlmanns Butterbirne	14-003-85	Pierre-à-bot	3	**G2**	**0.932**	**G2.1**	**0.932**	0.068	**0.932**	--	--	0.692	0.308
unknown	14-003-1471	Büron	3	**G2**	**0.932**	**G2.1**	**0.932**	0.068	**0.932**	--	--	0.692	0.308
Poire Chretien	14-003-121	Pierre-à-bot	4	**G2**	**0.975**	**G2.1**	**0.975**	0.025	**0.975**	--	--	0.682	0.318
Poire Chrétien	14-003-122	Pierre-à-bot	4	**G2**	**0.975**	**G2.1**	**0.975**	0.025	**0.975**	--	--	0.682	0.318
Poire Froment	14-003-222	Pierre-à-bot	5	**G2**	0.738	**G2.1**	**0.892**	0.262	0.738	--	--	**0.892**	0.108
Poire Golliatson	14-003-1127	Pierre-à-bot	5	**G2**	0.738	**G2.1**	**0.892**	0.262	0.738	--	--	**0.892**	0.108
Poire Volléja	14-003-650	Pierre-à-bot	6	**G1**	0.579	**G1.2**	**0.935**	0.579	0.421	0.065	**0.935**	--	--
Poire Vouèza	14-003-2540	Pierre-à-bot	6	**G1**	0.579	**G1.2**	**0.935**	0.579	0.421	0.065	**0.935**	--	--
Bratbirne	14-003-806	Büron	7	**G2**	**0.947**	**G2.1**	**0.947**	0.053	**0.947**	--	--	0.721	0.279
Bratbirne	14-003-812	Büron	7	**G2**	**0.947**	**G2.1**	**0.947**	0.053	**0.947**	--	--	0.721	0.279
Spitzlibirne	14-003-930	Büron	8	**G2**	**0.880**	**G2.2**	**0.880**	0.120	**0.880**	--	--	0.211	0.789
Egnacher Spitzbirne	14-003-180	Bözberg-Vierlinden	8	**G2**	**0.880**	**G2.2**	**0.880**	0.120	**0.880**	--	--	0.211	0.789
unknown	14-003-1479	Büron	9	**G2**	**0.963**	**G2.2**	**0.963**	0.037	**0.963**	--	--	0.181	**0.819**
Herrenbirne	14-003-991	Büron	9	**G2**	**0.963**	**G2.2**	**0.963**	0.037	**0.963**	--	--	0.181	**0.819**
Petite Roussette	14-003-1548	Aclens	10	**G2**	0.618	**G2.2**	0.654	0.382	0.618	--	--	0.346	0.654
Poire rouge	14-003-1503	Aclens	10	**G2**	0.618	**G2.2**	0.654	0.382	0.618	--	--	0.346	0.654
Poire Rouselette	14-003-1549	Aclens	10	**G2**	0.618	**G2.2**	0.654	0.382	0.618	--	--	0.346	0.654
Maseron	14-003-868	Büron	11	**G2**	**0.937**	**G2.1**	**0.937**	0.063	**0.937**	--	--	**0.929**	0.071
Rebenbirne	14-003-869	Büron	11	**G2**	**0.937**	**G2.1**	**0.937**	0.063	**0.937**	--	--	**0.929**	0.071
Vincy 5	14-003-2518	Aubonne	12	**G1**	**0.911**	**G1.2**	**0.911**	**0.911**	0.089	0.474	0.526	--	--
unknown	14-003-1501	Büron	12	**G1**	**0.911**	**G1.2**	**0.911**	**0.911**	0.089	0.474	0.526	--	--
Wintertrübler	14-003-900	Büron	13	**G1**	**0.967**	**G1.1**	**0.967**	**0.967**	0.033	**0.873**	0.127	--	--
Poire à rissoles Marlioz	14-003-471	Aubonne	14	**G2**	0.778	**G2.1**	0.778	0.222	0.778	--	--	0.605	0.395
Poire Rissole	14-003-1570	Aclens	14	**G2**	0.778	**G2.1**	0.778	0.222	0.778	--	--	0.605	0.395
Poire à Rissole I	14-003-1155	Aclens	14	**G2**	0.778	**G2.1**	0.778	0.222	0.778	--	--	0.605	0.395
Poire à Rissole II	14-003-1156	Aclens	14	**G2**	0.778	**G2.1**	0.778	0.222	0.778	--	--	0.605	0.395
Poire à Rissole	14-003-506	Pierre-à-bot	14	**G2**	0.778	**G2.1**	0.778	0.222	0.778	--	--	0.605	0.395
Luzeiner Längler	14-003-376	Roggwil Hofen	15	**G2**	**0.968**	**G2.1**	**0.968**	0.032	**0.968**	--	--	**0.955**	0.045
Luzeiner Längler	14-003-376	Aubonne	15	**G2**	**0.968**	**G2.1**	**0.968**	0.032	**0.968**	--	--	**0.955**	0.045
Luzeiner Längler	14-003-376	Aclens	15	**G2**	**0.968**	**G2.1**	**0.968**	0.032	**0.968**	--	--	**0.955**	0.045
Bündtbirne	14-003-843	Büron	15	**G2**	**0.968**	**G2.1**	**0.968**	0.032	**0.968**	--	--	**0.955**	0.045
Luzeiner Dörrbirne	14-003-908	Büron	15	**G2**	**0.968**	**G2.1**	**0.968**	0.032	**0.968**	--	--	**0.955**	0.045
Teigbirne	14-003-619	Aubonne	16	**G2**	0.688	**G2.1**	0.723	0.312	0.688	--	--	0.723	0.277
Gris Chollet	14-003-261	Aclens	16	**G2**	0.688	**G2.1**	0.723	0.312	0.688	--	--	0.723	0.277
Poire Blanc	14-003-1140	Aclens	16	**G2**	0.688	**G2.1**	0.723	0.312	0.688	--	--	0.723	0.277
Poire Blanc	14-003-1537	Aclens	16	**G2**	0.688	**G2.1**	0.723	0.312	0.688	--	--	0.723	0.277
Poire Gris	14-003-1149	Aclens	16	**G2**	0.688	**G2.1**	0.723	0.312	0.688	--	--	0.723	0.277
Poire Blanc	14-003-1115	Pierre-à-bot	16	**G2**	0.688	**G2.1**	0.723	0.312	0.688	--	--	0.723	0.277
Poire Blanc	14-003-1116	Pierre-à-bot	16	**G2**	0.688	**G2.1**	0.723	0.312	0.688	--	--	0.723	0.277
Poire Gris	14-003-263	Pierre-à-bot	16	**G2**	0.688	**G2.1**	0.723	0.312	0.688	--	--	0.723	0.277
Graubirne	14-003-1235	Büron	16	**G2**	0.688	**G2.1**	0.723	0.312	0.688	--	--	0.723	0.277
Poire gris	14-003-1514	Büron	16	**G2**	0.688	**G2.1**	0.723	0.312	0.688	--	--	0.723	0.277
Spalierbirne	14-003-917	Büron	17	**G2**	**0.978**	**G2.1**	**0.978**	0.022	**0.978**	--	--	0.756	0.244
Wildling von Sargans	14-003-672	Knonau	17	**G2**	**0.978**	**G2.1**	**0.978**	0.022	**0.978**	--	--	0.756	0.244
Uelibirne	14-003-1016	Büron	18	**G2**	**0.960**	**G2.1**	**0.960**	0.040	**0.960**	--	--	0.516	0.484
Uelibirne	14-003-636	Knonau	18	**G2**	**0.960**	**G2.1**	**0.960**	0.040	**0.960**	--	--	0.516	0.484
Julieierbirne	14-003-948	Baden	19	**G1**	0.528	**G1.2**	**0.964**	0.528	0.472	0.036	**0.964**	--	--
Butterbirne	14-003-997	Büron	19	**G1**	0.528	**G1.2**	**0.964**	0.528	0.472	0.036	**0.964**	--	--
Unspunnen	14-003-999	Büron	19	**G1**	0.528	**G1.2**	**0.964**	0.528	0.472	0.036	**0.964**	--	--
unknown	14-003-876	Büron	20	**G1**	**0.948**	**G1.1**	**0.968**	**0.948**	0.052	**0.968**	0.032	--	--
Doyenne du Comice	Reference 3	Uni Reading	20	**G1**	**0.948**	**G1.1**	**0.968**	**0.948**	0.052	**0.968**	0.032	--	--
Poire Eyrepire	14-003-2532	Pierre-à-bot	21	**G2**	**0.881**	**G2.1**	**0.881**	0.119	**0.881**	--	--	0.757	0.243
Poire d' Oeuf	14-003-1131	Pierre-à-bot	21	**G2**	**0.881**	**G2.1**	**0.881**	0.119	**0.881**	--	--	0.757	0.243
unknown	14-003-1576	Büron	21	**G2**	**0.881**	**G2.1**	**0.881**	0.119	**0.881**	--	--	0.757	0.243
unknown	14-003-1436	Büron	22	**G2**	**0.967**	**G2.2**	**0.967**	0.033	**0.967**	--	--	0.109	**0.891**
Heubirli	14-003-919	Büron	22	**G2**	**0.967**	**G2.2**	**0.967**	0.033	**0.967**	--	--	0.109	**0.891**
Stadelbirne	14-003-714	Baden	23	**G2**	**0.974**	**G2.2**	**0.974**	0.026	**0.974**	--	--	0.475	0.525
Chrisibira	14-003-1011	Baden	23	**G2**	**0.974**	**G2.2**	**0.974**	0.026	**0.974**	--	--	0.475	0.525
Traubenbirne	14-003-1484	Büron	23	**G2**	**0.974**	**G2.2**	**0.974**	0.026	**0.974**	--	--	0.475	0.525
unknown	14-003-1037	Büron	24	**G2**	**0.967**	**G2.1**	**0.967**	0.033	**0.967**	--	--	0.594	0.406
Dornbirne	14-003-3750	Büron	24	**G2**	**0.967**	**G2.1**	**0.967**	0.033	**0.967**	--	--	0.594	0.406
Hornusser	14-003-861	Büron	25	**G2**	**0.984**	**G2.2**	**0.984**	0.016	**0.984**	--	--	0.167	**0.833**
Längler	14-003-787	Büron	25	**G2**	**0.984**	**G2.2**	**0.984**	0.016	**0.984**	--	--	0.167	**0.833**
Hornuser	14-003-766	Büron	25	**G2**	**0.984**	**G2.2**	**0.984**	0.016	**0.984**	--	--	0.167	**0.833**
Winterbirne	14-003-727	Büron	26	**G1**	**0.923**	**G1.2**	**0.923**	**0.923**	0.077	0.400	0.600	--	--
Poires à Botzi	14-003-1525	Büron	26	**G1**	**0.923**	**G1.2**	**0.923**	**0.923**	0.077	0.400	0.600	--	--
Poire Pouéta Pe	14-003-2530	Pierre-à-bot	27	**G2**	**0.863**	**G2.2**	**0.863**	0.137	**0.863**	--	--	0.401	0.599
Poire Pouta-pi x tapis	14-003-1541	Pierre-à-bot	27	**G2**	**0.863**	**G2.2**	**0.863**	0.137	**0.863**	--	--	0.401	0.599
unknown	14-003-1438	Büron	28	**G1**	**0.947**	**G1.1**	**0.947**	**0.947**	0.053	**0.913**	0.087	--	--
Marie-Luise	14-003-1420	Büron	28	**G1**	**0.947**	**G1.1**	**0.947**	**0.947**	0.053	**0.913**	0.087	--	--
Poire Culotte suisse	14-003-1122	Pierre-à-bot	29	**G1**	**0.915**	**G1.1**	**0.915**	**0.915**	0.085	0.773	0.227	--	--
Schweizerhosen	14-003-753	Büron	29	**G1**	**0.915**	**G1.1**	**0.915**	**0.915**	0.085	0.773	0.227	--	--
Häuffler	14-003-1066	Roggwil Riedern	30	**G2**	**0.830**	**G2.1**	**0.830**	0.170	**0.830**	--	--	0.548	0.452
Stuckibirne	14-003-1045	Baden	30	**G2**	**0.830**	**G2.1**	**0.830**	0.170	**0.830**	--	--	0.548	0.452
Häuffler	14-003-292	Knonau	30	**G2**	**0.830**	**G2.1**	**0.830**	0.170	**0.830**	--	--	0.548	0.452
Dallen-Birne	14-003-1392	Roggwil Riedern	31	**G2**	**0.936**	**G2.2**	**0.936**	0.064	**0.936**	--	--	0.344	0.656
Platzbirne	14-003-1409	Roggwil Riedern	31	**G2**	**0.936**	**G2.2**	**0.936**	0.064	**0.936**	--	--	0.344	0.656
Poire Barbeyron	14-003-26	Aubonne	32	**G1**	0.630	**G1.2**	**0.970**	0.630	0.370	0.030	**0.970**	--	--
Poire Barbeyron	14-003-26	Pierre-à-bot	32	**G1**	0.630	**G1.2**	**0.970**	0.630	0.370	0.030	**0.970**	--	--
Poire de Fer	14-003-1147	Aclens	33	**G2**	**0.915**	**G2.1**	**0.915**	0.085	**0.915**	--	--	0.503	0.497

Chlöpfer	14-003-1031	Baden	33	**G2**	**0.915**	**G2.1**	**0.915**	0.085	**0.915**	--	--	0.503	0.497
Wyy Birne / Weinbirne	14-003-1458	Baden	33	**G2**	**0.915**	**G2.1**	**0.915**	0.085	**0.915**	--	--	0.503	0.497
Martin	14-003-1561	Aclens	34	**G1**	0.548	**G1.2**	**0.966**	0.548	0.452	0.034	**0.966**	--	--
Poire Saint-Martin	14-003-1159	Aclens	34	**G1**	0.548	**G1.2**	**0.966**	0.548	0.452	0.034	**0.966**	--	--
Poire de Fer	14-003-199	Pierre-à-bot	34	**G1**	0.548	**G1.2**	**0.966**	0.548	0.452	0.034	**0.966**	--	--
Poire Quieuchet	14-003-485	Pierre-à-bot	34	**G1**	0.548	**G1.2**	**0.966**	0.548	0.452	0.034	**0.966**	--	--
Poire Saint-Martin	14-003-544	Pierre-à-bot	34	**G1**	0.548	**G1.2**	**0.966**	0.548	0.452	0.034	**0.966**	--	--
Poire Cent-Grappes rouge	14-003-108	Pierre-à-bot	35	**G1**	0.737	**G1.2**	0.754	0.737	0.263	0.246	0.754	--	--
unknown	14-003-1474	Büron	35	**G1**	0.737	**G1.2**	0.754	0.737	0.263	0.246	0.754	--	--
Gränggeli	14-003-1424	Baden	36	**G2**	0.789	**G2.2**	0.789	0.211	0.789	--	--	0.303	0.697
Grängeli	14-003-336	Knonau	36	**G2**	0.789	**G2.2**	0.789	0.211	0.789	--	--	0.303	0.697
Nidwaldner Weissbirne	14-003-710	Baden	37	**G2**	**0.800**	**G2.1**	**0.800**	0.200	**0.800**	--	--	0.585	0.415
Lehnbirne	14-003-704	Baden	37	**G2**	**0.800**	**G2.1**	**0.800**	0.200	**0.800**	--	--	0.585	0.415
Süssbirli	14-003-1413	Büron	38	**G2**	0.664	**G2.2**	0.770	0.336	0.664	--	--	0.230	0.770
Büschelibirli	14-003-1412	Büron	38	**G2**	0.664	**G2.2**	0.770	0.336	0.664	--	--	0.230	0.770
Pero Breno	14-003-2574	Manno	39	**G1**	0.569	**G1.2**	**0.883**	0.569	0.431	0.117	**0.883**	--	--
Pér bütér	14-003-2580	Manno	39	**G1**	0.569	**G1.2**	**0.883**	0.569	0.431	0.117	**0.883**	--	--
Sonnenbirne	14-003-1337	Koppigen	40	**G2**	**0.935**	**G2.1**	**0.948**	0.065	**0.935**	--	--	**0.948**	0.052
unknown	14-003-1223	Koppigen	40	**G2**	**0.935**	**G2.1**	**0.948**	0.065	**0.935**	--	--	**0.948**	0.052
Poire Bellexime	14-003-40	Pierre-à-bot	41	**G1**	0.596	**G1.2**	**0.894**	0.596	0.404	0.106	**0.894**	--	--
Poire Cuisse-Dame	14-003-155	Pierre-à-bot	41	**G1**	0.596	**G1.2**	**0.894**	0.596	0.404	0.106	**0.894**	--	--
Poire Monsieur	14-003-414	Pierre-à-bot	41	**G1**	0.596	**G1.2**	**0.894**	0.596	0.404	0.106	**0.894**	--	--
Poire Tabatière	14-003-616	Pierre-à-bot	41	**G1**	0.596	**G1.2**	**0.894**	0.596	0.404	0.106	**0.894**	--	--
Nägeli	14-003-1053	Büron	41	**G1**	0.596	**G1.2**	**0.894**	0.596	0.404	0.106	**0.894**	--	--
Nägelibirne	14-003-427	Dürrenäsch	41	**G1**	0.596	**G1.2**	**0.894**	0.596	0.404	0.106	**0.894**	--	--
Poire Socratte	14-003-577	Pierre-à-bot	42	**G2**	**0.871**	**G2.2**	**0.871**	0.129	**0.871**	--	--	0.319	0.681
Poire à Soupe	14-003-1135	Pierre-à-bot	42	**G2**	**0.871**	**G2.2**	**0.871**	0.129	**0.871**	--	--	0.319	0.681
Sconosciuto prob. locale	14-003-2585	Manno	43	**G1**	0.508	**G1.2**	**0.912**	0.508	0.492	0.088	**0.912**	--	--
Pér de Sant'Ana	14-003-2589	Manno	43	**G1**	0.508	**G1.2**	**0.912**	0.508	0.492	0.088	**0.912**	--	--
Züribirä	14-003-982	Büron	44	**G1**	**0.975**	**G1.1**	**0.975**	**0.975**	0.025	0.737	0.263	--	--
Wieserbirne	14-003-303	Büron	44	**G1**	**0.975**	**G1.1**	**0.975**	**0.975**	0.025	0.737	0.263	--	--
Schweizerhose	14-003-988	Roggwil Riedern	45	**G2**	**0.958**	**G2.1**	**0.958**	0.042	**0.958**	--	--	**0.956**	0.044
unknown	14-003-1364	Koppigen	45	**G2**	**0.958**	**G2.1**	**0.958**	0.042	**0.958**	--	--	**0.956**	0.044
Sunnebire	14-003-933	Büron	45	**G2**	**0.958**	**G2.1**	**0.958**	0.042	**0.958**	--	--	**0.956**	0.044
Schwyzerhose	14-003-995	Büron	45	**G2**	**0.958**	**G2.1**	**0.958**	0.042	**0.958**	--	--	**0.956**	0.044
Längler	14-003-355	Roggwil Riedern	46	**G2**	**0.966**	**G2.1**	**0.966**	0.034	**0.966**	--	--	**0.941**	0.059
Wadelbirne	14-003-651	Aclens	46	**G2**	**0.966**	**G2.1**	**0.966**	0.034	**0.966**	--	--	**0.941**	0.059
Poire Cannepire	14-003-101	Pierre-à-bot	46	**G2**	**0.966**	**G2.1**	**0.966**	0.034	**0.966**	--	--	**0.941**	0.059
Poire Jaune Codia	14-003-129	Pierre-à-bot	46	**G2**	**0.966**	**G2.1**	**0.966**	0.034	**0.966**	--	--	**0.941**	0.059
Poire Coiièdge	14-003-130	Pierre-à-bot	46	**G2**	**0.966**	**G2.1**	**0.966**	0.034	**0.966**	--	--	**0.941**	0.059
Poire Colliard	14-003-134	Pierre-à-bot	46	**G2**	**0.966**	**G2.1**	**0.966**	0.034	**0.966**	--	--	**0.941**	0.059
Poire à Golliard	14-003-249	Pierre-à-bot	46	**G2**	**0.966**	**G2.1**	**0.966**	0.034	**0.966**	--	--	**0.941**	0.059
Comtesse de Paris	14-003-136	Roggwil Hofen	47	**G1**	**0.844**	**G1.2**	**0.844**	**0.844**	0.156	0.256	0.744	--	--
Comtesse de Paris	14-003-136	Pierre-à-bot	47	**G1**	**0.844**	**G1.2**	**0.844**	**0.844**	0.156	0.256	0.744	--	--
Affelträngler	14-003-6	Roggwil Hofen	48	**G2**	**0.812**	**G2.2**	**0.849**	0.188	**0.812**	--	--	0.151	**0.849**
Bändlibirne	14-003-1051	Baden	48	**G2**	**0.812**	**G2.2**	**0.849**	0.188	**0.812**	--	--	0.151	**0.849**
Ryschacherbirne	14-003-1065	Baden	48	**G2**	**0.812**	**G2.2**	**0.849**	0.188	**0.812**	--	--	0.151	**0.849**
kleine Birli	14-003-1018	Baden	48	**G2**	**0.812**	**G2.2**	**0.849**	0.188	**0.812**	--	--	0.151	**0.849**
Affelträngler	14-003-6	Baden	48	**G2**	**0.812**	**G2.2**	**0.849**	0.188	**0.812**	--	--	0.151	**0.849**
Affeltranger	14-003-1069	Büron	48	**G2**	**0.812**	**G2.2**	**0.849**	0.188	**0.812**	--	--	0.151	**0.849**
Trischeläbere	14-003-763	Roggwil Hofen	49	**G2**	**0.863**	**G2.1**	**0.863**	0.137	**0.863**	--	--	0.694	0.306
Mokholzbirne	14-003-763	Büron	49	**G2**	**0.863**	**G2.1**	**0.863**	0.137	**0.863**	--	--	0.694	0.306
Goldschmeckler	14-003-253	Roggwil Hofen	50	**G2**	**0.984**	**G2.2**	**0.984**	0.016	**0.984**	--	--	0.355	0.645
Bergbirne	14-003-664	Baden	50	**G2**	**0.984**	**G2.2**	**0.984**	0.016	**0.984**	--	--	0.355	0.645
Bergbirne	14-003-914	Büron	50	**G2**	**0.984**	**G2.2**	**0.984**	0.016	**0.984**	--	--	0.355	0.645
Späte Weinbirne	14-003-805	Büron	50	**G2**	**0.984**	**G2.2**	**0.984**	0.016	**0.984**	--	--	0.355	0.645
Aeschmer Holzbirne	14-003-5	Knonau	50	**G2**	**0.984**	**G2.2**	**0.984**	0.016	**0.984**	--	--	0.355	0.645
Lätterbirne	14-003-358	Roggwil Hofen	51	**G2**	**0.939**	**G2.1**	**0.939**	0.061	**0.939**	--	--	0.749	0.251
Stuttgarter Weinbirne	14-003-765	Büron	51	**G2**	**0.939**	**G2.1**	**0.939**	0.061	**0.939**	--	--	0.749	0.251
Schellerbirne	14-003-554	Aubonne	52	**G2**	0.716	**G2.2**	**0.856**	0.284	0.716	--	--	0.144	**0.856**
Poire Bataillard	14-003-33	Pierre-à-bot	52	**G2**	0.716	**G2.2**	**0.856**	0.284	0.716	--	--	0.144	**0.856**
Cuisinière	14-003-1553	Pierre-à-bot	52	**G2**	0.716	**G2.2**	**0.856**	0.284	0.716	--	--	0.144	**0.856**
Poire Gaucher	14-003-232	Pierre-à-bot	52	**G2**	0.716	**G2.2**	**0.856**	0.284	0.716	--	--	0.144	**0.856**
Poire Caillin	14-003-95	Pierre-à-bot	53	**G2**	**0.894**	**G2.2**	**0.894**	0.106	**0.894**	--	--	0.266	0.734
unknown	14-003-1447	Baden	53	**G2**	**0.894**	**G2.2**	**0.894**	0.106	**0.894**	--	--	0.266	0.734
Büschelibirä	14-003-1446	Baden	54	**G1**	**0.939**	**G1.2**	**0.939**	**0.939**	0.061	0.483	0.517	--	--
Winterroussellette	14-003-975	Büron	54	**G1**	**0.939**	**G1.2**	**0.939**	**0.939**	0.061	0.483	0.517	--	--
Büschelibirne	14-003-915	Büron	54	**G1**	**0.939**	**G1.2**	**0.939**	**0.939**	0.061	0.483	0.517	--	--
Basler Birli	14-003-1025	Büron	55	**G2**	**0.979**	**G2.1**	**0.979**	0.021	**0.979**	--	--	0.513	0.487
unknown	14-003-1482	Büron	55	**G2**	**0.979**	**G2.1**	**0.979**	0.021	**0.979**	--	--	0.513	0.487
Pero Lopagno	14-003-2575	Manno	56	**G1**	**0.936**	**G1.1**	**0.936**	**0.936**	0.064	0.722	0.278	--	--
Per buter Bioggio	14-003-2695	Manno	56	**G1**	**0.936**	**G1.1**	**0.936**	**0.936**	0.064	0.722	0.278	--	--
Pere farinose / Pere grandi	14-003-1506	Roggwil Riedern	56	**G1**	**0.936**	**G1.1**	**0.936**	**0.936**	0.064	0.722	0.278	--	--
Pero Decana (lato nord)	14-003-2736	Manno	57	**G1**	**0.941**	**G1.1**	**0.941**	**0.941**	0.059	**0.879**	0.121	--	--
Butterbirne	14-003-1302	Büron	57	**G1**	**0.941**	**G1.1**	**0.941**	**0.941**	0.059	**0.879**	0.121	--	--
Pero Decana (latu sud)	14-003-2735	Manno	58	**G1**	0.560	**G1.1**	0.672	0.560	0.440	0.672	0.328	--	--
Deutsche National Bergamotte	14-003-165	Roggwil Hofen	58	**G1**	0.560	**G1.1**	0.672	0.560	0.440	0.672	0.328	--	--
Bergamotte	14-003-1425	Baden	58	**G1**	0.560	**G1.1**	0.672	0.560	0.440	0.672	0.328	--	--
Tellerbirne	14-003-722	Baden	58	**G1**	0.560	**G1.1**	0.672	0.560	0.440	0.672	0.328	--	--
Glockenbirne	14-003-752	Baden	58	**G1**	0.560	**G1.1**	0.672	0.560	0.440	0.672	0.328	--	--
unknown / Wettinger Holzbirne	14-003-1402	Roggwil Riedern	59	**G2**	**0.952**	**G2.2**	**0.952**	0.048	**0.952**	--	--	0.390	0.610
Muggenbirne	14-003-905	Büron	59	**G2**	**0.952**	**G2.2**	**0.952**	0.048	**0.952**	--	--	0.390	0.610
Schiblerbirne	14-003-922	Büron	59	**G2**	**0.952**	**G2.2**	**0.952**	0.048	**0.952**	--	--	0.390	0.610
Biessenhofer Mostbirne	14-003-58	Büron	59	**G2**	**0.952**	**G2.2**	**0.952**	0.048	**0.952**	--	--	0.390	0.610
Ballaigui	14-003-24	Aubonne	60	**G1**	0.593	**G1.1**	0.593	0.593	0.407	0.546	0.454	--	--
Poire Bergamote de Ballaigues	14-003-1137	Pierre-à-bot	60	**G1**	0.593	**G1.1**	0.593	0.593	0.407	0.546	0.454	--	--
Poire Demi-Goillard	14-003-162	Pierre-à-bot	60	**G1**	0.593	**G1.1**	0.593	0.593	0.407	0.546	0.454	--	--

Gros brun	14-003-2513	Aubonne	61	**G1**	0.594	**G1.2**	0.694	0.594	0.406	0.306	0.694	--	--
Poire-Livre	14-003-1129	Pierre-à-bot	61	**G1**	0.594	**G1.2**	0.694	0.594	0.406	0.306	0.694	--	--
Poire Vouarding	14-003-1136	Pierre-à-bot	61	**G1**	0.594	**G1.2**	0.694	0.594	0.406	0.306	0.694	--	--
Poire Fondante des Bois	14-003-214	Pierre-à-bot	62	**G1**	**0.982**	**G1.1**	**0.982**	**0.982**	0.018	**0.944**	0.056	--	--
Winterbirne	14-003-1492	Büron	62	**G1**	**0.982**	**G1.1**	**0.982**	**0.982**	0.018	**0.944**	0.056	--	--
Braune Birne	14-003-837	Büron	62	**G1**	**0.982**	**G1.1**	**0.982**	**0.982**	0.018	**0.944**	0.056	--	--
Poire Rosselet	14-003-518	Pierre-à-bot	63	**G1**	**0.932**	**G1.2**	**0.937**	**0.932**	0.068	0.063	**0.937**	--	--
Deutsche Kanne	14-003-1315	Baden	63	**G1**	**0.932**	**G1.2**	**0.937**	**0.932**	0.068	0.063	**0.937**	--	--
unknown	14-003-1331	Baden	63	**G1**	**0.932**	**G1.2**	**0.937**	**0.932**	0.068	0.063	**0.937**	--	--
unknown	14-003-587	Büron	63	**G1**	**0.932**	**G1.2**	**0.937**	**0.932**	0.068	0.063	**0.937**	--	--
Kleines Zuckerbirrli	14-003-912	Büron	63	**G1**	**0.932**	**G1.2**	**0.937**	**0.932**	0.068	0.063	**0.937**	--	--
unknown	14-003-1463	Büron	63	**G1**	**0.932**	**G1.2**	**0.937**	**0.932**	0.068	0.063	**0.937**	--	--
Stuttgarter Geishirtle	14-003-604	Knonau	63	**G1**	**0.932**	**G1.2**	**0.937**	**0.932**	0.068	0.063	**0.937**	--	--
Melonenbirne	14-003-788	Baden	64	**G2**	**0.945**	**G2.1**	**0.945**	0.055	**0.945**	--	--	0.797	0.203
Reiholzer	14-003-1373	Büron	64	**G2**	**0.945**	**G2.1**	**0.945**	0.055	**0.945**	--	--	0.797	0.203
Melonenbirne	14-003-918	Büron	64	**G2**	**0.945**	**G2.1**	**0.945**	0.055	**0.945**	--	--	0.797	0.203
Madame Verté	14-003-382	Roggwil Hofen	65	**G2**	**0.973**	**G2.2**	**0.973**	0.027	**0.973**	--	--	0.171	**0.829**
Reinholzbirne	14-003-498	Roggwil Hofen	65	**G2**	**0.973**	**G2.2**	**0.973**	0.027	**0.973**	--	--	0.171	**0.829**
Wildling von Einsiedeln	14-003-2520	Aubonne	65	**G2**	**0.973**	**G2.2**	**0.973**	0.027	**0.973**	--	--	0.171	**0.829**
Cothio	14-003-1496	Roggwil Riedern	66	**G2**	**0.853**	**G2.2**	**0.853**	0.147	**0.853**	--	--	0.279	0.721
Poiratte	14-003-1497	Aclens	66	**G2**	**0.853**	**G2.2**	**0.853**	0.147	**0.853**	--	--	0.279	0.721
Poire à Cotio	14-003-141	Pierre-à-bot	66	**G2**	**0.853**	**G2.2**	**0.853**	0.147	**0.853**	--	--	0.279	0.721
Poire Courbe	14-003-145	Pierre-à-bot	66	**G2**	**0.853**	**G2.2**	**0.853**	0.147	**0.853**	--	--	0.279	0.721
Glockenbirne	14-003-1040	Roggwil Riedern	67	**G2**	**0.974**	**G2.1**	**0.974**	0.026	**0.974**	--	--	0.739	0.261
Speckbirne	14-003-798	Roggwil Riedern	67	**G2**	**0.974**	**G2.1**	**0.974**	0.026	**0.974**	--	--	0.739	0.261
Elmenshofer	14-003-1020	Roggwil Riedern	67	**G2**	**0.974**	**G2.1**	**0.974**	0.026	**0.974**	--	--	0.739	0.261
Breitbirne	14-003-1034	Büron	67	**G2**	**0.974**	**G2.1**	**0.974**	0.026	**0.974**	--	--	0.739	0.261
Weinbirne	14-003-762	Büron	67	**G2**	**0.974**	**G2.1**	**0.974**	0.026	**0.974**	--	--	0.739	0.261
Speckbirne	14-003-834	Büron	67	**G2**	**0.974**	**G2.1**	**0.974**	0.026	**0.974**	--	--	0.739	0.261
Lümbergsbirne	14-003-1379	Roggwil Riedern	68	**G2**	**0.954**	**G2.2**	**0.954**	0.046	**0.954**	--	--	0.326	0.674
unknown	14-003-1371	Baden	68	**G2**	**0.954**	**G2.2**	**0.954**	0.046	**0.954**	--	--	0.326	0.674
Niederländer	14-003-1391	Büron	68	**G2**	**0.954**	**G2.2**	**0.954**	0.046	**0.954**	--	--	0.326	0.674
Meitlibirne	14-003-1381	Büron	68	**G2**	**0.954**	**G2.2**	**0.954**	0.046	**0.954**	--	--	0.326	0.674
Thalbirne	14-003-1393	Büron	68	**G2**	**0.954**	**G2.2**	**0.954**	0.046	**0.954**	--	--	0.326	0.674
Zuckerbirne	14-003-810	Roggwil Riedern	69	**G1**	**0.918**	**G1.1**	**0.918**	**0.918**	0.082	**0.822**	0.178	--	--
Goldlänglerbirne	14-003-846	Roggwil Riedern	69	**G1**	**0.918**	**G1.1**	**0.918**	**0.918**	0.082	**0.822**	0.178	--	--
Blaulängala	14-003-906	Roggwil Hofen	70	**G2**	**0.974**	**G2.1**	**0.974**	0.026	**0.974**	--	--	0.647	0.353
Römische Schmalzbirne	14-003-906	Roggwil Hofen	70	**G2**	**0.974**	**G2.1**	**0.974**	0.026	**0.974**	--	--	0.647	0.353
Zuckerbirne	14-003-885	Büron	70	**G2**	**0.974**	**G2.1**	**0.974**	0.026	**0.974**	--	--	0.647	0.353
unknown	14-003-927	Büron	70	**G2**	**0.974**	**G2.1**	**0.974**	0.026	**0.974**	--	--	0.647	0.353
Römische Schmalzbirne	14-003-840	Büron	70	**G2**	**0.974**	**G2.1**	**0.974**	0.026	**0.974**	--	--	0.647	0.353
Blaulängala	14-003-906	Büron	70	**G2**	**0.974**	**G2.1**	**0.974**	0.026	**0.974**	--	--	0.647	0.353
Schweizer Bratbirne	14-003-564	Roggwil Hofen	71	**G2**	**0.973**	**G2.1**	**0.973**	0.027	**0.973**	--	--	0.593	0.407
Imberwurzen	14-003-317	Knonau	71	**G2**	**0.973**	**G2.1**	**0.973**	0.027	**0.973**	--	--	0.593	0.407
Poire Aberiet	14-003-1139	Aclens	72	**G2**	0.612	**G2.2**	0.762	0.388	0.612	--	--	0.238	0.762
Aberiet	14-003-1535	Aclens	72	**G2**	0.612	**G2.2**	0.762	0.388	0.612	--	--	0.238	0.762
Poire Aberiet	14-003-2	Pierre-à-bot	72	**G2**	0.612	**G2.2**	0.762	0.388	0.612	--	--	0.238	0.762
Poire-Fer	14-003-1145	Aclens	73	**G2**	0.540	**G2.2**	0.677	0.460	0.540	--	--	0.323	0.677
Poire Rêche	14-003-493	Aclens	73	**G2**	0.540	**G2.2**	0.677	0.460	0.540	--	--	0.323	0.677
Poire rèche	14-003-1579	Aclens	73	**G2**	0.540	**G2.2**	0.677	0.460	0.540	--	--	0.323	0.677
Poire Rêche	14-003-492	Pierre-à-bot	73	**G2**	0.540	**G2.2**	0.677	0.460	0.540	--	--	0.323	0.677
Poire à Rissoles	14-003-1570	Aclens	74.1	**G1**	**0.972**	**G1.1**	**0.972**	**0.972**	0.028	**0.967**	0.033	--	--
Conférence	14-003-2536	Pierre-à-bot	74.1	**G1**	**0.972**	**G1.1**	**0.972**	**0.972**	0.028	**0.967**	0.033	--	--
Zuckerbirne / Conference	14-003-1068	Baden	74.1	**G1**	**0.972**	**G1.1**	**0.972**	**0.972**	0.028	**0.967**	0.033	--	--
unknown	14-003-1462	Büron	74.1	**G1**	**0.972**	**G1.1**	**0.972**	**0.972**	0.028	**0.967**	0.033	--	--
unknown	14-003-1395	Büron	74.1	**G1**	**0.972**	**G1.1**	**0.972**	**0.972**	0.028	**0.967**	0.033	--	--
Conference	Reference 4	Uni Reading	74.2	**G1**	**0.948**	**G1.1**	**0.968**	**0.948**	0.052	**0.968**	0.032	--	--
Tyrola / Tyroler	14-003-1510	Baden	75	**G2**	**0.917**	**G2.1**	**0.917**	0.083	**0.917**	--	--	**0.826**	0.174
Schmalzbirne von Brest	14-003-557	Büron	75	**G2**	**0.917**	**G2.1**	**0.917**	0.083	**0.917**	--	--	**0.826**	0.174
Poire des moissons	14-003-1499	Pierre-à-bot	76	**G1**	**0.904**	**G1.2**	**0.904**	**0.904**	0.096	0.108	**0.892**	--	--
Poire de moisson	14-003-1499	Büron	76	**G1**	**0.904**	**G1.2**	**0.904**	**0.904**	0.096	0.108	**0.892**	--	--
Graue Birne	14-003-1104	Baden	77	**G1**	0.671	**G1.2**	**0.962**	0.671	0.329	0.038	**0.962**	--	--
Graue Birne	14-003-262	Knonau	77	**G1**	0.671	**G1.2**	**0.962**	0.671	0.329	0.038	**0.962**	--	--
Gugerlibirne	14-003-278	Baden	78	**G1**	**0.973**	**G1.1**	**0.979**	**0.973**	0.027	**0.979**	0.021	--	--
Weisse Herbstbutterbirne	14-003-1256	Baden	78	**G1**	**0.973**	**G1.1**	**0.979**	**0.973**	0.027	**0.979**	0.021	--	--
unknown	14-003-1485	Büron	78	**G1**	**0.973**	**G1.1**	**0.979**	**0.973**	0.027	**0.979**	0.021	--	--
Pero squisito	14-003-2697	Manno	79	**G1**	**0.942**	**G1.2**	**0.942**	**0.942**	0.058	0.132	**0.868**	--	--
Pero rossiccio	14-003-2706	Manno	79	**G1**	**0.942**	**G1.2**	**0.942**	**0.942**	0.058	0.132	**0.868**	--	--
Sept en gueule	14-003-1571	Aclens	79	**G1**	**0.942**	**G1.2**	**0.942**	**0.942**	0.058	0.132	**0.868**	--	--
Poire gris	14-003-1536	Aclens	79	**G1**	**0.942**	**G1.2**	**0.942**	**0.942**	0.058	0.132	**0.868**	--	--
Poire des Moissons	14-003-1575	Aclens	79	**G1**	**0.942**	**G1.2**	**0.942**	**0.942**	0.058	0.132	**0.868**	--	--
Poire à Rissole	14-003-1157	Aclens	79	**G1**	**0.942**	**G1.2**	**0.942**	**0.942**	0.058	0.132	**0.868**	--	--
Poire Roh	14-003-1558	Aclens	79	**G1**	**0.942**	**G1.2**	**0.942**	**0.942**	0.058	0.132	**0.868**	--	--
Laurent	14-003-1551	Aclens	79	**G1**	**0.942**	**G1.2**	**0.942**	**0.942**	0.058	0.132	**0.868**	--	--
Poire Beurré Gris	14-003-53	Pierre-à-bot	79	**G1**	**0.942**	**G1.2**	**0.942**	**0.942**	0.058	0.132	**0.868**	--	--
Poire Blesson rouge de Ballaigues	14-003-1117	Pierre-à-bot	79	**G1**	**0.942**	**G1.2**	**0.942**	**0.942**	0.058	0.132	**0.868**	--	--
Poire Canéet	14-003-1119	Pierre-à-bot	79	**G1**	**0.942**	**G1.2**	**0.942**	**0.942**	0.058	0.132	**0.868**	--	--
Poire Fricot	14-003-1552	Pierre-à-bot	79	**G1**	**0.942**	**G1.2**	**0.942**	**0.942**	0.058	0.132	**0.868**	--	--
Poir Gägubirli	14-003-1515	Pierre-à-bot	79	**G1**	**0.942**	**G1.2**	**0.942**	**0.942**	0.058	0.132	**0.868**	--	--
Poire Olivette	14-003-437	Pierre-à-bot	79	**G1**	**0.942**	**G1.2**	**0.942**	**0.942**	0.058	0.132	**0.868**	--	--
Poire Rousselette	14-003-531	Pierre-à-bot	79	**G1**	**0.942**	**G1.2**	**0.942**	**0.942**	0.058	0.132	**0.868**	--	--
Poire Wasserbirne	14-003-1191	Pierre-à-bot	79	**G1**	**0.942**	**G1.2**	**0.942**	**0.942**	0.058	0.132	**0.868**	--	--
Poire Hasebirne	14-003-2533	Pierre-à-bot	79	**G1**	**0.942**	**G1.2**	**0.942**	**0.942**	0.058	0.132	**0.868**	--	--
Poire Steinmüribirne	14-003-594	Pierre-à-bot	79	**G1**	**0.942**	**G1.2**	**0.942**	**0.942**	0.058	0.132	**0.868**	--	--
unknown	14-003-1452	Büron	79	**G1**	**0.942**	**G1.2**	**0.942**	**0.942**	0.058	0.132	**0.868**	--	--
unknown	14-003-1451	Büron	79	**G1**	**0.942**	**G1.2**	**0.942**	**0.942**	0.058	0.132	**0.868**	--	--
Rebenbirne	14-003-490	Knonau	79	**G1**	**0.942**	**G1.2**	**0.942**	**0.942**	0.058	0.132	**0.868**	--	--

Pisö di Vescràa	14-003-2714	Manno	80	**G1**	0.797	**G1.2**	**0.928**	0.797	0.203	0.072	**0.928**	--	--
Poire Ostan	14-003-442	Pierre-à-bot	80	**G1**	0.797	**G1.2**	**0.928**	0.797	0.203	0.072	**0.928**	--	--
Poire Pape	14-003-448	Pierre-à-bot	80	**G1**	0.797	**G1.2**	**0.928**	0.797	0.203	0.072	**0.928**	--	--
Pape	14-003-1550	Büron	80	**G1**	0.797	**G1.2**	**0.928**	0.797	0.203	0.072	**0.928**	--	--
Weisse Welschbirne	14-003-746	Roggwil Riedern	81	**G2**	0.712	**G2.1**	0.712	0.288	0.712	--	--	0.596	0.404
Champagner	14-003-666	Büron	81	**G2**	0.712	**G2.1**	0.712	0.288	0.712	--	--	0.596	0.404
Dornbirne	14-003-1320	Koppigen	82	**G1**	0.708	**G1.2**	0.795	0.708	0.292	0.205	0.795	--	--
unknown	14-003-1217	Koppigen	82	**G1**	0.708	**G1.2**	0.795	0.708	0.292	0.205	0.795	--	--
Ulmer Butterbirne	14-003-637	Roggwil Hofen	83	**G1**	**0.913**	**G1.1**	**0.913**	**0.913**	0.087	**0.903**	0.097	--	--
Langstiel	14-003-1375	Baden	83	**G1**	**0.913**	**G1.1**	**0.913**	**0.913**	0.087	**0.903**	0.097	--	--
Schürbirne	14-003-560	Aubonne	84	**G2**	**0.969**	**G2.2**	**0.969**	0.031	**0.969**	--	--	0.440	0.560
Poire Epine	14-003-1521	Aclens	84	**G2**	**0.969**	**G2.2**	**0.969**	0.031	**0.969**	--	--	0.440	0.560
Poire Epine	14-003-187	Pierre-à-bot	84	**G2**	**0.969**	**G2.2**	**0.969**	0.031	**0.969**	--	--	0.440	0.560
Épine	14-003-1521	Pierre-à-bot	84	**G2**	**0.969**	**G2.2**	**0.969**	0.031	**0.969**	--	--	0.440	0.560
Fernate d'hiver	14-003-2509	Aubonne	85	**G1**	0.718	**G1.2**	**0.815**	0.718	0.282	0.185	**0.815**	--	--
Poire Fernatte d'hiver	14-003-201	Pierre-à-bot	85	**G1**	0.718	**G1.2**	**0.815**	0.718	0.282	0.185	**0.815**	--	--
Poire farine	14-003-2515	Aubonne	86	**G2**	**0.802**	**G2.2**	**0.802**	0.198	**0.802**	--	--	0.411	0.589
Poire Fernattes	14-003-1498	Aclens	86	**G2**	**0.802**	**G2.2**	**0.802**	0.198	**0.802**	--	--	0.411	0.589
Poire Fernatte	14-003-200	Pierre-à-bot	86	**G2**	**0.802**	**G2.2**	**0.802**	0.198	**0.802**	--	--	0.411	0.589
Fernatte	14-003-1504	Pierre-à-bot	86	**G2**	**0.802**	**G2.2**	**0.802**	0.198	**0.802**	--	--	0.411	0.589
Poire Fernotte	14-003-202	Pierre-à-bot	86	**G2**	**0.802**	**G2.2**	**0.802**	0.198	**0.802**	--	--	0.411	0.589
Poire Mehlbirne	14-003-398	Pierre-à-bot	86	**G2**	**0.802**	**G2.2**	**0.802**	0.198	**0.802**	--	--	0.411	0.589
Blesson dur	14-003-2500	Aubonne	87	**G1**	0.560	**G1.2**	**0.896**	0.560	0.440	0.104	**0.896**	--	--
Poire Prodige	14-003-59	Pierre-à-bot	87	**G1**	0.560	**G1.2**	**0.896**	0.560	0.440	0.104	**0.896**	--	--
Poire Bellosin	14-003-2528	Aclens	88	**G2**	0.609	**G2.2**	0.673	0.391	0.609	--	--	0.327	0.673
Poire Rogin	14-003-509	Aclens	88	**G2**	0.609	**G2.2**	0.673	0.391	0.609	--	--	0.327	0.673
Poire Bacon	14-003-22	Pierre-à-bot	89	**G1**	**0.969**	**G1.1**	**0.969**	**0.969**	0.031	0.699	0.301	--	--
Poire Muscat	14-003-420	Pierre-à-bot	89	**G1**	**0.969**	**G1.1**	**0.969**	**0.969**	0.031	0.699	0.301	--	--
Poire Noir	14-003-431	Pierre-à-bot	90	**G2**	**0.950**	**G2.2**	**0.950**	0.050	**0.950**	--	--	0.347	0.653
unknown	14-003-739	Büron	90	**G2**	**0.950**	**G2.2**	**0.950**	0.050	**0.950**	--	--	0.347	0.653
Braunbirne	14-003-723	Roggwil Riedern	91	**G1**	0.719	**G1.1**	0.719	0.719	0.281	0.639	0.361	--	--
Sonnenbirne	14-003-1449	Roggwil Riedern	91	**G1**	0.719	**G1.1**	0.719	0.719	0.281	0.639	0.361	--	--
Muskatelter	14-003-705	Baden	91	**G1**	0.719	**G1.1**	0.719	0.719	0.281	0.639	0.361	--	--
Grande queue	14-003-1563	Aclens	92	**G2**	**0.951**	**G2.2**	**0.951**	0.049	**0.951**	--	--	0.488	0.512
Poire Epine	14-003-188	Pierre-à-bot	92	**G2**	**0.951**	**G2.2**	**0.951**	0.049	**0.951**	--	--	0.488	0.512
Mutschgenteller	14-003-1252	Baden	92	**G2**	**0.951**	**G2.2**	**0.951**	0.049	**0.951**	--	--	0.488	0.512
Rote Muskateller	14-003-523	Knonau	92	**G2**	**0.951**	**G2.2**	**0.951**	0.049	**0.951**	--	--	0.488	0.512
Fleischbirne	14-003-1029	Roggwil Riedern	93.1	**G2**	0.679	**G2.1**	0.771	0.321	0.679	--	--	0.771	0.229
Späte Hansli	14-003-1351	Büron	93.1	**G2**	0.679	**G2.1**	0.771	0.321	0.679	--	--	0.771	0.229
Butterbirne	14-003-1321	Roggwil Riedern	93.2	**G1**	**0.896**	**G1.1**	**0.896**	**0.896**	0.104	0.756	0.244	--	--
Fuchsbirne	14-003-830	Roggwil Riedern	94	**G1**	**0.977**	**G1.1**	**0.984**	**0.977**	0.023	**0.984**	0.016	--	--
unknown	14-003-1439	Büron	94	**G1**	**0.977**	**G1.1**	**0.984**	**0.977**	0.023	**0.984**	0.016	--	--
unknown	14-003-1529	Büron	94	**G1**	**0.977**	**G1.1**	**0.984**	**0.977**	0.023	**0.984**	0.016	--	--
unknown	14-003-1459	Büron	94	**G1**	**0.977**	**G1.1**	**0.984**	**0.977**	0.023	**0.984**	0.016	--	--
Turgauer Weinbirnen	14-003-1376	Roggwil Riedern	95	**G2**	**0.949**	**G2.1**	**0.949**	0.051	**0.949**	--	--	0.511	0.489
Gunterzhauser	14-003-741	Roggwil Riedern	95	**G2**	**0.949**	**G2.1**	**0.949**	0.051	**0.949**	--	--	0.511	0.489
unknown	14-003-1457	Roggwil Riedern	95	**G2**	**0.949**	**G2.1**	**0.949**	0.051	**0.949**	--	--	0.511	0.489
Ottenbacher Schellerbirne	14-003-444	Roggwil Hofen	95	**G2**	**0.949**	**G2.1**	**0.949**	0.051	**0.949**	--	--	0.511	0.489
Glockenbirne	14-003-247	Pierre-à-bot	95	**G2**	**0.949**	**G2.1**	**0.949**	0.051	**0.949**	--	--	0.511	0.489
Widilämpä	14-003-989	Büron	95	**G2**	**0.949**	**G2.1**	**0.949**	0.051	**0.949**	--	--	0.511	0.489
Winterbirne	14-003-811	Büron	95	**G2**	**0.949**	**G2.1**	**0.949**	0.051	**0.949**	--	--	0.511	0.489
unknown	14-003-1432	Büron	95	**G2**	**0.949**	**G2.1**	**0.949**	0.051	**0.949**	--	--	0.511	0.489
unknown	14-003-1493	Büron	95	**G2**	**0.949**	**G2.1**	**0.949**	0.051	**0.949**	--	--	0.511	0.489
unknown	14-003-1429	Roggwil Riedern	96	**G2**	**0.979**	**G2.1**	**0.979**	0.021	**0.979**	--	--	0.771	0.229
Forellenbirne	14-003-215	Koppigen	96	**G2**	**0.979**	**G2.1**	**0.979**	0.021	**0.979**	--	--	0.771	0.229
Hanslibirne	14-003-288	Roggwil Hofen	96	**G2**	**0.979**	**G2.1**	**0.979**	0.021	**0.979**	--	--	0.771	0.229
unknown	14-003-1339	Baden	96	**G2**	**0.979**	**G2.1**	**0.979**	0.021	**0.979**	--	--	0.771	0.229
Zuckerbirne	14-003-777	Büron	96	**G2**	**0.979**	**G2.1**	**0.979**	0.021	**0.979**	--	--	0.771	0.229
Grünbirne	14-003-1273	Koppigen	97	**G2**	**0.800**	**G2.1**	**0.800**	0.200	**0.800**	--	--	0.546	0.454
Bruembirne / Wörgibire	14-003-1262	Koppigen	97	**G2**	**0.800**	**G2.1**	**0.800**	0.200	**0.800**	--	--	0.546	0.454
Hengeln	14-003-1289	Koppigen	97	**G2**	**0.800**	**G2.1**	**0.800**	0.200	**0.800**	--	--	0.546	0.454
Stillbirne	14-003-1102	Koppigen	97	**G2**	**0.800**	**G2.1**	**0.800**	0.200	**0.800**	--	--	0.546	0.454
Rotbärtler	14-003-1248	Koppigen	97	**G2**	**0.800**	**G2.1**	**0.800**	0.200	**0.800**	--	--	0.546	0.454
Walliser Sämling	14-003-1580	Roggwil Hofen	98	**G2**	0.698	**G2.2**	0.698	0.302	0.698	--	--	0.329	0.671
Poire Honig Birnen	14-003-1540	Pierre-à-bot	98	**G2**	0.698	**G2.2**	0.698	0.302	0.698	--	--	0.329	0.671
Honigbirne	14-003-1540	Büron	98	**G2**	0.698	**G2.2**	0.698	0.302	0.698	--	--	0.329	0.671
Poire rougin	14-003-2525	Aubonne	99	**G1**	**0.881**	**G1.1**	**0.881**	**0.881**	0.119	0.546	0.454	--	--
Poire-Bouteille	14-003-1143	Aclens	99	**G1**	**0.881**	**G1.1**	**0.881**	**0.881**	0.119	0.546	0.454	--	--
Poire d' Orient	14-003-441	Pierre-à-bot	99	**G1**	**0.881**	**G1.1**	**0.881**	**0.881**	0.119	0.546	0.454	--	--
unknown	14-003-1342	Baden	100	**G1**	0.641	**G1.2**	0.761	0.641	0.359	0.239	0.761	--	--
unknown	14-003-1475	Büron	100	**G1**	0.641	**G1.2**	0.761	0.641	0.359	0.239	0.761	--	--
Stuttgarter Birne	14-003-774	Büron	100	**G1**	0.641	**G1.2**	0.761	0.641	0.359	0.239	0.761	--	--
Schürbirne	14-003-560	Bözberg-Vierlinden	100	**G1**	0.641	**G1.2**	0.761	0.641	0.359	0.239	0.761	--	--
Cuisse Dame	14-003-1559	Aclens	101	**G1**	0.762	**G1.2**	**0.957**	0.762	0.238	0.043	**0.957**	--	--
unknown	14-003-1043	Büron	101	**G1**	0.762	**G1.2**	**0.957**	0.762	0.238	0.043	**0.957**	--	--
Dornbirne	14-003-1523	Aclens	102	**G2**	**0.929**	**G2.1**	**0.929**	0.071	**0.929**	--	--	0.700	0.300
Dornbirne	14-003-1033	Baden	102	**G2**	**0.929**	**G2.1**	**0.929**	0.071	**0.929**	--	--	0.700	0.300
Rotbärtler	14-003-1404	Baden	103	**G2**	**0.969**	**G2.2**	**0.969**	0.031	**0.969**	--	--	0.073	**0.927**
Goldbirrli	14-003-984	Büron	103	**G2**	**0.969**	**G2.2**	**0.969**	0.031	**0.969**	--	--	0.073	**0.927**
Poire Muscat	14-003-423	Aclens	104	**G2**	**0.920**	**G2.2**	**0.920**	0.080	**0.920**	--	--	0.460	0.540
Poire Sept-en-Gueule	14-003-1134	Pierre-à-bot	104	**G2**	**0.920**	**G2.2**	**0.920**	0.080	**0.920**	--	--	0.460	0.540
Züribirne	14-003-796	Baden	105	**G2**	**0.954**	**G2.2**	**0.954**	0.046	**0.954**	--	--	0.389	0.611
Züribirne	14-003-797	Büron	105	**G2**	**0.954**	**G2.2**	**0.954**	0.046	**0.954**	--	--	0.389	0.611
Zuckerbirne	14-003-836	Büron	105	**G2**	**0.954**	**G2.2**	**0.954**	0.046	**0.954**	--	--	0.389	0.611
Köstliche von Charneu	14-003-1024	Baden	106	**G1**	**0.940**	**G1.2**	**0.940**	**0.940**	0.060	0.449	0.551	--	--
Köstliche von Charneu	14-003-344	Knonau	106	**G1**	**0.940**	**G1.2**	**0.940**	**0.940**	0.060	0.449	0.551	--	--
Meerbirne	14-003-1385	Baden	107	**G2**	0.585	**G2.1**	0.589	0.415	0.585	--	--	0.589	0.411

Meerbirne	14-003-706	Baden	107	**G2**	0.585	**G2.1**	0.589	0.415	0.585	--	--	0.589	0.411
Späte Islerbirne	14-003-1443	Baden	107	**G2**	0.585	**G2.1**	0.589	0.415	0.585	--	--	0.589	0.411
Wegbirne	14-003-1346	Koppigen	108	**G1**	**0.970**	**G1.2**	**0.970**	**0.970**	0.030	0.097	**0.903**	--	--
Clarmont	14-003-2504	Aclens	108	**G1**	**0.970**	**G1.2**	**0.970**	**0.970**	0.030	0.097	**0.903**	--	--
unknown	14-003-1079	Baden	108	**G1**	**0.970**	**G1.2**	**0.970**	**0.970**	0.030	0.097	**0.903**	--	--
Eierbirne	14-003-791	Büron	108	**G1**	**0.970**	**G1.2**	**0.970**	**0.970**	0.030	0.097	**0.903**	--	--
Winterbirne	14-003-877	Büron	108	**G1**	**0.970**	**G1.2**	**0.970**	**0.970**	0.030	0.097	**0.903**	--	--
Fressbirne	14-003-744	Büron	108	**G1**	**0.970**	**G1.2**	**0.970**	**0.970**	0.030	0.097	**0.903**	--	--
Märxlera	14-003-772	Büron	108	**G1**	**0.970**	**G1.2**	**0.970**	**0.970**	0.030	0.097	**0.903**	--	--
Pero Campestro	14-003-2564	Manno	109	**G1**	**0.963**	**G1.1**	**0.963**	**0.963**	0.037	0.632	0.368	--	--
Sal22	14-003-2552	Manno	109	**G1**	**0.963**	**G1.1**	**0.963**	**0.963**	0.037	0.632	0.368	--	--
Sconosciuto prob. locale	14-003-2590	Manno	110	**G1**	**0.975**	**G1.1**	**0.975**	**0.975**	0.025	**0.910**	0.090	--	--
Poire Giffard Bttb.	14-003-2537	Pierre-à-bot	110	**G1**	**0.975**	**G1.1**	**0.975**	**0.975**	0.025	**0.910**	0.090	--	--
Frühbirne	14-003-779	Baden	110	**G1**	**0.975**	**G1.1**	**0.975**	**0.975**	0.025	**0.910**	0.090	--	--
Butterbirne	14-003-986	Büron	110	**G1**	**0.975**	**G1.1**	**0.975**	**0.975**	0.025	**0.910**	0.090	--	--
Giffards Butterbirne	14-003-52	Büron	110	**G1**	**0.975**	**G1.1**	**0.975**	**0.975**	0.025	**0.910**	0.090	--	--
Legibirne	14-003-366	Höri	110	**G1**	**0.975**	**G1.1**	**0.975**	**0.975**	0.025	**0.910**	0.090	--	--
Pero peduncolo lungo	14-003-2560	Manno	111	**G1**	**0.973**	**G1.2**	**0.973**	**0.973**	0.027	0.064	**0.936**	--	--
Sconosciuto prob. locale	14-003-2583	Manno	111	**G1**	**0.973**	**G1.2**	**0.973**	**0.973**	0.027	0.064	**0.936**	--	--
unknown	14-003-1437	Baden	111	**G1**	**0.973**	**G1.2**	**0.973**	**0.973**	0.027	0.064	**0.936**	--	--
Butterbirne	14-003-785	Baden	111	**G1**	**0.973**	**G1.2**	**0.973**	**0.973**	0.027	0.064	**0.936**	--	--
Wulliwusch	14-003-817	Baden	111	**G1**	**0.973**	**G1.2**	**0.973**	**0.973**	0.027	0.064	**0.936**	--	--
unknown	14-003-758	Büron	111	**G1**	**0.973**	**G1.2**	**0.973**	**0.973**	0.027	0.064	**0.936**	--	--
Welsche Isler	14-003-1411	Büron	111	**G1**	**0.973**	**G1.2**	**0.973**	**0.973**	0.027	0.064	**0.936**	--	--
Ankenbirne	14-003-1309	Dürrenäsch	111	**G1**	**0.973**	**G1.2**	**0.973**	**0.973**	0.027	0.064	**0.936**	--	--
Goldlänglerbirne	14-003-886	Roggwil Riedern	112	**G1**	**0.942**	**G1.2**	**0.964**	**0.942**	0.058	0.036	**0.964**	--	--
Goldlängelerbirne	14-003-886	Büron	112	**G1**	**0.942**	**G1.2**	**0.964**	**0.942**	0.058	0.036	**0.964**	--	--
Speckbirne	14-003-803	Büron	112	**G1**	**0.942**	**G1.2**	**0.964**	**0.942**	0.058	0.036	**0.964**	--	--
Pera del coco	14-003-1519	Roggwil Riedern	113	**G1**	**0.984**	**G1.1**	**0.984**	**0.984**	0.016	**0.899**	0.101	--	--
Rosalettibirne/Frühe aus Trévoux	14-003-1059	Baden	113	**G1**	**0.984**	**G1.1**	**0.984**	**0.984**	0.016	**0.899**	0.101	--	--
Noir	14-003-1572	Roggwil Riedern	114	**G2**	**0.946**	**G2.2**	**0.946**	0.054	**0.946**	--	--	0.316	0.684
Isler	14-003-726	Roggwil Riedern	114	**G2**	**0.946**	**G2.2**	**0.946**	0.054	**0.946**	--	--	0.316	0.684
Madame Favre	14-003-380	Roggwil Riedern	115	**G1**	**0.921**	**G1.2**	**0.921**	**0.921**	0.079	0.354	0.646	--	--
Poire citron	14-003-1507	Aclens	115	**G1**	**0.921**	**G1.2**	**0.921**	**0.921**	0.079	0.354	0.646	--	--
Poire-citron	14-003-1534	Aclens	115	**G1**	**0.921**	**G1.2**	**0.921**	**0.921**	0.079	0.354	0.646	--	--
Poire Beurré d'Amanlis	14-003-1189	Pierre-à-bot	115	**G1**	**0.921**	**G1.2**	**0.921**	**0.921**	0.079	0.354	0.646	--	--
Winterkönigin	14-003-1023	Roggwil Riedern	116	**G1**	**0.959**	**G1.1**	**0.981**	**0.959**	0.041	**0.981**	0.019	--	--
Alexandrine Douillard	14-003-8	Roggwil Riedern	116	**G1**	**0.959**	**G1.1**	**0.981**	**0.959**	0.041	**0.981**	0.019	--	--
unknown	14-003-1491	Büron	116	**G1**	**0.959**	**G1.1**	**0.981**	**0.959**	0.041	**0.981**	0.019	--	--
Flons Dechantsbirne	14-003-1419	Roggwil Riedern	117	**G1**	**0.816**	**G1.1**	**0.893**	**0.816**	0.184	**0.893**	0.107	--	--
Poire Bergamotte	14-003-2529	Aclens	117	**G1**	**0.816**	**G1.1**	**0.893**	**0.816**	0.184	**0.893**	0.107	--	--
Poire de Zir	14-003-687	Pierre-à-bot	117	**G1**	**0.816**	**G1.1**	**0.893**	**0.816**	0.184	**0.893**	0.107	--	--
Poire Crassane	14-003-148	Pierre-à-bot	117	**G1**	**0.816**	**G1.1**	**0.893**	**0.816**	0.184	**0.893**	0.107	--	--
Grünbirne	14-003-750	Roggwil Riedern	118	**G2**	**0.879**	**G2.2**	**0.879**	0.121	**0.879**	--	--	0.344	0.656
Herteigler	14-003-802	Roggwil Riedern	118	**G2**	**0.879**	**G2.2**	**0.879**	0.121	**0.879**	--	--	0.344	0.656
Gelbmöstler	14-003-235	Höri	118	**G2**	**0.879**	**G2.2**	**0.879**	0.121	**0.879**	--	--	0.344	0.656
Buchsibire	14-003-1232	Koppigen	118	**G2**	**0.879**	**G2.2**	**0.879**	0.121	**0.879**	--	--	0.344	0.656
unknown	14-003-1442	Koppigen	118	**G2**	**0.879**	**G2.2**	**0.879**	0.121	**0.879**	--	--	0.344	0.656
Poire Collet	14-003-132	Pierre-à-bot	118	**G2**	**0.879**	**G2.2**	**0.879**	0.121	**0.879**	--	--	0.344	0.656
Kugelförmige Mostbirne	14-003-1398	Büron	118	**G2**	**0.879**	**G2.2**	**0.879**	0.121	**0.879**	--	--	0.344	0.656
Gelbsüsslerbirne	14-003-841	Büron	118	**G2**	**0.879**	**G2.2**	**0.879**	0.121	**0.879**	--	--	0.344	0.656
Büschelibirne	14-003-1327	Koppigen	119	**G1**	0.613	**G1.1**	0.613	0.613	0.387	0.514	0.486	--	--
Hirschbirli	14-003-1288	Koppigen	119	**G1**	0.613	**G1.1**	0.613	0.613	0.387	0.514	0.486	--	--
Einsiedlerbirne	14-003-1401	Baden	120	**G2**	**0.818**	**G2.2**	**0.878**	0.182	**0.818**	--	--	0.122	**0.878**
Lederbirne	14-003-961	Büron	120	**G2**	**0.818**	**G2.2**	**0.878**	0.182	**0.818**	--	--	0.122	**0.878**
Späte Weinbirne	14-003-977	Büron	121	**G1**	0.502	**G1.2**	**0.876**	0.502	0.498	0.124	**0.876**	--	--
Kellers Mostbirne	14-003-944	Büron	121	**G1**	0.502	**G1.2**	**0.876**	0.502	0.498	0.124	**0.876**	--	--
Couenla	14-003-2507	Aubonne	122	**G1**	0.539	**G1.2**	**0.942**	0.539	0.461	0.058	**0.942**	--	--
Poire Couenla	14-003-144	Aclens	122	**G1**	0.539	**G1.2**	**0.942**	0.539	0.461	0.058	**0.942**	--	--
Poire Couëla	14-003-143	Pierre-à-bot	122	**G1**	0.539	**G1.2**	**0.942**	0.539	0.461	0.058	**0.942**	--	--
Botzi jaune	14-003-2501	Aubonne	123	**G1**	0.796	**G1.2**	0.796	0.796	0.204	0.362	0.638	--	--
Botzi rouge	14-003-2502	Aubonne	123	**G1**	0.796	**G1.2**	0.796	0.796	0.204	0.362	0.638	--	--
Grüne Büschelibirne	14-003-270	Aclens	123	**G1**	0.796	**G1.2**	0.796	0.796	0.204	0.362	0.638	--	--
Poire à Botsi	14-003-1142	Aclens	123	**G1**	0.796	**G1.2**	0.796	0.796	0.204	0.362	0.638	--	--
Böztris	14-003-1564	Aclens	123	**G1**	0.796	**G1.2**	0.796	0.796	0.204	0.362	0.638	--	--
Poire Epine	14-003-1144	Aclens	123	**G1**	0.796	**G1.2**	0.796	0.796	0.204	0.362	0.638	--	--
Poire Verde	14-003-1530	Aclens	123	**G1**	0.796	**G1.2**	0.796	0.796	0.204	0.362	0.638	--	--
Poire à Botsi	14-003-1118	Pierre-à-bot	123	**G1**	0.796	**G1.2**	0.796	0.796	0.204	0.362	0.638	--	--
Hornuser	14-003-715	Baden	124	**G1**	**0.871**	**G1.1**	**0.871**	**0.871**	0.129	**0.843**	0.157	--	--
Fritzenbirne	14-003-1405	Büron	124	**G1**	**0.871**	**G1.1**	**0.871**	**0.871**	0.129	**0.843**	0.157	--	--
Schifferbirne	14-003-865	Büron	124	**G1**	**0.871**	**G1.1**	**0.871**	**0.871**	0.129	**0.843**	0.157	--	--
Arthur	14-003-819	Büron	124	**G1**	**0.871**	**G1.1**	**0.871**	**0.871**	0.129	**0.843**	0.157	--	--
Biesson	14-003-1547	Aclens	125	**G2**	0.586	**G2.2**	0.756	0.414	0.586	--	--	0.244	0.756
Poire Bièchon	14-003-57	Pierre-à-bot	125	**G2**	0.586	**G2.2**	0.756	0.414	0.586	--	--	0.244	0.756
Poire Biesson	14-003-1138	Pierre-à-bot	125	**G2**	0.586	**G2.2**	0.756	0.414	0.586	--	--	0.244	0.756
Poire Pomme-Poire	14-003-472	Pierre-à-bot	125	**G2**	0.586	**G2.2**	0.756	0.414	0.586	--	--	0.244	0.756
Holzbirne	14-003-1517	Aclens	126	**G1**	**0.843**	**G1.2**	**0.843**	**0.843**	0.157	0.233	0.767	--	--
Winterchristbirne	14-003-675	Baden	126	**G1**	**0.843**	**G1.2**	**0.843**	**0.843**	0.157	0.233	0.767	--	--
Winter-Christbirne	14-003-1272	Büron	126	**G1**	**0.843**	**G1.2**	**0.843**	**0.843**	0.157	0.233	0.767	--	--
Sal109	14-003-2578	Manno	127	**G1**	**0.948**	**G1.1**	**0.948**	**0.948**	0.052	**0.881**	0.119	--	--
Triacca	14-003-2516	Aubonne	127	**G1**	**0.948**	**G1.1**	**0.948**	**0.948**	0.052	**0.881**	0.119	--	--
Sal71	14-003-2554	Manno	128	**G1**	**0.966**	**G1.1**	**0.971**	**0.966**	0.034	**0.971**	0.029	--	--
unknown	14-003-1453	Büron	128	**G1**	**0.966**	**G1.1**	**0.971**	**0.966**	0.034	**0.971**	0.029	--	--
Williams' Bon Chretien	Reference 8	Uni Reading	128	**G1**	**0.966**	**G1.1**	**0.971**	**0.966**	0.034	**0.971**	0.029	--	--
Pero Canonica	14-003-2566	Manno	129	**G1**	**0.973**	**G1.1**	**0.973**	**0.973**	0.027	**0.922**	0.078	--	--
Pero Canonica 2	14-003-2708	Manno	129	**G1**	**0.973**	**G1.1**	**0.973**	**0.973**	0.027	**0.922**	0.078	--	--
Beurrée grise	14-003-1565	Aclens	129	**G1**	**0.973**	**G1.1**	**0.973**	**0.973**	0.027	**0.922**	0.078	--	--

Deux soeurs	14-003-1527	Büron	129	G1	0.973	G1.1	0.973	0.973	0.027	0.922	0.078	--	--
Doppelte Phillipsbirne	14-003-167	Knonau	129	G1	0.973	G1.1	0.973	0.973	0.027	0.922	0.078	--	--
Pero "chiappona"	14-003-2704	Manno	130	G2	0.581	G2.1	0.581	0.419	0.581	--	--	0.567	0.433
Poire de fer	14-003-1531	Aclens	130	G2	0.581	G2.1	0.581	0.419	0.581	--	--	0.567	0.433
Poire Loup	14-003-372	Aclens	130	G2	0.581	G2.1	0.581	0.419	0.581	--	--	0.567	0.433
Poire à viande	14-003-1539	Aclens	130	G2	0.581	G2.1	0.581	0.419	0.581	--	--	0.567	0.433
Poire de Livre?	14-003-1160	Aclens	130	G2	0.581	G2.1	0.581	0.419	0.581	--	--	0.567	0.433
Poire-Livre	14-003-369	Pierre-à-bot	130	G2	0.581	G2.1	0.581	0.419	0.581	--	--	0.567	0.433
Poire Loup	14-003-2545	Pierre-à-bot	130	G2	0.581	G2.1	0.581	0.419	0.581	--	--	0.567	0.433
Poire à Viande	14-003-645	Pierre-à-bot	130	G2	0.581	G2.1	0.581	0.419	0.581	--	--	0.567	0.433
Sürler	14-003-731	Roggwil Riedern	131	G2	0.977	G2.2	0.977	0.023	0.977	--	--	0.273	0.727
Guntershauser	14-003-790	Roggwil Riedern	131	G2	0.977	G2.2	0.977	0.023	0.977	--	--	0.273	0.727
unknown	14-003-809	Roggwil Riedern	131	G2	0.977	G2.2	0.977	0.023	0.977	--	--	0.273	0.727
Imbler	14-003-1427	Büron	131	G2	0.977	G2.2	0.977	0.023	0.977	--	--	0.273	0.727
Teigbirne	14-003-1070	Büron	131	G2	0.977	G2.2	0.977	0.023	0.977	--	--	0.273	0.727
Teigbirne	14-003-1038	Roggwil Riedern	132	G2	0.909	G2.2	0.909	0.091	0.909	--	--	0.308	0.692
Tiroler	14-003-801	Roggwil Riedern	132	G2	0.909	G2.2	0.909	0.091	0.909	--	--	0.308	0.692
Fleischbirne	14-003-827	Roggwil Riedern	132	G2	0.909	G2.2	0.909	0.091	0.909	--	--	0.308	0.692
Welschbärtler	14-003-878	Roggwil Riedern	132	G2	0.909	G2.2	0.909	0.091	0.909	--	--	0.308	0.692
Spiesslerbirne	14-003-716	Roggwil Riedern	132	G2	0.909	G2.2	0.909	0.091	0.909	--	--	0.308	0.692
Fleischbirne	14-003-904	Roggwil Riedern	132	G2	0.909	G2.2	0.909	0.091	0.909	--	--	0.308	0.692
Bärikerbirne	14-003-27	Höri	132	G2	0.909	G2.2	0.909	0.091	0.909	--	--	0.308	0.692
Kleine Wasserbirne	14-003-1334	Koppigen	132	G2	0.909	G2.2	0.909	0.091	0.909	--	--	0.308	0.692
unknown	14-003-1092	Koppigen	132	G2	0.909	G2.2	0.909	0.091	0.909	--	--	0.308	0.692
Bärikerbirne	14-003-27	Roggwil Hofen	132	G2	0.909	G2.2	0.909	0.091	0.909	--	--	0.308	0.692
Bärikerbirne	14-003-27	Aubonne	132	G2	0.909	G2.2	0.909	0.091	0.909	--	--	0.308	0.692
Zwiebelbirne	14-003-698	Pierre-à-bot	132	G2	0.909	G2.2	0.909	0.091	0.909	--	--	0.308	0.692
Züribirne	14-003-1396	Baden	132	G2	0.909	G2.2	0.909	0.091	0.909	--	--	0.308	0.692
Züribirne Typ 2	14-003-696	Baden	132	G2	0.909	G2.2	0.909	0.091	0.909	--	--	0.308	0.692
Vater	14-003-792	Büron	132	G2	0.909	G2.2	0.909	0.091	0.909	--	--	0.308	0.692
Tirolerbirne	14-003-958	Büron	132	G2	0.909	G2.2	0.909	0.091	0.909	--	--	0.308	0.692
Späckbire	14-003-1039	Büron	132	G2	0.909	G2.2	0.909	0.091	0.909	--	--	0.308	0.692
Fleischbirne	14-003-904	Büron	132	G2	0.909	G2.2	0.909	0.091	0.909	--	--	0.308	0.692
Tiroler	14-003-963	Büron	132	G2	0.909	G2.2	0.909	0.091	0.909	--	--	0.308	0.692
Niederländer Birnen	14-003-1423	Roggwil Riedern	133	G2	0.960	G2.1	0.960	0.040	0.960	--	--	0.957	0.043
Landsknechtler	14-003-352	Roggwil Riedern	133	G2	0.960	G2.1	0.960	0.040	0.960	--	--	0.957	0.043
Glöggler	14-003-804	Roggwil Riedern	133	G2	0.960	G2.1	0.960	0.040	0.960	--	--	0.957	0.043
Fleischbirne	14-003-828	Roggwil Riedern	133	G2	0.960	G2.1	0.960	0.040	0.960	--	--	0.957	0.043
Landsknechtler	14-003-888	Roggwil Hofen	133	G2	0.960	G2.1	0.960	0.040	0.960	--	--	0.957	0.043
Längler	14-003-3669	Roggwil Hofen	133	G2	0.960	G2.1	0.960	0.040	0.960	--	--	0.957	0.043
Rotlängler Dornbirne	14-003-526	Baden	133	G2	0.960	G2.1	0.960	0.040	0.960	--	--	0.957	0.043
Rotlängler	14-003-1421	Baden	133	G2	0.960	G2.1	0.960	0.040	0.960	--	--	0.957	0.043
Landsknechtler	14-003-888	Büron	133	G2	0.960	G2.1	0.960	0.040	0.960	--	--	0.957	0.043
Dörrbirne	14-003-824	Büron	133	G2	0.960	G2.1	0.960	0.040	0.960	--	--	0.957	0.043
Kilbibirnen	14-003-755	Roggwil Riedern	134	G2	0.979	G2.2	0.979	0.021	0.979	--	--	0.382	0.618
Schutzibirne	14-003-561	Roggwil Hofen	134	G2	0.979	G2.2	0.979	0.021	0.979	--	--	0.382	0.618
Welschbirne	14-003-924	Büron	134	G2	0.979	G2.2	0.979	0.021	0.979	--	--	0.382	0.618
Strohbirrli	14-003-981	Büron	134	G2	0.979	G2.2	0.979	0.021	0.979	--	--	0.382	0.618
Fuchsbirne	14-003-842	Büron	134	G2	0.979	G2.2	0.979	0.021	0.979	--	--	0.382	0.618
Feigenbirne	14-003-882	Büron	134	G2	0.979	G2.2	0.979	0.021	0.979	--	--	0.382	0.618
Luzerner Weissbirne	14-003-789	Roggwil Riedern	135	G2	0.556	G2.1	0.556	0.444	0.556	--	--	0.534	0.466
Glockenbirne	14-003-1014	Roggwil Riedern	135	G2	0.556	G2.1	0.556	0.444	0.556	--	--	0.534	0.466
Teigbirne	14-003-526	Roggwil Riedern	136	G1	0.951	G1.2	0.951	0.951	0.049	0.052	0.948	--	--
Amanlis Butterbirne	14-003-720	Roggwil Riedern	136	G1	0.951	G1.2	0.951	0.951	0.049	0.052	0.948	--	--
Chriesibirne	14-003-875	Roggwil Riedern	137	G2	0.696	G2.1	0.696	0.304	0.696	--	--	0.505	0.495
Langstieler	14-003-356	Roggwil Hofen	137	G2	0.696	G2.1	0.696	0.304	0.696	--	--	0.505	0.495
Krummstieler	14-003-345	Aubonne	137	G2	0.696	G2.1	0.696	0.304	0.696	--	--	0.505	0.495
Chriesibirne	14-003-921	Büron	137	G2	0.696	G2.1	0.696	0.304	0.696	--	--	0.505	0.495
Appenzeller Langstieler	14-003-19	privat	137	G2	0.696	G2.1	0.696	0.304	0.696	--	--	0.505	0.495
Appenzeller Langstieler	14-003-19	privat	137	G2	0.696	G2.1	0.696	0.304	0.696	--	--	0.505	0.495
Juxbirne	14-003-909	Büron	138	G1	0.826	G1.2	0.970	0.826	0.174	0.030	0.970	--	--
unknown	14-003-1473	Büron	138	G1	0.826	G1.2	0.970	0.826	0.174	0.030	0.970	--	--
Zitronenbirne	14-003-688	Roggwil Hofen	139	G1	0.895	G1.2	0.970	0.895	0.105	0.030	0.970	--	--
Oberösterreicher Weinbirne	14-003-434	Höri	139	G1	0.895	G1.2	0.970	0.895	0.105	0.030	0.970	--	--
Amisberger	14-003-1374	Baden	140	G2	0.806	G2.1	0.806	0.194	0.806	--	--	0.513	0.487
Tropfbirne	14-003-956	Büron	140	G2	0.806	G2.1	0.806	0.194	0.806	--	--	0.513	0.487
Grünmöstler	14-003-273	Roggwil Hofen	141	G2	0.889	G2.2	0.889	0.111	0.889	--	--	0.367	0.633
Weinbirne	14-003-1057	Baden	141	G2	0.889	G2.2	0.889	0.111	0.889	--	--	0.367	0.633
Wettinger Holzbirne	14-003-668	Baden	141	G2	0.889	G2.2	0.889	0.111	0.889	--	--	0.367	0.633
Züribieter	14-003-1001	Büron	141	G2	0.889	G2.2	0.889	0.111	0.889	--	--	0.367	0.633
Fricktaler	14-003-1382	Büron	141	G2	0.889	G2.2	0.889	0.111	0.889	--	--	0.367	0.633
Heulampen grosser Typ	14-003-1581	Roggwil Hofen	142	G2	0.966	G2.1	0.966	0.034	0.966	--	--	0.850	0.150
Heilemben	14-003-951	Büron	142	G2	0.966	G2.1	0.966	0.034	0.966	--	--	0.850	0.150
Poire Plat	14-003-469	Aubonne	143	G1	0.958	G1.2	0.958	0.958	0.042	0.075	0.925	--	--
Poire Pipe	14-003-467	Pierre-à-bot	143	G1	0.958	G1.2	0.958	0.958	0.042	0.075	0.925	--	--
Poire Plat	14-003-469	Pierre-à-bot	143	G1	0.958	G1.2	0.958	0.958	0.042	0.075	0.925	--	--
Bratbirne von Versoix	14-003-78	Aubonne	144	G1	0.755	G1.2	0.978	0.755	0.245	0.022	0.978	--	--
Saint-Laurent	14-003-1573	Aclens	144	G1	0.755	G1.2	0.978	0.755	0.245	0.022	0.978	--	--
Poire Saint-Laurent II	14-003-1158	Aclens	144	G1	0.755	G1.2	0.978	0.755	0.245	0.022	0.978	--	--
St-Laurent	14-003-1566	Aclens	144	G1	0.755	G1.2	0.978	0.755	0.245	0.022	0.978	--	--
Poire Blèque	14-003-62	Pierre-à-bot	144	G1	0.755	G1.2	0.978	0.755	0.245	0.022	0.978	--	--
Poire Channe	14-003-112	Pierre-à-bot	144	G1	0.755	G1.2	0.978	0.755	0.245	0.022	0.978	--	--
Poire Saint-Laurent	14-003-543	Pierre-à-bot	144	G1	0.755	G1.2	0.978	0.755	0.245	0.022	0.978	--	--
Löhninger Mehlbirne	14-003-370	Pierre-à-bot	145	G2	0.966	G2.1	0.966	0.034	0.966	--	--	0.628	0.372
Falsche Luise	14-003-1363	Büron	145	G2	0.966	G2.1	0.966	0.034	0.966	--	--	0.628	0.372
Poire San-Règle	14-003-548	Pierre-à-bot	146	G1	0.608	G1.2	0.910	0.608	0.392	0.090	0.910	--	--
Poire Surègle	14-003-613	Pierre-à-bot	146	G1	0.608	G1.2	0.910	0.608	0.392	0.090	0.910	--	--

Weihnachtsbirne / Hardenpont	14-003-1036	Baden	147	**G1**	**0.975**	**G1.1**	**0.975**	**0.975**	0.025	**0.973**	0.027	--	--
Rietwiser	14-003-1073	Baden	147	**G1**	**0.975**	**G1.1**	**0.975**	**0.975**	0.025	**0.973**	0.027	--	--
Boncristián	14-003-2587	Manno	148	**G2**	0.702	**G2.1**	0.702	0.298	0.702	--	--	0.579	0.421
Boncristián	14-003-2587	Manno	148	**G2**	0.702	**G2.1**	0.702	0.298	0.702	--	--	0.579	0.421
Bon Cristian	14-003-1520	Büron	148	**G2**	0.702	**G2.1**	0.702	0.298	0.702	--	--	0.579	0.421
Wasserbirne	14-003-653	Nuclear stock	149	**G2**	**0.802**	**G2.1**	**0.802**	0.198	**0.802**	--	--	0.547	0.453
Erdhauser	14-003-193	Roggwil Hofen	149	**G2**	**0.802**	**G2.1**	**0.802**	0.198	**0.802**	--	--	0.547	0.453
Grosse Wasserbirne von Chailly	14-003-264	Aubonne	149	**G2**	**0.802**	**G2.1**	**0.802**	0.198	**0.802**	--	--	0.547	0.453
Wettingerbirne	14-003-2519	Aubonne	149	**G2**	**0.802**	**G2.1**	**0.802**	0.198	**0.802**	--	--	0.547	0.453
Poire Maude	14-003-397	Pierre-à-bot	149	**G2**	**0.802**	**G2.1**	**0.802**	0.198	**0.802**	--	--	0.547	0.453
Poire Rouge	14-003-1132	Pierre-à-bot	149	**G2**	**0.802**	**G2.1**	**0.802**	0.198	**0.802**	--	--	0.547	0.453
Wurlitzer	14-003-1015	Baden	149	**G2**	**0.802**	**G2.1**	**0.802**	0.198	**0.802**	--	--	0.547	0.453
Portamenter	14-003-473	Baden	149	**G2**	**0.802**	**G2.1**	**0.802**	0.198	**0.802**	--	--	0.547	0.453
Beihäusler	14-003-1394	Büron	149	**G2**	**0.802**	**G2.1**	**0.802**	0.198	**0.802**	--	--	0.547	0.453
unknown	14-003-1415	Büron	149	**G2**	**0.802**	**G2.1**	**0.802**	0.198	**0.802**	--	--	0.547	0.453
Butterbirne	14-003-901	Büron	149	**G2**	**0.802**	**G2.1**	**0.802**	0.198	**0.802**	--	--	0.547	0.453
Vifisbirne	14-003-210	Dürrenäsch	149	**G2**	**0.802**	**G2.1**	**0.802**	0.198	**0.802**	--	--	0.547	0.453
Eisenbirne	14-003-1063	Roggwil Riedern	150	**G2**	**0.853**	**G2.2**	**0.853**	0.147	**0.853**	--	--	0.419	0.581
Späte Weinbirne	14-003-586	Roggwil Hofen	150	**G2**	**0.853**	**G2.2**	**0.853**	0.147	**0.853**	--	--	0.419	0.581
Verte de Dully	14-003-2517	Aclens	150	**G2**	**0.853**	**G2.2**	**0.853**	0.147	**0.853**	--	--	0.419	0.581
unknown	14-003-1494	Büron	150	**G2**	**0.853**	**G2.2**	**0.853**	0.147	**0.853**	--	--	0.419	0.581
unbekannte Mostbirne	14-003-946	Büron	150	**G2**	**0.853**	**G2.2**	**0.853**	0.147	**0.853**	--	--	0.419	0.581
Gunteschhuser	14-003-880	Büron	150	**G2**	**0.853**	**G2.2**	**0.853**	0.147	**0.853**	--	--	0.419	0.581
unknown	14-003-1454	Büron	151	**G1**	**0.956**	**G1.1**	**0.956**	**0.956**	0.044	**0.857**	0.143	--	--
Heubirne	14-003-815	Büron	151	**G1**	**0.956**	**G1.1**	**0.956**	**0.956**	0.044	**0.857**	0.143	--	--
Heubirne	14-003-784	Büron	151	**G1**	**0.956**	**G1.1**	**0.956**	**0.956**	0.044	**0.857**	0.143	--	--
Hingeli	14-003-539	Baden	152	**G2**	0.505	**G2.2**	0.798	0.495	0.505	--	--	0.202	0.798
Studenweider	14-003-983	Büron	152	**G2**	0.505	**G2.2**	0.798	0.495	0.505	--	--	0.202	0.798
Hasenbirne	14-003-3671	Roggwil Hofen	153	**G2**	**0.981**	**G2.2**	**0.981**	0.019	**0.981**	--	--	0.067	**0.933**
Chriesibirne	14-003-794	Büron	153	**G2**	**0.981**	**G2.2**	**0.981**	0.019	**0.981**	--	--	0.067	**0.933**
Pero grande destra	14-003-2699	Manno	154	**G1**	**0.977**	**G1.1**	**0.978**	**0.977**	0.023	**0.978**	0.022	--	--
Fedant	14-003-1383	Roggwil Riedern	154	**G1**	**0.977**	**G1.1**	**0.978**	**0.977**	0.023	**0.978**	0.022	--	--
Saure Theilersbirne / Riedwiser ?	14-003-1022	Roggwil Riedern	155	**G2**	**0.982**	**G2.2**	**0.982**	0.018	**0.982**	--	--	0.081	**0.919**
unknown	14-003-1086	Baden	155	**G2**	**0.982**	**G2.2**	**0.982**	0.018	**0.982**	--	--	0.081	**0.919**
Zuckerbirne	14-003-1388	Roggwil Riedern	156	**G2**	**0.971**	**G2.2**	**0.971**	0.029	**0.971**	--	--	0.298	0.702
Speckbirne	14-003-907	Büron	156	**G2**	**0.971**	**G2.2**	**0.971**	0.029	**0.971**	--	--	0.298	0.702
Goldbirnen	14-003-733	Roggwil Riedern	157	**G2**	**0.923**	**G2.2**	**0.923**	0.077	**0.923**	--	--	0.127	**0.873**
Eierbirne	14-003-835	Roggwil Riedern	157	**G2**	**0.923**	**G2.2**	**0.923**	0.077	**0.923**	--	--	0.127	**0.873**
Stielbirne / Grosse Roggenbirne	14-003-1408	Roggwil Riedern	158	**G2**	**0.987**	**G2.2**	**0.987**	0.013	**0.987**	--	--	0.101	**0.899**
Freulerbirne	14-003-735	Roggwil Riedern	158	**G2**	**0.987**	**G2.2**	**0.987**	0.013	**0.987**	--	--	0.101	**0.899**
Poire de Moisson	14-003-1502	Aclens	158	**G2**	**0.987**	**G2.2**	**0.987**	0.013	**0.987**	--	--	0.101	**0.899**
Weinbirne	14-003-813	Roggwil Riedern	159	**G2**	**0.942**	**G2.1**	**0.942**	0.058	**0.942**	--	--	**0.809**	0.191
Chriesibirne	14-003-889	Büron	159	**G2**	**0.942**	**G2.1**	**0.942**	0.058	**0.942**	--	--	**0.809**	0.191
Petersbirne	14-003-2722	Roggwil Hofen	160	**G1**	**0.845**	**G1.2**	**0.860**	**0.845**	0.155	0.140	**0.860**	--	--
Augustbirne	14-003-282	Baden	160	**G1**	**0.845**	**G1.2**	**0.860**	**0.845**	0.155	0.140	**0.860**	--	--
Heubirne	14-003-760	Büron	160	**G1**	**0.845**	**G1.2**	**0.860**	**0.845**	0.155	0.140	**0.860**	--	--
Magdalenen	14-003-881	Büron	160	**G1**	**0.845**	**G1.2**	**0.860**	**0.845**	0.155	0.140	**0.860**	--	--
Herbstlängelen	14-003-761	Roggwil Riedern	161	**G2**	**0.981**	**G2.1**	**0.981**	0.019	**0.981**	--	--	**0.942**	0.058
Herbstlängler	14-003-768	Roggwil Riedern	161	**G2**	**0.981**	**G2.1**	**0.981**	0.019	**0.981**	--	--	**0.942**	0.058
Herbstlängler	14-003-299	Roggwil Hofen	161	**G2**	**0.981**	**G2.1**	**0.981**	0.019	**0.981**	--	--	**0.942**	0.058
General von Coligny	14-003-240	Aclens	161	**G2**	**0.981**	**G2.1**	**0.981**	0.019	**0.981**	--	--	**0.942**	0.058
Herbstlängeln	14-003-890	Büron	161	**G2**	**0.981**	**G2.1**	**0.981**	0.019	**0.981**	--	--	**0.942**	0.058
Grünbirne	14-003-823	Roggwil Riedern	162	**G2**	**0.945**	**G2.2**	**0.945**	0.055	**0.945**	--	--	0.250	0.750
Rotkeller	14-003-884	Büron	162	**G2**	**0.945**	**G2.2**	**0.945**	0.055	**0.945**	--	--	0.250	0.750
Flaschenbirne	14-003-826	Roggwil Riedern	163	**G2**	**0.954**	**G2.2**	**0.954**	0.046	**0.954**	--	--	0.326	0.674
Lederbirne	14-003-1353	Koppigen	163	**G2**	**0.954**	**G2.2**	**0.954**	0.046	**0.954**	--	--	0.326	0.674
Chrisibirne	14-003-782	Büron	163	**G2**	**0.954**	**G2.2**	**0.954**	0.046	**0.954**	--	--	0.326	0.674
Worglibiere	14-003-1278	Koppigen	164	**G2**	**0.963**	**G2.1**	**0.963**	0.037	**0.963**	--	--	**0.927**	0.073
Pfundbirne	14-003-1344	Koppigen	164	**G2**	**0.963**	**G2.1**	**0.963**	0.037	**0.963**	--	--	**0.927**	0.073
Heubirne	14-003-1266	Koppigen	165	**G2**	**0.829**	**G2.1**	**0.829**	0.171	**0.829**	--	--	**0.803**	0.197
Zuckerbirne / Hanslibirne	14-003-1093	Koppigen	165	**G2**	**0.829**	**G2.1**	**0.829**	0.171	**0.829**	--	--	**0.803**	0.197
Wörger	14-003-1227	Koppigen	166	**G2**	**0.851**	**G2.1**	**0.851**	0.149	**0.851**	--	--	**0.824**	0.176
Aermelbirne	14-003-1345	Koppigen	166	**G2**	**0.851**	**G2.1**	**0.851**	0.149	**0.851**	--	--	**0.824**	0.176
Längler Kannenbirne	14-003-1253	Koppigen	166	**G2**	**0.851**	**G2.1**	**0.851**	0.149	**0.851**	--	--	**0.824**	0.176
Entenbirne	14-003-1098	Koppigen	167	**G2**	**0.912**	**G2.1**	**0.912**	0.088	**0.912**	--	--	**0.909**	0.091
Sidebire	14-003-1290	Koppigen	167	**G2**	**0.912**	**G2.1**	**0.912**	0.088	**0.912**	--	--	**0.909**	0.091
unknown	14-003-1343	Koppigen	167	**G2**	**0.912**	**G2.1**	**0.912**	0.088	**0.912**	--	--	**0.909**	0.091
Coulliarde Etrangleuse	14-003-1280	Koppigen	168	**G2**	**0.972**	**G2.1**	**0.972**	0.028	**0.972**	--	--	0.599	0.401
Kannenbirne	14-003-1239	Büron	168	**G2**	**0.972**	**G2.1**	**0.972**	0.028	**0.972**	--	--	0.599	0.401
Butterbirne	14-003-1380	Baden	169	**G1**	**0.981**	**G1.1**	**0.981**	**0.981**	0.019	**0.958**	0.042	--	--
unknown	14-003-1441	Büron	169	**G1**	**0.981**	**G1.1**	**0.981**	**0.981**	0.019	**0.958**	0.042	--	--
Guntershauser	14-003-280	Roggwil Hofen	170	**G1**	**0.890**	**G1.2**	**0.969**	**0.890**	0.110	0.031	**0.969**	--	--
Poire Klettgauer Dornbirne	14-003-335	Pierre-à-bot	170	**G1**	**0.890**	**G1.2**	**0.969**	**0.890**	0.110	0.031	**0.969**	--	--
Schwarzrädler	14-003-563	Roggwil Hofen	171	**G2**	**0.960**	**G2.1**	**0.960**	0.040	**0.960**	--	--	0.526	0.474
Schwarzrädler	14-003-563	Büron	171	**G2**	**0.960**	**G2.1**	**0.960**	0.040	**0.960**	--	--	0.526	0.474
Julidechantsbirne	14-003-325	Roggwil Hofen	172	**G1**	**0.907**	**G1.1**	**0.947**	**0.907**	0.093	**0.947**	0.053	--	--
Winterdiole	14-003-1226	Baden	172	**G1**	**0.907**	**G1.1**	**0.947**	**0.907**	0.093	**0.947**	0.053	--	--
Heubirli	14-003-987	Büron	172	**G1**	**0.907**	**G1.1**	**0.947**	**0.907**	0.093	**0.947**	0.053	--	--
Julibirne	14-003-839	Büron	172	**G1**	**0.907**	**G1.1**	**0.947**	**0.907**	0.093	**0.947**	0.053	--	--
Happerswiler	14-003-289	Roggwil Hofen	173	**G2**	0.777	**G2.2**	0.777	0.223	0.777	--	--	0.302	0.698
Happerswiler	14-003-289	Baden	173	**G2**	0.777	**G2.2**	0.777	0.223	0.777	--	--	0.302	0.698
Weissbirne	14-003-781	Büron	173	**G2**	0.777	**G2.2**	0.777	0.223	0.777	--	--	0.302	0.698
Poire muscat Blessens	14-003-2522	Aubonne	174	**G2**	0.538	**G2.2**	0.538	0.462	0.538	--	--	0.488	0.512
Muscat	14-003-1524	Aclens	174	**G2**	0.538	**G2.2**	0.538	0.462	0.538	--	--	0.488	0.512
Poire Muscat	14-003-422	Aclens	174	**G2**	0.538	**G2.2**	0.538	0.462	0.538	--	--	0.488	0.512
Poire Mouchca	14-003-417	Pierre-à-bot	174	**G2**	0.538	**G2.2**	0.538	0.462	0.538	--	--	0.488	0.512
Poire Muscat	14-003-421	Pierre-à-bot	174	**G2**	0.538	**G2.2**	0.538	0.462	0.538	--	--	0.488	0.512

Gratsserbirne	14-003-1508	Aclens	175	**G1**	**0.968**	**G1.1**	**0.968**	**0.968**	0.032	0.736	0.264	--	--
Feigenbirne	14-003-1422	Büron	175	**G1**	**0.968**	**G1.1**	**0.968**	**0.968**	0.032	0.736	0.264	--	--
Holzerbirne	14-003-311	Knonau	175	**G1**	**0.968**	**G1.1**	**0.968**	**0.968**	0.032	0.736	0.264	--	--
unknown	14-003-1078	Baden	176	**G2**	**0.965**	**G2.1**	**0.965**	0.035	**0.965**	--	--	0.516	0.484
Reinhölzler	14-003-1390	Büron	176	**G2**	**0.965**	**G2.1**	**0.965**	0.035	**0.965**	--	--	0.516	0.484
Poire Cavouirou	14-003-105	Pierre-à-bot	177	**G1**	0.671	**G1.2**	**0.935**	0.671	0.329	0.065	**0.935**	--	--
Poire Gode	14-003-248	Pierre-à-bot	177	**G1**	0.671	**G1.2**	**0.935**	0.671	0.329	0.065	**0.935**	--	--
Poire Martignerin	14-003-394	Pierre-à-bot	177	**G1**	0.671	**G1.2**	**0.935**	0.671	0.329	0.065	**0.935**	--	--
Poire Recordon	14-003-494	Pierre-à-bot	177	**G1**	0.671	**G1.2**	**0.935**	0.671	0.329	0.065	**0.935**	--	--
Poire Verdan	14-003-639	Pierre-à-bot	177	**G1**	0.671	**G1.2**	**0.935**	0.671	0.329	0.065	**0.935**	--	--
Poire Verdet	14-003-640	Pierre-à-bot	177	**G1**	0.671	**G1.2**	**0.935**	0.671	0.329	0.065	**0.935**	--	--
Mailänder	14-003-1061	Baden	178	**G2**	0.738	**G2.1**	**0.880**	0.262	0.738	--	--	**0.880**	0.120
Deerbirne / Mehlbirne	14-003-1030	Baden	178	**G2**	0.738	**G2.1**	**0.880**	0.262	0.738	--	--	**0.880**	0.120
Poire Grummbirli	14-003-267	Pierre-à-bot	179	**G2**	0.674	**G2.2**	0.674	0.326	0.674	--	--	0.366	0.634
Poire à grande Queue	14-003-484	Pierre-à-bot	179	**G2**	0.674	**G2.2**	0.674	0.326	0.674	--	--	0.366	0.634
Stilbirne	14-003-1090	Büron	179	**G2**	0.674	**G2.2**	0.674	0.326	0.674	--	--	0.366	0.634
Poire Sept en gueule	14-003-571	Pierre-à-bot	180	**G2**	**0.855**	**G2.2**	**0.868**	0.145	**0.855**	--	--	0.132	**0.868**
Poire Sept-en-Gueule	14-003-1133	Pierre-à-bot	180	**G2**	**0.855**	**G2.2**	**0.868**	0.145	**0.855**	--	--	0.132	**0.868**
Thurgäuerli	14-003-808	Roggwil Riedern	181	**G2**	0.536	**G2.1**	0.536	0.464	0.536	--	--	0.507	0.493
Thurgauer Weinbirne	14-003-780	Roggwil Riedern	181	**G2**	0.536	**G2.1**	0.536	0.464	0.536	--	--	0.507	0.493
Thurgäuerli	14-003-856	Büron	181	**G2**	0.536	**G2.1**	0.536	0.464	0.536	--	--	0.507	0.493
unknown	14-003-1341	Koppigen	182	**G2**	**0.924**	**G2.2**	**0.924**	0.076	**0.924**	--	--	0.347	0.653
Kleine Süsse	14-003-1099	Koppigen	182	**G2**	**0.924**	**G2.2**	**0.924**	0.076	**0.924**	--	--	0.347	0.653
Poire Rondatte	14-003-513	Pierre-à-bot	183	**G2**	**0.825**	**G2.1**	**0.825**	0.175	**0.825**	--	--	0.528	0.472
späte Weinbirne	14-003-1513	Pierre-à-bot	183	**G2**	**0.825**	**G2.1**	**0.825**	0.175	**0.825**	--	--	0.528	0.472
Dornbirne	14-003-773	Büron	183	**G2**	**0.825**	**G2.1**	**0.825**	0.175	**0.825**	--	--	0.528	0.472
Winterchristbirne	14-003-1247	Roggwil Riedern	184	**G2**	**0.867**	**G2.2**	**0.867**	0.133	**0.867**	--	--	0.462	0.538
Mostbirne	14-003-1293	Koppigen	184	**G2**	**0.867**	**G2.2**	**0.867**	0.133	**0.867**	--	--	0.462	0.538
unknown	14-003-1336	Koppigen	184	**G2**	**0.867**	**G2.2**	**0.867**	0.133	**0.867**	--	--	0.462	0.538
unknown	14-003-1107	Koppigen	184	**G2**	**0.867**	**G2.2**	**0.867**	0.133	**0.867**	--	--	0.462	0.538
Bergamotte	14-003-1299	Koppigen	184	**G2**	**0.867**	**G2.2**	**0.867**	0.133	**0.867**	--	--	0.462	0.538
Vifisbirne	14-003-1269	Koppigen	184	**G2**	**0.867**	**G2.2**	**0.867**	0.133	**0.867**	--	--	0.462	0.538
Augustbirne	14-003-749	Büron	185	**G1**	**0.954**	**G1.1**	**0.954**	**0.954**	0.046	**0.843**	0.157	--	--
Heuerbirne	14-003-970	Büron	185	**G1**	**0.954**	**G1.1**	**0.954**	**0.954**	0.046	**0.843**	0.157	--	--
Bergamotte	14-003-954	Baden	186	**G2**	**0.967**	**G2.1**	**0.967**	0.033	**0.967**	--	--	0.594	0.406
Heubirne	14-003-831	Büron	186	**G2**	**0.967**	**G2.1**	**0.967**	0.033	**0.967**	--	--	0.594	0.406
Poire Verte Codia	14-003-1120	Pierre-à-bot	187	**G2**	0.695	**G2.1**	**0.828**	0.305	0.695	--	--	**0.828**	0.172
Johannisbirne	14-003-1021	Baden	187	**G2**	0.695	**G2.1**	**0.828**	0.305	0.695	--	--	**0.828**	0.172
Birne Olivia Stoll	-	privat	188	**G2**	**0.959**	**G2.1**	**0.959**	0.041	**0.959**	--	--	**0.949**	0.051
Brunnenbirne	14-003-1028	Büron	189	**G1**	0.698	**G1.2**	**0.967**	0.698	0.302	0.033	**0.967**	--	--
Legibirne	14-003-366	Dürrenäsch	189	**G1**	0.698	**G1.2**	**0.967**	0.698	0.302	0.033	**0.967**	--	--
Sal102	14-003-2577	Manno	190	**G1**	**0.960**	**G1.2**	**0.960**	**0.960**	0.040	0.104	**0.896**	--	--
Precoci piccole	14-003-2555	Manno	191	**G2**	0.627	**G2.2**	0.796	0.373	0.627	--	--	0.204	0.796
Precoci grandi	14-003-2556	Manno	192	**G2**	**0.847**	**G2.1**	**0.847**	0.153	**0.847**	--	--	0.570	0.430
Pero Muzzano	14-003-2565	Manno	193	**G1**	**0.954**	**G1.1**	**0.954**	**0.954**	0.046	**0.934**	0.066	--	--
Pero Lugaggia	14-003-2561	Manno	194	**G2**	**0.889**	**G2.1**	**0.889**	0.111	**0.889**	--	--	0.550	0.450
Pero Bero bergamotto	14-003-2558	Manno	195	**G2**	0.585	**G2.2**	0.616	0.415	0.585	--	--	0.384	0.616
Pero piccolo	14-003-2559	Manno	196	**G1**	0.794	**G1.1**	0.794	0.794	0.206	0.788	0.212	--	--
Per rügin	14-003-2571	Manno	197	**G1**	0.534	**G1.1**	0.651	0.534	0.466	0.651	0.349	--	--
Sconosciuto prob. locale	14-003-2579	Manno	198	**G1**	**0.975**	**G1.1**	**0.975**	**0.975**	0.025	**0.876**	0.124	--	--
Pero San Pietro	14-003-2572	Manno	199	**G1**	**0.899**	**G1.1**	**0.913**	**0.899**	0.101	**0.913**	0.087	--	--
Pero Regazzoli	14-003-2715	Manno	200	**G1**	**0.868**	**G1.2**	**0.868**	**0.868**	0.132	0.265	0.735	--	--
Pero Orizzonte	14-003-2716	Manno	201	**G1**	**0.957**	**G1.2**	**0.957**	**0.957**	0.043	0.356	0.644	--	--
Pero Breganzona	14-003-2563	Manno	202	**G1**	**0.961**	**G1.1**	**0.961**	**0.961**	0.039	0.669	0.331	--	--
Martin sec	14-003-2562	Manno	203	**G1**	0.745	**G1.2**	**0.966**	0.745	0.255	0.034	**0.966**	--	--
Piccole	14-003-2557	Manno	204	**G1**	**0.956**	**G1.2**	**0.956**	**0.956**	0.044	0.241	0.759	--	--
Pero prato grande	14-003-2696	Manno	205	**G1**	**0.922**	**G1.2**	**0.922**	**0.922**	0.078	0.177	**0.823**	--	--
Pero Bironico	14-003-2711	Manno	206	**G2**	**0.801**	**G2.2**	**0.801**	0.199	**0.801**	--	--	0.481	0.519
Sconosciuto prob. locale	14-003-2593	Manno	207	**G2**	**0.803**	**G2.2**	**0.803**	0.197	**0.803**	--	--	0.418	0.582
Sconosciuto prob. locale	14-003-2591	Manno	208	**G1**	0.789	**G1.2**	0.789	0.789	0.211	0.316	0.684	--	--
Sconosciuto prob. locale	14-003-2594	Manno	209	**G1**	**0.981**	**G1.1**	**0.981**	**0.981**	0.019	**0.976**	0.024	--	--
Brütt e bòn	14-003-2586	Manno	210	**G1**	**0.804**	**G1.2**	**0.964**	**0.804**	0.196	0.036	**0.964**	--	--
Sconosciuto prob. locale	14-003-2582	Manno	211	**G1**	**0.917**	**G1.1**	**0.917**	**0.917**	0.083	0.678	0.322	--	--
Sconosciuto prob. locale	14-003-2584	Manno	212	**G1**	**0.967**	**G1.2**	**0.967**	**0.967**	0.033	0.470	0.530	--	--
Pér de la Èma	14-003-2588	Manno	213	**G1**	**0.833**	**G1.1**	**0.833**	**0.833**	0.167	0.735	0.265	--	--
Sconosciuto locale	14-003-2581	Manno	214	**G1**	0.654	**G1.1**	0.654	0.654	0.346	0.525	0.475	--	--
Pero grande sinistra	14-003-2698	Manno	215	**G1**	**0.963**	**G1.2**	**0.963**	**0.963**	0.037	0.328	0.672	--	--
Pero a spagliera	14-003-2700	Manno	216	**G1**	**0.952**	**G1.1**	**0.952**	**0.952**	0.048	**0.939**	0.061	--	--
Pero burroso	14-003-2701	Manno	217	**G1**	**0.971**	**G1.1**	**0.971**	**0.971**	0.029	**0.961**	0.039	--	--
Tipo Sept en gueule	14-003-2702	Manno	218	**G1**	**0.801**	**G1.2**	**0.801**	**0.801**	0.199	0.487	0.513	--	--
Pero malmesso	14-003-2703	Manno	219	**G1**	0.665	**G1.1**	0.716	0.665	0.335	0.716	0.284	--	--
Pisö de la Biava	14-003-2713	Manno	220	**G1**	**0.949**	**G1.2**	**0.949**	**0.949**	0.051	0.067	**0.933**	--	--
Vernin	14-003-2712	Manno	221	**G1**	**0.829**	**G1.2**	**0.829**	**0.829**	0.171	0.448	0.552	--	--
Pero romboidale	14-003-2705	Manno	222	**G1**	0.797	**G1.2**	0.797	0.797	0.203	0.443	0.557	--	--
Pero centenario	14-003-2707	Manno	223	**G1**	**0.974**	**G1.1**	**0.974**	**0.974**	0.026	**0.875**	0.125	--	--
Knollbirne	14-003-337	Nuclear stock	224	**G2**	**0.856**	**G2.2**	**0.856**	0.144	**0.856**	--	--	0.144	**0.856**
Wybirne	14-003-738	Roggwil Riedern	225	**G1**	0.550	**G1.2**	0.672	0.550	0.450	0.328	0.672	--	--
unknown	14-003-1505	Roggwil Riedern	226	**G1**	**0.898**	**G1.1**	**0.922**	**0.898**	0.102	**0.922**	0.078	--	--
Minister Dr. Lucius	14-003-406	Roggwil Riedern	227	**G1**	0.796	**G1.1**	**0.932**	0.796	0.204	**0.932**	0.068	--	--
Regelbirne	14-003-1229	Roggwil Riedern	228	**G2**	**0.979**	**G2.2**	**0.979**	0.021	**0.979**	--	--	0.347	0.653
Hasenbirrli	14-003-721	Roggwil Riedern	229	**G1**	0.691	**G1.2**	0.691	0.691	0.309	0.329	0.671	--	--
Gute Graue	14-003-703	Roggwil Riedern	230	**G2**	**0.879**	**G2.2**	**0.879**	0.121	**0.879**	--	--	0.231	0.769
Müli Birne	14-003-709	Roggwil Riedern	231	**G2**	0.643	**G2.1**	0.662	0.357	0.643	--	--	0.662	0.338
Weidbirli	14-003-1487	Roggwil Riedern	232	**G2**	**0.919**	**G2.1**	**0.919**	0.081	**0.919**	--	--	0.551	0.449
Hungbirli	14-003-965	Roggwil Riedern	233	**G2**	**0.852**	**G2.1**	**0.852**	0.148	**0.852**	--	--	0.569	0.431
Züribirne	14-003-1008	Roggwil Riedern	234	**G2**	**0.968**	**G2.1**	**0.968**	0.032	**0.968**	--	--	0.719	0.281
Sürler	14-003-1017	Roggwil Riedern	235	**G2**	**0.864**	**G2.2**	**0.864**	0.136	**0.864**	--	--	0.264	0.736

Häsler	14-003-1032	Roggwil Riedern	236	**G2**	0.774	**G2.2**	**0.937**	0.226	0.774	--	--	0.063	**0.937**
Aglischerl	14-003-1048	Roggwil Riedern	237	**G2**	**0.980**	**G2.2**	**0.980**	0.020	**0.980**	--	--	0.127	**0.873**
Solaner	14-003-1049	Roggwil Riedern	238	**G1**	**0.973**	**G1.2**	**0.973**	**0.973**	0.027	0.488	0.512	--	--
Maria Magdalena	14-003-1064	Roggwil Riedern	239	**G1**	0.718	**G1.2**	**0.970**	0.718	0.282	0.030	**0.970**	--	--
Chugelibire	14-003-1075	Roggwil Riedern	240	**G2**	0.751	**G2.1**	0.751	0.249	0.751	--	--	0.631	0.369
unknown	14-003-1081	Roggwil Riedern	241	**G1**	**0.975**	**G1.1**	**0.975**	**0.975**	0.025	**0.974**	0.026	--	--
unknown	14-003-1087	Roggwil Riedern	242	**G1**	0.523	**G1.2**	**0.972**	0.523	0.477	0.028	**0.972**	--	--
Chäs Bire	14-003-1112	Roggwil Riedern	243	**G1**	**0.983**	**G1.2**	**0.983**	**0.983**	0.017	0.114	**0.886**	--	--
Butterbirne	14-003-1219	Roggwil Riedern	244	**G1**	**0.979**	**G1.1**	**0.979**	**0.979**	0.021	**0.976**	0.024	--	--
Graubirne	14-003-1235	Roggwil Riedern	245	**G2**	**0.940**	**G2.2**	**0.940**	0.060	**0.940**	--	--	0.347	0.653
Kügelibire	14-003-1265	Roggwil Riedern	246	**G1**	**0.955**	**G1.1**	**0.955**	**0.955**	0.045	**0.907**	0.093	--	--
Rebenbirne	14-003-1366	Roggwil Riedern	247	**G2**	0.789	**G2.1**	0.789	0.211	0.789	--	--	0.681	0.319
Blutbirne	14-003-1367	Roggwil Riedern	248	**G1**	**0.854**	**G1.2**	**0.967**	**0.854**	0.146	0.033	**0.967**	--	--
Echte Dornbirne	14-003-1368	Roggwil Riedern	249	**G2**	0.789	**G2.1**	0.789	0.211	0.789	--	--	0.665	0.335
Chogelibere	14-003-1369	Roggwil Riedern	250	**G1**	**0.916**	**G1.1**	**0.916**	**0.916**	0.084	**0.877**	0.123	--	--
Mollebusch	14-003-1370	Roggwil Riedern	251	**G1**	**0.831**	**G1.1**	**0.831**	**0.831**	0.169	0.766	0.234	--	--
Müsler	14-003-729	Roggwil Riedern	252	**G2**	0.759	**G2.2**	0.759	0.241	0.759	--	--	0.298	0.702
Erntbirli	14-003-1384	Roggwil Riedern	253	**G2**	0.785	**G2.2**	0.785	0.215	0.785	--	--	0.378	0.622
Holzerbirne	14-003-1386	Roggwil Riedern	254	**G1**	**0.972**	**G1.1**	**0.972**	**0.972**	0.028	0.694	0.306	--	--
Apothekerbirne	14-003-734	Roggwil Riedern	255	**G1**	**0.879**	**G1.1**	**0.950**	**0.879**	0.121	**0.950**	0.050	--	--
Künstberli	14-003-1397	Roggwil Riedern	256	**G2**	**0.883**	**G2.2**	**0.883**	0.117	**0.883**	--	--	0.251	0.749
Rebbirne	14-003-736	Roggwil Riedern	257	**G2**	**0.900**	**G2.2**	**0.900**	0.100	**0.900**	--	--	0.201	0.799
Wiibeere	14-003-1403	Roggwil Riedern	258	**G2**	**0.977**	**G2.2**	**0.977**	0.023	**0.977**	--	--	0.372	0.628
Winterbirne	14-003-1406	Roggwil Riedern	259	**G2**	**0.957**	**G2.1**	**0.957**	0.043	**0.957**	--	--	0.579	0.421
Zam Reiholzbirne	14-003-1407	Roggwil Riedern	260	**G2**	**0.957**	**G2.2**	**0.957**	0.043	**0.957**	--	--	0.386	0.614
Späte Weinbirne	14-003-1426	Roggwil Riedern	261	**G2**	**0.968**	**G2.2**	**0.968**	0.032	**0.968**	--	--	0.357	0.643
Zuckerbirne	14-003-1430	Roggwil Riedern	262	**G2**	**0.896**	**G2.1**	**0.896**	0.104	**0.896**	--	--	0.557	0.443
Teigbirne	14-003-1440	Roggwil Riedern	263	**G2**	**0.966**	**G2.2**	**0.966**	0.034	**0.966**	--	--	0.058	**0.942**
Grüne Magdalener/Julibirne	14-003-271	Roggwil Riedern	264	**G1**	**0.972**	**G1.1**	**0.972**	**0.972**	0.028	0.698	0.302	--	--
Kollersbirne	14-003-745	Roggwil Riedern	265	**G1**	**0.965**	**G1.1**	**0.966**	**0.965**	0.035	**0.966**	0.034	--	--
Gröler	14-003-747	Roggwil Riedern	266	**G2**	0.678	**G2.2**	0.709	0.322	0.678	--	--	0.291	0.709
Spätmöstler	14-003-748	Roggwil Riedern	267	**G2**	**0.879**	**G2.1**	**0.879**	0.121	**0.879**	--	--	0.756	0.244
Hasenbirne	14-003-290	Roggwil Riedern	268	**G1**	**0.976**	**G1.2**	**0.976**	**0.976**	0.024	0.104	**0.896**	--	--
Happerswiler	14-003-751	Roggwil Riedern	269	**G2**	**0.945**	**G2.1**	**0.945**	0.055	**0.945**	--	--	0.598	0.402
Rüthibirne	14-003-756	Roggwil Riedern	270	**G2**	**0.958**	**G2.2**	**0.958**	0.042	**0.958**	--	--	0.273	0.727
Grünbirne	14-003-757	Roggwil Riedern	271	**G2**	**0.911**	**G2.1**	**0.911**	0.089	**0.911**	--	--	0.572	0.428
Biessenhofer Mostbirne	14-003-58	Roggwil Riedern	272	**G2**	**0.824**	**G2.2**	**0.824**	0.176	**0.824**	--	--	0.306	0.694
Gelbwiegler	14-003-236	Roggwil Riedern	273	**G2**	0.705	**G2.2**	**0.867**	0.295	0.705	--	--	0.133	**0.867**
Julibirne	14-003-767	Roggwil Riedern	274	**G1**	**0.968**	**G1.1**	**0.968**	**0.968**	0.032	**0.837**	0.163	--	--
Sauerbirne	14-003-769	Roggwil Riedern	275	**G2**	**0.938**	**G2.2**	**0.938**	0.062	**0.938**	--	--	0.177	**0.823**
Butterbirne	14-003-770	Roggwil Riedern	276	**G1**	**0.971**	**G1.1**	**0.978**	**0.971**	0.029	**0.978**	0.022	--	--
Ulmer Butterbirne	14-003-637	Roggwil Riedern	277	**G1**	**0.952**	**G1.1**	**0.952**	**0.952**	0.048	**0.869**	0.131	--	--
Kollersbirne	14-003-340	Roggwil Riedern	278	**G2**	**0.957**	**G2.1**	**0.957**	0.043	**0.957**	--	--	**0.877**	0.123
Chlausenbirne	14-003-718	Roggwil Riedern	279	**G1**	**0.915**	**G1.1**	**0.967**	**0.915**	0.085	**0.967**	0.033	--	--
Zuckerbirne	14-003-786	Roggwil Riedern	280	**G1**	**0.980**	**G1.1**	**0.986**	**0.980**	0.020	**0.986**	0.014	--	--
Trübler	14-003-634	Roggwil Riedern	281	**G2**	**0.935**	**G2.2**	**0.935**	0.065	**0.935**	--	--	0.107	**0.893**
Petersbirne	14-003-457	Roggwil Riedern	282	**G1**	0.723	**G1.2**	**0.937**	0.723	0.277	0.063	**0.937**	--	--
Längler	14-003-800	Roggwil Riedern	283	**G2**	**0.980**	**G2.1**	**0.980**	0.020	**0.980**	--	--	**0.950**	0.050
Rotlängler Dörrbirne	14-003-526	Roggwil Riedern	284	**G2**	**0.940**	**G2.1**	**0.940**	0.060	**0.940**	--	--	0.633	0.367
Strickbirne	14-003-601	Roggwil Riedern	285	**G2**	**0.942**	**G2.2**	**0.942**	0.058	**0.942**	--	--	0.314	0.686
Römische Schmalzbirne	14-003-510	Roggwil Riedern	286	**G1**	**0.938**	**G1.2**	**0.976**	**0.938**	0.062	0.024	**0.976**	--	--
unknown	14-003-1460	Roggwil Riedern	287	**G1**	**0.980**	**G1.2**	**0.980**	**0.980**	0.020	0.030	**0.970**	--	--
Müsler	14-003-426	Roggwil Riedern	288	**G2**	0.753	**G2.2**	0.753	0.247	0.753	--	--	0.421	0.579
Zuckerbirne	14-003-891	Roggwil Riedern	289	**G1**	**0.810**	**G1.2**	**0.967**	**0.810**	0.190	0.033	**0.967**	--	--
Rietbirne	14-003-1469	Roggwil Riedern	290	**G2**	**0.954**	**G2.1**	**0.954**	0.046	**0.954**	--	--	**0.919**	0.081
unknown	14-003-1470	Roggwil Riedern	291	**G2**	**0.928**	**G2.2**	**0.928**	0.072	**0.928**	--	--	0.158	**0.842**
Frauenschenkel	14-003-1055	Roggwil Riedern	292	**G1**	0.527	**G1.1**	0.698	0.527	0.473	0.698	0.302	--	--
unknown	14-003-1434	Roggwil Riedern	293	**G2**	**0.976**	**G2.2**	**0.976**	0.024	**0.976**	--	--	0.408	0.592
Julibirne	14-003-713	Roggwil Riedern	294	**G1**	0.705	**G1.2**	**0.965**	0.705	0.295	0.035	**0.965**	--	--
Zuckerbirne	14-003-754	Roggwil Riedern	295	**G2**	**0.957**	**G2.2**	**0.957**	0.043	**0.957**	--	--	0.415	0.585
Bündnertrübla	14-003-771	Roggwil Riedern	296	**G2**	**0.923**	**G2.1**	**0.923**	0.077	**0.923**	--	--	0.634	0.366
Weissbirne	14-003-832	Roggwil Riedern	297	**G1**	0.534	**G1.2**	**0.960**	0.534	0.466	0.040	**0.960**	--	--
Kuhbirne	14-003-844	Roggwil Riedern	298	**G2**	0.775	**G2.2**	**0.888**	0.225	0.775	--	--	0.112	**0.888**
Herrenbirne	14-003-848	Roggwil Riedern	299	**G2**	**0.884**	**G2.1**	**0.884**	0.116	**0.884**	--	--	0.572	0.428
Böxler	14-003-850	Roggwil Riedern	300	**G2**	**0.970**	**G2.2**	**0.970**	0.030	**0.970**	--	--	0.487	0.513
Zimtbirne	14-003-851	Roggwil Riedern	301	**G1**	0.711	**G1.2**	**0.961**	0.711	0.289	0.039	**0.961**	--	--
unknown	14-003-1461	Roggwil Riedern	302	**G1**	**0.983**	**G1.1**	**0.988**	**0.983**	0.017	**0.988**	0.012	--	--
Vrenäbirä	14-003-852	Roggwil Riedern	303	**G2**	0.539	**G2.1**	0.539	0.461	0.539	--	--	0.528	0.472
Schutzibirne	14-003-3752	Roggwil Riedern	304	**G2**	**0.967**	**G2.2**	**0.967**	0.033	**0.967**	--	--	0.384	0.616
Kugelbirne	14-003-853	Roggwil Riedern	305	**G2**	**0.959**	**G2.1**	**0.959**	0.041	**0.959**	--	--	**0.888**	0.112
Marxenbirne	14-003-396	Roggwil Riedern	306	**G2**	**0.974**	**G2.1**	**0.974**	0.026	**0.974**	--	--	**0.860**	0.140
Rotholzbirne	14-003-855	Roggwil Riedern	307	**G2**	**0.966**	**G2.2**	**0.966**	0.034	**0.966**	--	--	0.362	0.638
Helegger	14-003-294	Roggwil Riedern	308	**G2**	**0.967**	**G2.2**	**0.967**	0.033	**0.967**	--	--	0.389	0.611
Rebenbirne	14-003-857	Roggwil Riedern	309	**G2**	**0.841**	**G2.1**	**0.841**	0.159	**0.841**	--	--	**0.814**	0.186
Rothäfler	14-003-859	Roggwil Riedern	310	**G2**	**0.889**	**G2.2**	**0.889**	0.111	**0.889**	--	--	0.155	**0.845**
Räuchlisberger	14-003-860	Roggwil Riedern	311	**G2**	0.533	**G2.2**	0.793	0.467	0.533	--	--	0.207	0.793
Rotlängler	14-003-862	Roggwil Riedern	312	**G2**	**0.874**	**G2.2**	**0.874**	0.126	**0.874**	--	--	0.497	0.503
Thorbirne	14-003-864	Roggwil Riedern	313	**G2**	**0.896**	**G2.1**	**0.896**	0.104	**0.896**	--	--	0.574	0.426
Schorne	14-003-866	Roggwil Riedern	314	**G1**	**0.918**	**G1.1**	**0.956**	**0.918**	0.082	**0.956**	0.044	--	--
Rotholzbirne	14-003-870	Roggwil Riedern	315	**G1**	**0.935**	**G1.1**	**0.957**	**0.935**	0.065	**0.957**	0.043	--	--
Mostbirne	14-003-871	Roggwil Riedern	316	**G2**	0.732	**G2.1**	0.738	0.268	0.732	--	--	0.738	0.262
Welsch Glöggler	14-003-873	Roggwil Riedern	317	**G2**	**0.939**	**G2.1**	**0.939**	0.061	**0.939**	--	--	0.641	0.359
Zitronenbirne	14-003-874	Roggwil Riedern	318	**G2**	**0.944**	**G2.1**	**0.950**	0.056	**0.944**	--	--	**0.950**	0.050
Lebruns Butterbirne	14-003-363	Roggwil Riedern	319	**G1**	0.663	**G1.1**	**0.875**	0.663	0.337	**0.875**	0.125	--	--
Feigenbirne	14-003-879	Roggwil Riedern	320	**G2**	**0.957**	**G2.1**	**0.957**	0.043	**0.957**	--	--	0.728	0.272
Gunteschhuser	14-003-880	Roggwil Riedern	321	**G2**	0.790	**G2.2**	0.790	0.210	0.790	--	--	0.445	0.555
Holzbirne	14-003-943	Roggwil Riedern	322	**G2**	**0.922**	**G2.2**	**0.922**	0.078	**0.922**	--	--	0.093	**0.907**

Magdalenen	14-003-881	Roggwil Riedern	323	**G1**	0.703	**G1.2**	**0.955**	0.703	0.297	0.045	**0.955**	--	--
Feigenbirne	14-003-882	Roggwil Riedern	324	**G2**	**0.982**	**G2.1**	**0.982**	0.018	**0.982**	--	--	0.501	0.499
Zuckerbirne	14-003-1464	Roggwil Riedern	325	**G2**	**0.946**	**G2.1**	**0.946**	0.054	**0.946**	--	--	**0.853**	0.147
unknown	14-003-1465	Roggwil Riedern	326	**G1**	**0.915**	**G1.2**	**0.979**	**0.915**	0.085	0.021	**0.979**	--	--
Trübler	14-003-1466	Roggwil Riedern	327	**G2**	**0.903**	**G2.1**	**0.903**	0.097	**0.903**	--	--	0.751	0.249
Zuckerbirne	14-003-885	Roggwil Riedern	328	**G2**	**0.961**	**G2.1**	**0.961**	0.039	**0.961**	--	--	0.589	0.411
Biessenhofer Birne	14-003-887	Roggwil Riedern	329	**G2**	0.692	**G2.2**	**0.823**	0.308	0.692	--	--	0.177	**0.823**
Schmalzbirne von Brest/Heubirne	14-003-557	Roggwil Riedern	330	**G2**	**0.958**	**G2.1**	**0.958**	0.042	**0.958**	--	--	**0.867**	0.133
Landsknechtler	14-003-555	Roggwil Riedern	331	**G2**	**0.908**	**G2.1**	**0.933**	0.092	**0.908**	--	--	**0.933**	0.067
Chriesibirne	14-003-889	Roggwil Riedern	332	**G2**	**0.962**	**G2.1**	**0.962**	0.038	**0.962**	--	--	**0.823**	0.177
Feigenbirne	14-003-892	Roggwil Riedern	333	**G2**	0.787	**G2.1**	0.787	0.213	0.787	--	--	0.609	0.391
Trischeläbere	14-003-626	Roggwil Riedern	334	**G2**	**0.910**	**G2.1**	**0.910**	0.090	**0.910**	--	--	0.713	0.287
Letterbirne	14-003-893	Roggwil Riedern	335	**G2**	0.632	**G2.2**	**0.927**	0.368	0.632	--	--	0.073	**0.927**
Kammerbirne	14-003-894	Roggwil Riedern	336	**G2**	**0.955**	**G2.1**	**0.955**	0.045	**0.955**	--	--	**0.918**	0.082
Mehlbirne	14-003-896	Roggwil Riedern	337	**G2**	**0.980**	**G2.2**	**0.980**	0.020	**0.980**	--	--	0.265	0.735
Aeulisbirne	14-003-897	Roggwil Riedern	338	**G2**	**0.958**	**G2.1**	**0.958**	0.042	**0.958**	--	--	**0.816**	0.184
Rütibirne	14-003-898	Roggwil Riedern	339	**G2**	0.589	**G2.2**	**0.904**	0.411	0.589	--	--	0.096	**0.904**
Happerswiler	14-003-899	Roggwil Riedern	340	**G1**	0.770	**G1.2**	**0.936**	0.770	0.230	0.064	**0.936**	--	--
Butterbirne	14-003-901	Roggwil Riedern	341	**G2**	**0.873**	**G2.1**	**0.873**	0.127	**0.873**	--	--	0.547	0.453
Zitronenbirne	14-003-903	Roggwil Riedern	342	**G1**	0.700	**G1.2**	**0.968**	0.700	0.300	0.032	**0.968**	--	--
Muggenbirne	14-003-905	Roggwil Riedern	343	**G2**	**0.883**	**G2.2**	**0.883**	0.117	**0.883**	--	--	0.342	0.658
Blaulängala	14-003-906	Roggwil Riedern	344	**G2**	**0.912**	**G2.1**	**0.912**	0.088	**0.912**	--	--	0.504	0.496
Juxbirne	14-003-909	Roggwil Riedern	345	**G1**	0.706	**G1.2**	**0.945**	0.706	0.294	0.055	**0.945**	--	--
Winterlenkelen	14-003-910	Roggwil Riedern	346	**G2**	**0.977**	**G2.1**	**0.977**	0.023	**0.977**	--	--	**0.851**	0.149
unknown	14-003-1468	Roggwil Riedern	347	**G1**	0.542	**G1.2**	**0.915**	0.542	0.458	0.085	**0.915**	--	--
Königsbirne	14-003-913	Roggwil Riedern	348	**G1**	**0.826**	**G1.1**	**0.832**	**0.826**	0.174	**0.832**	0.168	--	--
Affelträngler	14-003-916	Roggwil Riedern	349	**G2**	0.651	**G2.2**	**0.888**	0.349	0.651	--	--	0.112	**0.888**
Herbstgütler	14-003-298	Roggwil Riedern	350	**G2**	0.532	**G2.2**	0.564	0.468	0.532	--	--	0.436	0.564
Spitzbirne	14-003-717	Roggwil Riedern	351	**G2**	**0.938**	**G2.2**	**0.938**	0.062	**0.938**	--	--	0.252	0.748
Betzelsbirne	14-003-719	Roggwil Riedern	352	**G2**	**0.864**	**G2.1**	**0.864**	0.136	**0.864**	--	--	0.513	0.487
Fleischbirne	14-003-926	Roggwil Riedern	353	**G2**	**0.959**	**G2.1**	**0.959**	0.041	**0.959**	--	--	**0.913**	0.087
Mostbirne	14-003-928	Roggwil Riedern	354	**G2**	**0.930**	**G2.1**	**0.930**	0.070	**0.930**	--	--	**0.925**	0.075
unknown	14-003-929	Roggwil Riedern	355	**G2**	**0.961**	**G2.1**	**0.961**	0.039	**0.961**	--	--	0.783	0.217
unknown	14-003-1477	Roggwil Riedern	356	**G2**	**0.940**	**G2.1**	**0.940**	0.060	**0.940**	--	--	0.559	0.441
Grünmöstler	14-003-931	Roggwil Riedern	357	**G2**	**0.935**	**G2.2**	**0.935**	0.065	**0.935**	--	--	0.435	0.565
Napoleons Butterbirne	14-003-429	Roggwil Riedern	358	**G1**	**0.974**	**G1.1**	**0.981**	**0.974**	0.026	**0.981**	0.019	--	--
Zitronenbirne	14-003-935	Roggwil Riedern	359	**G2**	**0.937**	**G2.1**	**0.937**	0.063	**0.937**	--	--	0.569	0.431
Zitronenbirne	14-003-937	Roggwil Riedern	360	**G1**	**0.980**	**G1.1**	**0.983**	**0.980**	0.020	**0.983**	0.017	--	--
Happerswiler	14-003-938	Roggwil Riedern	361	**G2**	**0.807**	**G2.2**	**0.807**	0.193	**0.807**	--	--	0.375	0.625
unknown	14-003-1480	Roggwil Riedern	362	**G2**	**0.964**	**G2.1**	**0.964**	0.036	**0.964**	--	--	**0.912**	0.088
unknown	14-003-1481	Roggwil Riedern	363	**G1**	**0.966**	**G1.1**	**0.966**	**0.966**	0.034	**0.939**	0.061	--	--
Mostbirne	14-003-939	Roggwil Riedern	364	**G1**	0.532	**G1.2**	**0.973**	0.532	0.468	0.027	**0.973**	--	--
Butterbirne	14-003-940	Roggwil Riedern	365	**G1**	**0.894**	**G1.1**	**0.894**	**0.894**	0.106	0.679	0.321	--	--
Fischerbirne	14-003-941	Roggwil Riedern	366	**G2**	**0.930**	**G2.2**	**0.930**	0.070	**0.930**	--	--	0.252	0.748
Fleischbirne	14-003-1012	Roggwil Riedern	367	**G1**	0.523	**G1.2**	**0.943**	0.523	0.477	0.057	**0.943**	--	--
Bündner Langbirne	14-003-947	Roggwil Riedern	368	**G2**	**0.958**	**G2.1**	**0.958**	0.042	**0.958**	--	--	**0.914**	0.086
Räberbirli	14-003-955	Roggwil Riedern	369	**G2**	**0.843**	**G2.1**	**0.843**	0.157	**0.843**	--	--	0.624	0.376
Martibirne	14-003-959	Roggwil Riedern	370	**G2**	**0.978**	**G2.2**	**0.978**	0.022	**0.978**	--	--	0.186	**0.814**
Fleischbirne	14-003-962	Roggwil Riedern	371	**G2**	**0.931**	**G2.1**	**0.931**	0.069	**0.931**	--	--	0.676	0.324
Winterbirne	14-003-967	Roggwil Riedern	372	**G1**	0.627	**G1.2**	0.780	0.627	0.373	0.220	0.780	--	--
Weinbirne	14-003-971	Roggwil Riedern	373	**G2**	**0.972**	**G2.2**	**0.972**	0.028	**0.972**	--	--	0.351	0.649
Kunstbirne	14-003-972	Roggwil Riedern	374	**G2**	**0.962**	**G2.1**	**0.962**	0.038	**0.962**	--	--	**0.943**	0.057
saure Kugelbirne	14-003-978	Roggwil Riedern	375	**G2**	0.799	**G2.2**	**0.821**	0.201	0.799	--	--	0.179	**0.821**
Martinsbirne	14-003-358	Roggwil Riedern	376	**G2**	**0.935**	**G2.1**	**0.935**	0.065	**0.935**	--	--	**0.878**	0.122
Schwarzrädler	14-003-563	Roggwil Riedern	377	**G2**	**0.969**	**G2.1**	**0.969**	0.031	**0.969**	--	--	0.652	0.348
Rossaletti	14-003-1003	Roggwil Riedern	378	**G1**	**0.900**	**G1.2**	**0.973**	**0.900**	0.100	0.027	**0.973**	--	--
Blutbirne	14-003-1004	Roggwil Riedern	379	**G1**	0.575	**G1.2**	**0.929**	0.575	0.425	0.071	**0.929**	--	--
Lederbirne	14-003-1006	Roggwil Riedern	380	**G2**	0.751	**G2.1**	0.751	0.249	0.751	--	--	0.717	0.283
Ackerbirne	14-003-1495	Roggwil Riedern	381	**G2**	**0.913**	**G2.2**	**0.913**	0.087	**0.913**	--	--	0.281	0.719
Melone	14-003-1096	Roggwil Riedern	382	**G2**	**0.968**	**G2.1**	**0.968**	0.032	**0.968**	--	--	**0.948**	0.052
Züri-Birne	14-003-1410	Roggwil Riedern	383	**G2**	**0.883**	**G2.2**	**0.883**	0.117	**0.883**	--	--	0.315	0.685
Häsler	14-003-730	Roggwil Riedern	384	**G2**	**0.934**	**G2.2**	**0.934**	0.066	**0.934**	--	--	0.151	**0.849**
Heubirli	14-003-1418	Roggwil Riedern	385	**G1**	**0.820**	**G1.2**	**0.931**	**0.820**	0.180	0.069	**0.931**	--	--
Goyatzon	14-003-1522	Roggwil Riedern	386	**G2**	0.796	**G2.1**	0.796	0.204	0.796	--	--	0.557	0.443
Merode2	14-003-2570	Roggwil Riedern	387	**G1**	0.761	**G1.1**	0.761	0.761	0.239	0.658	0.342	--	--
Merode1	14-003-1544	Roggwil Riedern	388	**G1**	**0.944**	**G1.1**	**0.944**	**0.944**	0.056	0.512	0.488	--	--
Pétolin	14-003-1567	Roggwil Riedern	389	**G1**	0.511	**G1.2**	**0.956**	0.511	0.489	0.044	**0.956**	--	--
Petit Gris / Martinsec	14-003-1578	Roggwil Riedern	390	**G1**	0.620	**G1.1**	0.620	0.620	0.380	0.517	0.483	--	--
Chriesibirne Thundorf	14-003-2569	Roggwil Riedern	391	**G2**	0.769	**G2.1**	0.769	0.231	0.769	--	--	0.580	0.420
Schutzibirne	14-003-561	Höri	392	**G2**	**0.944**	**G2.2**	**0.944**	0.056	**0.944**	--	--	0.478	0.522
Palmischbirne	14-003-446	Höri	393	**G1**	0.524	**G1.1**	0.606	0.524	0.476	0.606	0.394	--	--
Schweizer Bratbirne	14-003-564	Höri	394	**G2**	**0.981**	**G2.1**	**0.981**	0.019	**0.981**	--	--	0.576	0.424
Guntershauser	14-003-280	Höri	395	**G2**	**0.931**	**G2.2**	**0.931**	0.069	**0.931**	--	--	0.357	0.643
Ottenbacher Schellerbirne	14-003-444	Höri	396	**G2**	**0.962**	**G2.1**	**0.962**	0.038	**0.962**	--	--	0.518	0.482
Sommereierbirne	14-003-581	Höri	397	**G2**	**0.802**	**G2.1**	**0.802**	0.198	**0.802**	--	--	0.712	0.288
Schwyzerhose	14-003-568	Höri	398	**G2**	0.658	**G2.2**	0.762	0.342	0.658	--	--	0.238	0.762
Ulmer Butterbirne	14-003-3640	Höri	399	**G2**	0.538	**G2.1**	0.696	0.462	0.538	--	--	0.696	0.304
Rheinholzbirne	14-003-498	Höri	400	**G2**	0.758	**G2.2**	0.772	0.242	0.758	--	--	0.228	0.772
Herbstlängler	14-003-299	Höri	401	**G2**	**0.948**	**G2.1**	**0.948**	0.052	**0.948**	--	--	**0.940**	0.060
Langstieler	14-003-356	Höri	402	**G2**	**0.905**	**G2.1**	**0.905**	0.095	**0.905**	--	--	0.703	0.297
Guntershauser	14-003-280	Höri	403	**G2**	0.720	**G2.2**	0.720	0.280	0.720	--	--	0.492	0.508
Schoggolabirne	14-003-599	Höri	404	**G1**	0.743	**G1.2**	**0.811**	0.743	0.257	0.189	**0.811**	--	--
Lugnezer Honigbirne	14-003-375	Höri	405	**G2**	**0.937**	**G2.1**	**0.937**	0.063	**0.937**	--	--	0.781	0.219
Schafbirne	14-003-553	Höri	406	**G2**	**0.978**	**G2.1**	**0.978**	0.022	**0.978**	--	--	0.559	0.441
Schafbirne	14-003-553	Höri	407	**G2**	**0.961**	**G2.2**	**0.961**	0.039	**0.961**	--	--	0.415	0.585
Längler	14-003-3652	Höri	408	**G2**	**0.937**	**G2.2**	**0.937**	0.063	**0.937**	--	--	0.367	0.633
Weldenser	14-003-662	Höri	409	**G1**	0.745	**G1.1**	**0.882**	0.745	0.255	**0.882**	0.118	--	--

Schweizer Bratbirne	14-003-564	Höri	410	**G2**	**0.938**	**G2.1**	**0.938**	0.062	**0.938**	--	--	0.570	0.430
Späte Weinbirne	14-003-3655	Höri	411	**G2**	**0.888**	**G2.2**	**0.888**	0.112	**0.888**	--	--	0.212	0.788
Richlisbirne	14-003-500	Höri	412	**G2**	**0.959**	**G2.2**	**0.959**	0.041	**0.959**	--	--	0.261	0.739
Bergler	14-003-47	Höri	413	**G2**	**0.888**	**G2.2**	**0.888**	0.112	**0.888**	--	--	0.432	0.568
Ottenbacher Schellerbirne	14-003-444	Höri	414	**G2**	**0.960**	**G2.2**	**0.960**	0.040	**0.960**	--	--	0.350	0.650
Ulmer Butterbirne	14-003-3641	Höri	415	**G2**	**0.887**	**G2.2**	**0.887**	0.113	**0.887**	--	--	0.236	0.764
Herbstgütler	14-003-3657	Höri	416	**G2**	0.543	**G2.2**	0.563	0.457	0.543	--	--	0.437	0.563
Goldschmeckler	14-003-253	Höri	417	**G2**	**0.826**	**G2.2**	**0.826**	0.174	**0.826**	--	--	0.478	0.522
Luzerner Weinbirne	14-003-378	Höri	418	**G1**	0.567	**G1.2**	**0.854**	0.567	0.433	0.146	**0.854**	--	--
Schwarzrädler	14-003-3660	Höri	419	**G2**	**0.971**	**G2.1**	**0.971**	0.029	**0.971**	--	--	0.537	0.463
Ottenbacher Schellerbirne	14-003-444	Höri	420	**G2**	**0.931**	**G2.2**	**0.931**	0.069	**0.931**	--	--	0.464	0.536
Schwarzrädler	14-003-3660	Höri	421	**G2**	**0.971**	**G2.1**	**0.971**	0.029	**0.971**	--	--	0.559	0.441
Landsknechtler	14-003-3661	Höri	422	**G2**	0.737	**G2.1**	0.737	0.263	0.737	--	--	0.639	0.361
Schoggolabirne	14-003-599	Höri	423	**G1**	0.518	**G1.2**	0.794	0.518	0.482	0.206	0.794	--	--
Grünmöstler	14-003-3662	Höri	424	**G2**	**0.967**	**G2.1**	**0.967**	0.033	**0.967**	--	--	0.562	0.438
Luzeiner Längler	14-003-3663	Höri	425	**G2**	**0.977**	**G2.1**	**0.977**	0.023	**0.977**	--	--	**0.904**	0.096
Poire Martin-Sec	14-003-1150	Höri	426	**G1**	0.697	**G1.2**	**0.935**	0.697	0.303	0.065	**0.935**	--	--
Landsknechtler	14-003-3668	Höri	427	**G1**	0.659	**G1.2**	**0.977**	0.659	0.341	0.023	**0.977**	--	--
Lätterbirne	14-003-358	Höri	428	**G2**	**0.940**	**G2.2**	**0.940**	0.060	**0.940**	--	--	0.491	0.509
Frühe Weinbirne	14-003-226	Höri	429	**G1**	0.773	**G1.1**	0.773	0.773	0.227	0.596	0.404	--	--
Längler	14-003-3653	Höri	430	**G2**	**0.965**	**G2.1**	**0.965**	0.035	**0.965**	--	--	**0.954**	0.046
Rotlängler	14-003-3666	Höri	431	**G2**	**0.969**	**G2.1**	**0.969**	0.031	**0.969**	--	--	**0.928**	0.072
Hasenbirne	14-003-3670	Höri	432	**G2**	**0.962**	**G2.2**	**0.962**	0.038	**0.962**	--	--	0.250	0.750
Herbstgütler	14-003-3658	Höri	433	**G2**	0.552	**G2.2**	0.568	0.448	0.552	--	--	0.432	0.568
Blutbirne	14-003-68	Höri	434	**G2**	**0.845**	**G2.1**	**0.845**	0.155	**0.845**	--	--	0.660	0.340
Rotlängler	14-003-3667	Höri	435	**G2**	**0.973**	**G2.1**	**0.973**	0.027	**0.973**	--	--	**0.949**	0.051
unknown	14-003-1267	Koppigen	436	**G2**	0.560	**G2.1**	0.631	0.440	0.560	--	--	0.631	0.369
unknown	14-003-1431	Koppigen	437	**G1**	0.783	**G1.1**	**0.931**	0.783	0.217	**0.931**	0.069	--	--
Ankenbirne	14-003-1271	Koppigen	438	**G1**	0.734	**G1.2**	**0.908**	0.734	0.266	0.092	**0.908**	--	--
unknown	14-003-1230	Koppigen	439	**G2**	**0.935**	**G2.1**	**0.948**	0.065	**0.935**	--	--	**0.948**	0.052
Herrenbirne	14-003-1312	Koppigen	440	**G2**	0.785	**G2.2**	0.785	0.215	0.785	--	--	0.243	0.757
Siegelbirne	14-003-1313	Koppigen	441	**G2**	0.652	**G2.1**	0.800	0.348	0.652	--	--	0.800	0.200
Schneebirne	14-003-1310	Koppigen	442	**G2**	**0.953**	**G2.1**	**0.953**	0.047	**0.953**	--	--	**0.878**	0.122
unknown	14-003-1340	Koppigen	443	**G2**	0.751	**G2.1**	0.751	0.249	0.751	--	--	0.751	0.249
Speckbirne	14-003-1360	Koppigen	444	**G2**	0.575	**G2.1**	0.577	0.425	0.575	--	--	0.577	0.423
Roussonetli	14-003-1354	Koppigen	445	**G1**	**0.914**	**G1.1**	**0.972**	**0.914**	0.086	**0.972**	0.028	--	--
unknown	14-003-1348	Koppigen	446	**G2**	**0.957**	**G2.1**	**0.957**	0.043	**0.957**	--	--	0.568	0.432
unknown	14-003-1332	Koppigen	447	**G2**	**0.973**	**G2.1**	**0.973**	0.027	**0.973**	--	--	0.529	0.471
Schafbirne	14-003-1228	Koppigen	448	**G2**	0.793	**G2.2**	**0.809**	0.207	0.793	--	--	0.191	**0.809**
Bühlbirne	14-003-1257	Koppigen	449	**G2**	**0.894**	**G2.2**	**0.894**	0.106	**0.894**	--	--	0.224	0.776
unknown	14-003-1234	Koppigen	450	**G1**	0.549	**G1.2**	0.701	0.549	0.451	0.299	0.701	--	--
Mutschgentellerbirne	14-003-1106	Koppigen	451	**G2**	0.597	**G2.1**	0.597	0.403	0.597	--	--	0.501	0.499
Herzbirnen	14-003-1233	Koppigen	452	**G2**	**0.970**	**G2.1**	**0.970**	0.030	**0.970**	--	--	**0.836**	0.164
Kugelbirne	14-003-1242	Koppigen	453	**G2**	**0.962**	**G2.1**	**0.962**	0.038	**0.962**	--	--	0.721	0.279
Stettlenbirne	14-003-1249	Koppigen	454	**G2**	**0.884**	**G2.1**	**0.884**	0.116	**0.884**	--	--	**0.830**	0.170
Hostettler	14-003-1254	Koppigen	455	**G1**	0.694	**G1.1**	**0.840**	0.694	0.306	**0.840**	0.160	--	--
Sonnenbirne	14-003-1236	Koppigen	456	**G2**	**0.973**	**G2.1**	**0.973**	0.027	**0.973**	--	--	0.687	0.313
Bockbirneli	14-003-1109	Koppigen	457	**G2**	**0.966**	**G2.1**	**0.966**	0.034	**0.966**	--	--	0.645	0.355
Goldtaler	14-003-1387	Koppigen	458	**G2**	**0.918**	**G2.1**	**0.918**	0.082	**0.918**	--	--	0.539	0.461
Augustbeerli	14-003-1097	Koppigen	459	**G2**	0.772	**G2.2**	0.772	0.228	0.772	--	--	0.255	0.745
unknown	14-003-1250	Koppigen	460	**G1**	**0.909**	**G1.1**	**0.948**	**0.909**	0.091	**0.948**	0.052	--	--
Butter	14-003-1220	Koppigen	461	**G1**	0.684	**G1.1**	0.684	0.684	0.316	0.547	0.453	--	--
Gelbbirne	14-003-1241	Koppigen	462	**G2**	**0.963**	**G2.2**	**0.963**	0.037	**0.963**	--	--	0.143	**0.857**
Augustbirne	14-003-1246	Koppigen	463	**G2**	**0.981**	**G2.2**	**0.981**	0.019	**0.981**	--	--	0.463	0.537
Winterbirne	14-003-1114	Koppigen	464	**G2**	0.723	**G2.1**	0.723	0.277	0.723	--	--	0.609	0.391
unknown	14-003-1245	Koppigen	465	**G2**	**0.974**	**G2.2**	**0.974**	0.026	**0.974**	--	--	0.214	0.786
Olives	14-003-1282	Koppigen	466	**G2**	**0.923**	**G2.2**	**0.923**	0.077	**0.923**	--	--	0.342	0.658
Amman	14-003-1221	Koppigen	467	**G1**	**0.883**	**G1.2**	**0.883**	**0.883**	0.117	0.247	0.753	--	--
unknown	14-003-1357	Koppigen	468	**G2**	**0.877**	**G2.1**	**0.877**	0.123	**0.877**	--	--	0.591	0.409
unknown	14-003-1264	Koppigen	469	**G2**	**0.954**	**G2.1**	**0.954**	0.046	**0.954**	--	--	**0.826**	0.174
unknown	14-003-1283	Koppigen	470	**G2**	**0.881**	**G2.1**	**0.881**	0.119	**0.881**	--	--	0.540	0.460
Gartenbirne	14-003-725	Koppigen	471	**G2**	**0.953**	**G2.1**	**0.953**	0.047	**0.953**	--	--	0.609	0.391
unknown	14-003-1356	Koppigen	472	**G2**	**0.929**	**G2.1**	**0.929**	0.071	**0.929**	--	--	0.636	0.364
unknown	14-003-1362	Koppigen	473	**G1**	0.764	**G1.1**	0.764	0.764	0.236	0.707	0.293	--	--
Butterbirne	14-003-1298	Koppigen	474	**G1**	**0.956**	**G1.1**	**0.956**	**0.956**	0.044	**0.889**	0.111	--	--
Butterbirne	14-003-1091	Koppigen	475	**G2**	**0.820**	**G2.1**	**0.820**	0.180	**0.820**	--	--	0.586	0.414
Channebire / Würgebirne	14-003-1108	Koppigen	476	**G2**	**0.917**	**G2.1**	**0.917**	0.083	**0.917**	--	--	0.550	0.450
Bühlbirne	14-003-1274	Koppigen	477	**G2**	**0.834**	**G2.2**	**0.925**	0.166	**0.834**	--	--	0.075	**0.925**
Poire de fer	14-003-1294	Koppigen	478	**G2**	0.796	**G2.1**	0.796	0.204	0.796	--	--	0.681	0.319
Köllikerbirne	14-003-1297	Koppigen	479	**G2**	**0.956**	**G2.1**	**0.956**	0.044	**0.956**	--	--	0.597	0.403
Kodia vert	14-003-1279	Koppigen	480	**G2**	**0.878**	**G2.1**	**0.878**	0.122	**0.878**	--	--	**0.812**	0.188
Coyet	14-003-1296	Koppigen	481	**G2**	**0.886**	**G2.1**	**0.886**	0.114	**0.886**	--	--	0.557	0.443
Winterbirne	14-003-1324	Koppigen	482	**G1**	**0.806**	**G1.1**	**0.851**	**0.806**	0.194	**0.851**	0.149	--	--
Crugelibirnen	14-003-1318	Koppigen	483	**G1**	0.773	**G1.2**	0.773	0.773	0.227	0.330	0.670	--	--
unknown	14-003-1325	Koppigen	484	**G1**	**0.863**	**G1.1**	**0.863**	**0.863**	0.137	0.789	0.211	--	--
Sürler	14-003-732	Koppigen	485	**G2**	**0.907**	**G2.2**	**0.907**	0.093	**0.907**	--	--	0.490	0.510
unknown	14-003-1358	Koppigen	486	**G1**	**0.875**	**G1.1**	**0.875**	**0.875**	0.125	**0.872**	0.128	--	--
Zuckerbirne	14-003-1243	Koppigen	487	**G2**	**0.982**	**G2.2**	**0.982**	0.018	**0.982**	--	--	0.154	**0.846**
Grossmüeti	14-003-1216	Koppigen	488	**G2**	0.659	**G2.1**	0.687	0.341	0.659	--	--	0.687	0.313
unknown	14-003-1349	Koppigen	489	**G1**	**0.970**	**G1.1**	**0.970**	**0.970**	0.030	**0.967**	0.033	--	--
unknown	14-003-1338	Koppigen	490	**G1**	**0.924**	**G1.1**	**0.968**	**0.924**	0.076	**0.968**	0.032	--	--
Farnatte	14-003-1281	Koppigen	491	**G2**	**0.878**	**G2.2**	**0.878**	0.122	**0.878**	--	--	0.242	0.758
Siegelbirne	14-003-1329	Koppigen	492	**G2**	**0.970**	**G2.2**	**0.970**	0.030	**0.970**	--	--	0.478	0.522
Bühler	14-003-1277	Koppigen	493	**G1**	**0.936**	**G1.1**	**0.936**	**0.936**	0.064	0.633	0.367	--	--
unknown	14-003-1350	Koppigen	494	**G2**	0.592	**G2.2**	**0.902**	0.408	0.592	--	--	0.098	**0.902**
Rodje Bougre	14-003-1295	Koppigen	495	**G1**	0.680	**G1.2**	**0.977**	0.680	0.320	0.023	**0.977**	--	--
Dornbirne / Schürbire	14-003-1103	Koppigen	496	**G2**	0.647	**G2.2**	0.760	0.353	0.647	--	--	0.240	0.760

Gätterlibirne	14-003-1105	Koppigen	497	**G1**	0.675	**G1.2**	0.675	0.675	0.325	0.427	0.573	--	--
Schweizer Goldbirne	14-003-1218	Koppigen	498	**G1**	**0.976**	**G1.1**	**0.976**	**0.976**	0.024	**0.936**	0.064	--	--
Herrenbirne / Wagner	14-003-1113	Koppigen	499	**G1**	**0.968**	**G1.1**	**0.979**	**0.968**	0.032	**0.979**	0.021	--	--
unknown	14-003-1361	Koppigen	500	**G2**	**0.813**	**G2.1**	**0.813**	0.187	**0.813**	--	--	0.715	0.285
unknown	14-003-1224	Koppigen	501	**G1**	**0.965**	**G1.1**	**0.965**	**0.965**	0.035	0.721	0.279	--	--
Königs Butterbirne	14-003-1304	Koppigen	502	**G1**	**0.944**	**G1.1**	**0.944**	**0.944**	0.056	**0.921**	0.079	--	--
Weinbirne	14-003-1270	Koppigen	503	**G2**	**0.941**	**G2.2**	**0.941**	0.059	**0.941**	--	--	0.066	**0.934**
Bockbirnen	14-003-1323	Koppigen	504	**G2**	**0.945**	**G2.1**	**0.945**	0.055	**0.945**	--	--	0.683	0.317
unknown	14-003-1308	Koppigen	505	**G1**	**0.854**	**G1.2**	**0.854**	**0.854**	0.146	0.463	0.537	--	--
Heubirne	14-003-1244	Koppigen	506	**G2**	0.598	**G2.2**	**0.829**	0.402	0.598	--	--	0.171	**0.829**
Philipps Butterbirne	14-003-1352	Koppigen	507	**G1**	**0.832**	**G1.1**	**0.832**	**0.832**	0.168	**0.809**	0.191	--	--
unknown	14-003-1292	Koppigen	508	**G2**	**0.897**	**G2.2**	**0.897**	0.103	**0.897**	--	--	0.447	0.553
Holländerbirne	14-003-1287	Koppigen	509	**G2**	**0.821**	**G2.1**	**0.821**	0.179	**0.821**	--	--	0.708	0.292
Butterbirne / Ankebire	14-003-1319	Koppigen	510	**G1**	0.661	**G1.1**	0.661	0.661	0.339	0.604	0.396	--	--
Fivisbirne	14-003-1263	Koppigen	511	**G1**	0.680	**G1.2**	**0.931**	0.680	0.320	0.069	**0.931**	--	--
Schürlibirne	14-003-1268	Koppigen	512	**G2**	**0.952**	**G2.2**	**0.952**	0.048	**0.952**	--	--	0.335	0.665
Speckbirne	14-003-1286	Koppigen	513	**G2**	**0.952**	**G2.1**	**0.952**	0.048	**0.952**	--	--	**0.937**	0.063
Späte	14-003-1111	Koppigen	514	**G1**	**0.854**	**G1.1**	**0.854**	**0.854**	0.146	0.695	0.305	--	--
Birli	14-003-1089	Koppigen	515	**G1**	**0.972**	**G1.1**	**0.972**	**0.972**	0.028	**0.944**	0.056	--	--
Hausbirne / Balisadenbirne	14-003-1276	Koppigen	516	**G2**	0.727	**G2.1**	0.750	0.273	0.727	--	--	0.750	0.250
Grünbirne	14-003-1322	Koppigen	517	**G1**	**0.870**	**G1.2**	**0.874**	**0.870**	0.130	0.126	**0.874**	--	--
Goldforelle	14-003-1300	Koppigen	518	**G1**	**0.968**	**G1.1**	**0.968**	**0.968**	0.032	**0.961**	0.039	--	--
Ankenbirne	14-003-1333	Koppigen	519	**G1**	**0.963**	**G1.2**	**0.963**	**0.963**	0.037	0.102	**0.898**	--	--
Rotfleischiges Birli	14-003-1335	Koppigen	520	**G1**	0.560	**G1.2**	**0.966**	0.560	0.440	0.034	**0.966**	--	--
Augustbirne	14-003-1316	Koppigen	521	**G2**	**0.970**	**G2.2**	**0.970**	0.030	**0.970**	--	--	0.229	0.771
Zitronenbirne	14-003-1305	Koppigen	522	**G1**	0.601	**G1.2**	**0.935**	0.601	0.399	0.065	**0.935**	--	--
Wasserdorn	14-003-1260	Koppigen	523	**G2**	**0.938**	**G2.1**	**0.938**	0.062	**0.938**	--	--	0.564	0.436
unknown	14-003-1240	Koppigen	524	**G1**	**0.909**	**G1.1**	**0.940**	**0.909**	0.091	**0.940**	0.060	--	--
Regelbirne	14-003-1100	Koppigen	525	**G1**	0.534	**G1.2**	**0.971**	0.534	0.466	0.029	**0.971**	--	--
Späte Ankenbirne	14-003-1101	Koppigen	526	**G1**	**0.912**	**G1.1**	**0.975**	**0.912**	0.088	**0.975**	0.025	--	--
Weihnachtsbirne	14-003-1110	Koppigen	527	**G1**	**0.868**	**G1.1**	**0.951**	**0.868**	0.132	**0.951**	0.049	--	--
Butterbirne	14-003-1222	Koppigen	528	**G2**	0.582	**G2.1**	0.582	0.418	0.582	--	--	0.577	0.423
Fleischbirne	14-003-724	Koppigen	529	**G1**	**0.975**	**G1.1**	**0.983**	**0.975**	0.025	**0.983**	0.017	--	--
unknown	14-003-1433	Koppigen	530	**G2**	**0.911**	**G2.1**	**0.911**	0.089	**0.911**	--	--	0.539	0.461
Dörrbirne	14-003-1047	Koppigen	531	**G2**	**0.963**	**G2.2**	**0.963**	0.037	**0.963**	--	--	0.188	**0.812**
unknown	14-003-1285	Koppigen	532	**G1**	0.782	**G1.1**	0.782	0.782	0.218	0.767	0.233	--	--
Berner Wasserbirne	14-003-1251	Koppigen	533	**G2**	**0.968**	**G2.2**	**0.968**	0.032	**0.968**	--	--	0.083	**0.917**
unknown	14-003-1307	Koppigen	534	**G2**	**0.801**	**G2.2**	**0.801**	0.199	**0.801**	--	--	0.466	0.534
Bodmer	14-003-1275	Koppigen	535	**G2**	**0.938**	**G2.1**	**0.938**	0.062	**0.938**	--	--	0.726	0.274
Rägelisbirne	14-003-1303	Koppigen	536	**G1**	**0.921**	**G1.1**	**0.923**	**0.921**	0.079	**0.923**	0.077	--	--
Pfundbirne	14-003-1258	Koppigen	537	**G1**	**0.955**	**G1.1**	**0.970**	**0.955**	0.045	**0.970**	0.030	--	--
Zwiebelbirne	14-003-1261	Koppigen	538	**G1**	**0.876**	**G1.2**	**0.962**	**0.876**	0.124	0.038	**0.962**	--	--
Safretbirne	14-003-1284	Koppigen	539	**G2**	**0.958**	**G2.1**	**0.958**	0.042	**0.958**	--	--	0.661	0.339
Hängler	14-003-1060	Koppigen	540	**G2**	**0.943**	**G2.1**	**0.943**	0.057	**0.943**	--	--	0.722	0.278
Grossmutterbirne	14-003-1259	Koppigen	541	**G2**	**0.872**	**G2.1**	**0.872**	0.128	**0.872**	--	--	0.641	0.359
Ankenbirne	14-003-1306	Koppigen	542	**G1**	**0.934**	**G1.2**	**0.934**	**0.934**	0.066	0.476	0.524	--	--
Stielbirne	14-003-1326	Koppigen	543	**G2**	**0.968**	**G2.2**	**0.968**	0.032	**0.968**	--	--	0.381	0.619
Jakob	14-003-1291	Koppigen	544	**G1**	**0.971**	**G1.1**	**0.971**	**0.971**	0.029	**0.921**	0.079	--	--
Herzbirne	14-003-1311	Koppigen	545	**G1**	0.755	**G1.2**	**0.943**	0.755	0.245	0.057	**0.943**	--	--
Siegelbirne	14-003-1255	Koppigen	546	**G2**	**0.977**	**G2.1**	**0.977**	0.023	**0.977**	--	--	0.513	0.487
Ankenbirne	14-003-1237	Koppigen	547	**G1**	**0.983**	**G1.1**	**0.983**	**0.983**	0.017	**0.972**	0.028	--	--
Hirschbirli	14-003-1238	Koppigen	548	**G2**	0.750	**G2.2**	0.750	0.250	0.750	--	--	0.481	0.519
unknown	14-003-1301	Koppigen	549	**G1**	**0.983**	**G1.1**	**0.987**	**0.983**	0.017	**0.987**	0.013	--	--
Martinsbirne	14-003-993	Roggwil Hofen	550	**G2**	**0.963**	**G2.1**	**0.963**	0.037	**0.963**	--	--	**0.868**	0.132
Richlisbirne	14-003-500	Roggwil Hofen	551	**G2**	**0.968**	**G2.2**	**0.968**	0.032	**0.968**	--	--	0.115	**0.885**
Blutbirne	14-003-68	Roggwil Hofen	552	**G1**	0.538	**G1.2**	**0.931**	0.538	0.462	0.069	**0.931**	--	--
Müsler	14-003-425	Roggwil Hofen	553	**G2**	**0.888**	**G2.2**	**0.888**	0.112	**0.888**	--	--	0.304	0.696
Gerzler Birne	14-003-242	Roggwil Hofen	554	**G2**	**0.958**	**G2.2**	**0.958**	0.042	**0.958**	--	--	0.112	**0.888**
Schwyzerhose	14-003-568	Roggwil Hofen	555	**G1**	**0.946**	**G1.1**	**0.946**	**0.946**	0.054	0.660	0.340	--	--
Strickbirne	14-003-3673	Roggwil Hofen	556	**G2**	**0.896**	**G2.2**	**0.896**	0.104	**0.896**	--	--	0.205	0.795
Trübler	14-003-3674	Roggwil Hofen	557	**G2**	**0.955**	**G2.2**	**0.955**	0.045	**0.955**	--	--	0.075	**0.925**
Kollersbirne	14-003-340	Roggwil Hofen	558	**G2**	**0.968**	**G2.1**	**0.968**	0.032	**0.968**	--	--	**0.895**	0.105
Schoggolabirne	14-003-559	Roggwil Hofen	559	**G1**	0.620	**G1.2**	**0.921**	0.620	0.380	0.079	**0.921**	--	--
Herbstgütler	14-003-3670	Roggwil Hofen	560	**G1**	0.518	**G1.1**	0.689	0.518	0.482	0.689	0.311	--	--
Sommereierbirne	14-003-581	Roggwil Hofen	561	**G2**	**0.836**	**G2.1**	**0.836**	0.164	**0.836**	--	--	0.587	0.413
Lugnezer Honigbirne	14-003-375	Roggwil Hofen	562	**G2**	**0.957**	**G2.1**	**0.957**	0.043	**0.957**	--	--	0.583	0.417
Rotlängler Dörrbirne	14-003-526	Roggwil Hofen	563	**G2**	**0.923**	**G2.2**	**0.923**	0.077	**0.923**	--	--	0.467	0.533
Hanslibirne	14-003-288	Aubonne	564	**G1**	**0.963**	**G1.2**	**0.963**	**0.963**	0.037	0.468	0.532	--	--
Petit Gollia	14-003-458	Aubonne	565	**G2**	**0.976**	**G2.1**	**0.976**	0.024	**0.976**	--	--	**0.917**	0.083
De Versvey	14-003-2527	Aubonne	566	**G1**	0.563	**G1.2**	**0.963**	0.563	0.437	0.037	**0.963**	--	--
Oberuzwiler	-	privat	567	**G1**	**0.936**	**G1.1**	**0.963**	**0.936**	0.064	**0.963**	0.037	--	--
Collonges	14-003-2505	Aubonne	568	**G1**	0.567	**G1.2**	0.748	0.567	0.433	0.252	0.748	--	--
Forel	14-003-2510	Aubonne	569	**G2**	**0.862**	**G2.1**	**0.862**	0.138	**0.862**	--	--	0.578	0.422
Wespenbirne	14-003-667	Aubonne	570	**G1**	0.621	**G1.2**	**0.974**	0.621	0.379	0.026	**0.974**	--	--
Corsinges	14-003-2506	Aubonne	571	**G1**	**0.960**	**G1.2**	**0.960**	**0.960**	0.040	0.131	**0.869**	--	--
Fossati	14-003-2511	Aubonne	572	**G1**	0.638	**G1.2**	**0.897**	0.638	0.362	0.103	**0.897**	--	--
Allinges	14-003-9	Aubonne	573	**G1**	**0.883**	**G1.1**	**0.883**	**0.883**	0.117	0.735	0.265	--	--
Bataille des Evouettes	14-003-34	Aclens	574	**G1**	0.724	**G1.2**	**0.979**	0.724	0.276	0.021	**0.979**	--	--
Blesson à longue queue	14-003-63	Aclens	575	**G2**	0.517	**G2.2**	0.732	0.483	0.517	--	--	0.268	0.732
Poire Corsinge	14-003-2534	Aclens	576	**G1**	**0.944**	**G1.2**	**0.944**	**0.944**	0.056	0.081	**0.919**	--	--
La Fribourgeoise	14-003-351	Aclens	577	**G2**	**0.940**	**G2.1**	**0.940**	0.060	**0.940**	--	--	**0.857**	0.143
Poire Pétolin	14-003-459	Aclens	578	**G2**	0.519	**G2.2**	0.782	0.481	0.519	--	--	0.218	0.782
Poire Pueta Pé	14-003-2531	Aclens	579	**G1**	**0.853**	**G1.2**	**0.853**	**0.853**	0.147	0.344	0.656	--	--
Roussette	14-003-2526	Aclens	580	**G1**	0.758	**G1.2**	**0.966**	0.758	0.242	0.034	**0.966**	--	--
Poire Sanguinole	14-003-547	Aclens	581	**G1**	0.730	**G1.2**	**0.970**	0.730	0.270	0.030	**0.970**	--	--
Poire Bacon	14-003-23	Aclens	582	**G1**	**0.957**	**G1.1**	**0.957**	**0.957**	0.043	0.692	0.308	--	--
Poire Caloué	14-003-96	Aclens	583	**G1**	0.717	**G1.2**	0.754	0.717	0.283	0.246	0.754	--	--

Poire Calouet	14-003-97	Aclens	584	**G1**	0.633	**G1.2**	0.679	0.633	0.367	0.321	0.679	--	--
Poire Caoué	14-003-102	Aclens	585	**G2**	0.798	**G2.2**	0.798	0.202	0.798	--	--	0.445	0.555
Poire de Fer	14-003-1146	Aclens	586	**G2**	**0.865**	**G2.2**	**0.865**	0.135	**0.865**	--	--	0.179	**0.821**
Poire du Four	14-003-1148	Aclens	587	**G2**	**0.919**	**G2.1**	**0.919**	0.081	**0.919**	--	--	**0.804**	0.196
Poire Golliatson	14-003-2544	Aclens	588	**G2**	**0.812**	**G2.2**	**0.812**	0.188	**0.812**	--	--	0.434	0.566
Poire Mocaté	14-003-1151	Aclens	589	**G2**	0.601	**G2.1**	0.683	0.399	0.601	--	--	0.683	0.317
Poire Olivette	14-003-1152	Aclens	590	**G2**	0.701	**G2.2**	0.701	0.299	0.701	--	--	0.499	0.501
Poire Piasse	14-003-1153	Aclens	591	**G1**	0.797	**G1.2**	**0.933**	0.797	0.203	0.067	**0.933**	--	--
Poire rouge	14-003-1560	Aclens	592	**G2**	**0.881**	**G2.1**	**0.881**	0.119	**0.881**	--	--	0.590	0.410
Rousselette	14-003-1545	Aclens	593	**G1**	**0.965**	**G1.2**	**0.974**	**0.965**	0.035	0.026	**0.974**	--	--
Saint-Jean	14-003-1546	Aclens	594	**G1**	**0.967**	**G1.2**	**0.967**	**0.967**	0.033	0.085	**0.915**	--	--
Poire de Soveillame	14-003-1562	Aclens	595	**G1**	**0.968**	**G1.1**	**0.968**	**0.968**	0.032	**0.961**	0.039	--	--
Poire Vigneron	14-003-2539	Aclens	596	**G2**	0.589	**G2.2**	0.589	0.411	0.589	--	--	0.463	0.537
Duret	14-003-177	Pierre-à-bot	597	**G2**	0.751	**G2.2**	0.792	0.249	0.751	--	--	0.208	0.792
Poire Amboney	14-003-11	Pierre-à-bot	598	**G2**	0.612	**G2.2**	0.642	0.388	0.612	--	--	0.358	0.642
Poire Bellosin	14-003-42	Pierre-à-bot	599	**G2**	0.528	**G2.2**	0.573	0.472	0.528	--	--	0.427	0.573
Beurré superfin	14-003-1568	Pierre-à-bot	600	**G1**	0.789	**G1.1**	**0.933**	0.789	0.211	**0.933**	0.067	--	--
Poire Biasson	14-003-56	Pierre-à-bot	601	**G1**	**0.838**	**G1.2**	**0.889**	**0.838**	0.162	0.111	**0.889**	--	--
Poire Blanc	14-003-61	Pierre-à-bot	602	**G1**	**0.862**	**G1.2**	**0.951**	**0.862**	0.138	0.049	**0.951**	--	--
Poire Blesson Blanc du Versé	14-003-64	Pierre-à-bot	603	**G2**	0.583	**G2.2**	0.799	0.417	0.583	--	--	0.201	0.799
Poire Brunet	14-003-87	Pierre-à-bot	604	**G2**	**0.927**	**G2.1**	**0.932**	0.073	**0.927**	--	--	**0.932**	0.068
Poire Campanard	14-003-98	Pierre-à-bot	605	**G1**	0.665	**G1.2**	**0.921**	0.665	0.335	0.079	**0.921**	--	--
Poire Canevet	14-003-100	Pierre-à-bot	606	**G1**	0.596	**G1.2**	**0.908**	0.596	0.404	0.092	**0.908**	--	--
Poire Carmagnoule	14-003-104	Pierre-à-bot	607	**G1**	0.702	**G1.2**	**0.857**	0.702	0.298	0.143	**0.857**	--	--
Poire Cent-Grappes blanc	14-003-107	Pierre-à-bot	608	**G2**	0.697	**G2.2**	0.697	0.303	0.697	--	--	0.453	0.547
Charles Cognier	14-003-1556	Pierre-à-bot	609	**G1**	**0.960**	**G1.1**	**0.960**	**0.960**	0.040	0.587	0.413	--	--
Poire Citron, Poire-	14-003-124	Pierre-à-bot	610	**G1**	**0.981**	**G1.1**	**0.981**	**0.981**	0.019	**0.874**	0.126	--	--
Poire Clochette	14-003-128	Pierre-à-bot	611	**G2**	**0.974**	**G2.1**	**0.974**	0.026	**0.974**	--	--	**0.921**	0.079
Poire Coquinet	14-003-1121	Pierre-à-bot	612	**G2**	0.647	**G2.1**	0.647	0.353	0.647	--	--	0.612	0.388
Poire Coton	14-003-142	Pierre-à-bot	613	**G1**	0.628	**G1.2**	0.702	0.628	0.372	0.298	0.702	--	--
Poire Cuvette	14-003-156	Pierre-à-bot	614	**G1**	0.546	**G1.2**	**0.830**	0.546	0.454	0.170	**0.830**	--	--
Poire Damette	14-003-157	Pierre-à-bot	615	**G2**	0.526	**G2.2**	**0.824**	0.474	0.526	--	--	0.176	**0.824**
Poire Déba	14-003-158	Pierre-à-bot	616	**G2**	**0.859**	**G2.1**	**0.859**	0.141	**0.859**	--	--	0.573	0.427
Poire Dzojè	14-003-178	Pierre-à-bot	617	**G2**	0.740	**G2.1**	0.768	0.260	0.740	--	--	0.768	0.232
Poire Epi d'Hiver	14-003-186	Pierre-à-bot	618	**G1**	**0.828**	**G1.1**	**0.828**	**0.828**	0.172	0.516	0.484	--	--
Poire Epine	14-003-1123	Pierre-à-bot	619	**G1**	0.638	**G1.2**	**0.981**	0.638	0.362	0.019	**0.981**	--	--
Poire de Fer	14-003-1124	Pierre-à-bot	620	**G1**	0.789	**G1.1**	0.789	0.789	0.211	0.697	0.303	--	--
Poire Froment	14-003-1125	Pierre-à-bot	621	**G2**	0.575	**G2.2**	0.775	0.425	0.575	--	--	0.225	0.775
Poire Froment	14-003-1126	Pierre-à-bot	622	**G2**	0.767	**G2.2**	0.767	0.233	0.767	--	--	0.480	0.520
Poire Goyard	14-003-1128	Pierre-à-bot	623	**G2**	0.641	**G2.2**	0.733	0.359	0.641	--	--	0.267	0.733
Poire Goyatson	14-003-258	Pierre-à-bot	624	**G2**	**0.818**	**G2.1**	**0.818**	0.182	**0.818**	--	--	0.556	0.444
Poire Grachet	14-003-259	Pierre-à-bot	625	**G2**	**0.932**	**G2.2**	**0.932**	0.068	**0.932**	--	--	0.474	0.526
Poire Guertelet	14-003-276	Pierre-à-bot	626	**G2**	0.740	**G2.2**	**0.821**	0.260	0.740	--	--	0.179	**0.821**
Poire Guivertet	14-003-279	Pierre-à-bot	627	**G2**	**0.928**	**G2.2**	**0.928**	0.072	**0.928**	--	--	0.238	0.762
Poire Madeleine	14-003-383	Pierre-à-bot	628	**G1**	**0.868**	**G1.2**	**0.940**	**0.868**	0.132	0.060	**0.940**	--	--
Poire Malsalé	14-003-385	Pierre-à-bot	629	**G2**	**0.831**	**G2.1**	**0.831**	0.169	**0.831**	--	--	0.591	0.409
Martinsec panaché	14-003-1569	Pierre-à-bot	630	**G1**	0.653	**G1.2**	**0.882**	0.653	0.347	0.118	**0.882**	--	--
Poire à Miel	14-003-404	Pierre-à-bot	631	**G1**	**0.937**	**G1.2**	**0.979**	**0.937**	0.063	0.021	**0.979**	--	--
Poire Mocaté	14-003-407	Pierre-à-bot	632	**G1**	0.592	**G1.2**	**0.925**	0.592	0.408	0.075	**0.925**	--	--
Poire Mocatelet	14-003-408	Pierre-à-bot	633	**G2**	0.739	**G2.1**	0.739	0.261	0.739	--	--	0.566	0.434
Orange	14-003-2543	Pierre-à-bot	634	**G1**	**0.948**	**G1.2**	**0.948**	**0.948**	0.052	0.184	**0.816**	--	--
Poire Orange	14-003-439	Pierre-à-bot	635	**G1**	0.789	**G1.2**	**0.949**	0.789	0.211	0.051	**0.949**	--	--
Poire Paremin	14-003-452	Pierre-à-bot	636	**G1**	0.712	**G1.2**	**0.935**	0.712	0.288	0.065	**0.935**	--	--
Poire Pétolin	14-003-2546	Pierre-à-bot	637	**G2**	0.512	**G2.2**	**0.834**	0.488	0.512	--	--	0.166	**0.834**
Poire Petou	14-003-460	Pierre-à-bot	638	**G2**	0.642	**G2.1**	**0.820**	0.358	0.642	--	--	**0.820**	0.180
Poire Piasse	14-003-462	Pierre-à-bot	639	**G1**	**0.923**	**G1.2**	**0.954**	**0.923**	0.077	0.046	**0.954**	--	--
Poire Piâtè	14-003-1162	Pierre-à-bot	640	**G1**	**0.867**	**G1.2**	**0.965**	**0.867**	0.133	0.035	**0.965**	--	--
Poire Piéta	14-003-466	Pierre-à-bot	641	**G1**	0.510	**G1.2**	**0.821**	0.510	0.490	0.179	**0.821**	--	--
Poire Poiratte	14-003-470	Pierre-à-bot	642	**G1**	0.733	**G1.2**	**0.959**	0.733	0.267	0.041	**0.959**	--	--
Poire à deux yeux	14-003-1543	Pierre-à-bot	643	**G1**	**0.912**	**G1.2**	**0.974**	**0.912**	0.088	0.026	**0.974**	--	--
Poire Recan	14-003-491	Pierre-à-bot	644	**G1**	0.731	**G1.2**	**0.851**	0.731	0.269	0.149	**0.851**	--	--
Poire Rindaï	14-003-503	Pierre-à-bot	645	**G1**	0.722	**G1.2**	**0.846**	0.722	0.278	0.154	**0.846**	--	--
Poire Rochet	14-003-508	Pierre-à-bot	646	**G1**	**0.844**	**G1.2**	**0.926**	**0.844**	0.156	0.074	**0.926**	--	--
Poire Ronda	14-003-512	Pierre-à-bot	647	**G1**	**0.872**	**G1.2**	**0.918**	**0.872**	0.128	0.082	**0.918**	--	--
Poire Rondi	14-003-515	Pierre-à-bot	648	**G2**	**0.808**	**G2.1**	**0.838**	0.192	**0.808**	--	--	**0.838**	0.162
Poire Rosalet	14-003-516	Pierre-à-bot	649	**G2**	0.793	**G2.1**	0.793	0.207	0.793	--	--	0.627	0.373
Poire Rosselet	14-003-519	Pierre-à-bot	650	**G1**	**0.976**	**G1.2**	**0.978**	**0.976**	0.024	0.022	**0.978**	--	--
Poire Rouge	14-003-528	Pierre-à-bot	651	**G2**	0.643	**G2.2**	0.745	0.357	0.643	--	--	0.255	0.745
Poire Roz	14-003-533	Pierre-à-bot	652	**G2**	0.764	**G2.1**	0.764	0.236	0.764	--	--	0.682	0.318
Poire de la Saint-Jean	14-003-542	Pierre-à-bot	653	**G1**	0.764	**G1.2**	**0.956**	0.764	0.236	0.044	**0.956**	--	--
Poire St. Laurent	14-003-1542	Pierre-à-bot	654	**G2**	0.577	**G2.1**	0.577	0.423	0.577	--	--	0.501	0.499
Poire Sâe	14-003-541	Pierre-à-bot	655	**G2**	0.758	**G2.2**	0.758	0.242	0.758	--	--	0.417	0.583
Poire Serpent	14-003-572	Pierre-à-bot	656	**G1**	0.568	**G1.2**	**0.983**	0.568	0.432	0.017	**0.983**	--	--
Poire Sucelet	14-003-606	Pierre-à-bot	657	**G2**	0.652	**G2.2**	**0.848**	0.348	0.652	--	--	0.152	**0.848**
Poire de Suisse	14-003-609	Pierre-à-bot	658	**G2**	0.782	**G2.1**	0.782	0.218	0.782	--	--	0.601	0.399
Poire Tanti	14-003-617	Pierre-à-bot	659	**G1**	**0.926**	**G1.2**	**0.978**	**0.926**	0.074	0.022	**0.978**	--	--
Poire de Tcheneveuil	14-003-618	Pierre-à-bot	660	**G2**	**0.914**	**G2.2**	**0.914**	0.086	**0.914**	--	--	0.350	0.650
Poire Vert-Sucré	14-003-644	Pierre-à-bot	661	**G1**	0.713	**G1.2**	0.713	0.713	0.287	0.383	0.617	--	--
Poire de Vigne	14-003-646	Pierre-à-bot	662	**G1**	0.575	**G1.2**	**0.947**	0.575	0.425	0.053	**0.947**	--	--
Poire Voidja	14-003-649	Pierre-à-bot	663	**G1**	0.709	**G1.2**	0.709	0.709	0.291	0.446	0.554	--	--
Poire Allemand	14-003-1188	Pierre-à-bot	664	**G2**	0.572	**G2.1**	0.751	0.428	0.572	--	--	0.751	0.249
Poire Chevaux	?	Pierre-à-bot	665	**G1**	0.587	**G1.2**	**0.968**	0.587	0.413	0.032	**0.968**	--	--
Culotte de Berger	?	Pierre-à-bot	666	**G1**	**0.957**	**G1.1**	**0.961**	**0.957**	0.043	**0.961**	0.039	--	--
Poire Fricot	?	Pierre-à-bot	667	**G2**	**0.902**	**G2.2**	**0.902**	0.098	**0.902**	--	--	0.173	**0.827**
Rosellette	14-003-1019	Baden	668	**G1**	**0.966**	**G1.1**	**0.966**	**0.966**	0.034	**0.882**	0.118	--	--
Champagner	14-003-1035	Baden	669	**G2**	0.504	**G2.2**	0.566	0.496	0.504	--	--	0.434	0.566
Strubikner	14-003-1041	Baden	670	**G1**	**0.922**	**G1.2**	**0.922**	**0.922**	0.078	0.222	0.778	--	--

unknown	14-003-1050	Baden	671	**G1**	**0.977**	**G1.2**	**0.977**	**0.977**	0.023	0.319	0.681	--	--
unknown	14-003-1052	Baden	672	**G1**	**0.889**	**G1.1**	**0.889**	**0.889**	0.111	**0.817**	0.183	--	--
Ackerbirne	14-003-1054	Baden	673	**G2**	**0.958**	**G2.2**	**0.958**	0.042	**0.958**	--	--	0.480	0.520
unknown	14-003-1056	Baden	674	**G1**	**0.821**	**G1.2**	**0.943**	**0.821**	0.179	0.057	**0.943**	--	--
Feigenbirne	14-003-1058	Baden	675	**G2**	**0.822**	**G2.2**	**0.822**	0.178	**0.822**	--	--	0.227	0.773
Grumbirne	14-003-1062	Baden	676	**G2**	0.531	**G2.2**	0.596	0.469	0.531	--	--	0.404	0.596
Zimmermann	14-003-1067	Baden	677	**G1**	**0.956**	**G1.2**	**0.956**	**0.956**	0.044	0.103	**0.897**	--	--
Zitronenbirne	14-003-1071	Baden	678	**G2**	0.533	**G2.1**	**0.830**	0.467	0.533	--	--	**0.830**	0.170
Stümpler	14-003-1076	Baden	679	**G2**	**0.826**	**G2.2**	**0.826**	0.174	**0.826**	--	--	0.448	0.552
unknown	14-003-1077	Baden	680	**G1**	0.724	**G1.2**	**0.926**	0.724	0.276	0.074	**0.926**	--	--
unknown	14-003-1080	Baden	681	**G1**	**0.883**	**G1.2**	**0.968**	**0.883**	0.117	0.032	**0.968**	--	--
Chilbi Birne	14-003-1082	Baden	682	**G2**	**0.851**	**G2.1**	**0.851**	0.149	**0.851**	--	--	0.755	0.245
unknown	14-003-1084	Baden	683	**G1**	**0.871**	**G1.2**	**0.871**	**0.871**	0.129	0.331	0.669	--	--
unknown	14-003-1085	Baden	684	**G1**	**0.959**	**G1.1**	**0.959**	**0.959**	0.041	**0.941**	0.059	--	--
unknown	14-003-1088	Baden	685	**G2**	0.590	**G2.2**	**0.910**	0.410	0.590	--	--	0.090	**0.910**
Santa Maria	14-003-1042	Baden	686	**G1**	0.622	**G1.1**	0.622	0.622	0.378	0.618	0.382	--	--
A cuire	14-003-1555	Baden	687	**G2**	**0.803**	**G2.2**	**0.803**	0.197	**0.803**	--	--	0.478	0.522
unknown / Louise bonne de la Tuilière	14-003-1557	Baden	688	**G1**	**0.975**	**G1.1**	**0.975**	**0.975**	0.025	**0.944**	0.056	--	--
Marion	14-003-1574	Baden	689	**G2**	0.639	**G2.1**	**0.832**	0.361	0.639	--	--	**0.832**	0.168
Rädler	14-003-486	Baden	690	**G2**	**0.981**	**G2.2**	**0.981**	0.019	**0.981**	--	--	0.087	**0.913**
Züccoh	14-003-690	Baden	691	**G1**	**0.944**	**G1.1**	**0.964**	**0.944**	0.056	**0.964**	0.036	--	--
Schneebirne	14-003-1317	Baden	692	**G2**	0.664	**G2.1**	0.664	0.336	0.664	--	--	0.550	0.450
unknown	14-003-1094	Baden	693	**G1**	**0.951**	**G1.1**	**0.951**	**0.951**	0.049	0.757	0.243	--	--
Gelbe Butterbirne	14-003-1095	Baden	694	**G1**	0.743	**G1.2**	0.753	0.743	0.257	0.247	0.753	--	--
unknown	14-003-1347	Baden	695	**G2**	**0.939**	**G2.2**	**0.939**	0.061	**0.939**	--	--	0.220	0.780
Keinerlei	14-003-1365	Baden	696	**G2**	**0.934**	**G2.2**	**0.934**	0.066	**0.934**	--	--	0.117	**0.883**
Wettinger	14-003-1377	Baden	697	**G2**	**0.865**	**G2.2**	**0.931**	0.135	**0.865**	--	--	0.069	**0.931**
Hängler	14-003-295	Baden	698	**G2**	**0.913**	**G2.1**	**0.913**	0.087	**0.913**	--	--	0.589	0.411
Stammbirne	14-003-1444	Baden	699	**G2**	**0.966**	**G2.2**	**0.966**	0.034	**0.966**	--	--	0.240	0.760
unknown	14-003-1445	Baden	700	**G1**	0.740	**G1.2**	**0.815**	0.740	0.260	0.185	**0.815**	--	--
Naghin's Butterbirne	14-003-428	Baden	701	**G1**	**0.975**	**G1.1**	**0.984**	**0.975**	0.025	**0.984**	0.016	--	--
Chriesibirne	14-003-123	Baden	702	**G1**	**0.806**	**G1.1**	**0.851**	**0.806**	0.194	**0.851**	0.149	--	--
Kugelbirne	14-003-347	Baden	703	**G1**	0.756	**G1.2**	**0.843**	0.756	0.244	0.157	**0.843**	--	--
Wilde Eierbirne	14-003-669	Baden	704	**G2**	**0.977**	**G2.1**	**0.977**	0.023	**0.977**	--	--	0.723	0.277
Dörrbirne	14-003-529	Baden	705	**G2**	0.715	**G2.1**	**0.810**	0.285	0.715	--	--	**0.810**	0.190
Sarganser Dörrbirne	14-003-712	Baden	706	**G2**	**0.942**	**G2.2**	**0.942**	0.058	**0.942**	--	--	0.166	**0.834**
unknown	14-003-693	Baden	707	**G2**	**0.892**	**G2.2**	**0.892**	0.108	**0.892**	--	--	0.250	0.750
Sternenbirne	14-003-700	Baden	708	**G2**	**0.949**	**G2.2**	**0.949**	0.051	**0.949**	--	--	0.429	0.571
Miltibirne	14-003-707	Baden	709	**G2**	**0.972**	**G2.1**	**0.972**	0.028	**0.972**	--	--	0.733	0.267
Söilibirne	14-003-1489	Baden	710	**G1**	0.618	**G1.2**	0.618	0.618	0.382	0.482	0.518	--	--
Grosse Weissbirne	14-003-702	Baden	711	**G2**	**0.922**	**G2.2**	**0.922**	0.078	**0.922**	--	--	0.397	0.603
Biomättler	14-003-1490	Baden	712	**G2**	**0.966**	**G2.2**	**0.966**	0.034	**0.966**	--	--	0.323	0.677
Heubirli	14-003-1399	Baden	713	**G2**	**0.904**	**G2.2**	**0.904**	0.096	**0.904**	--	--	0.341	0.659
Aenisbirne	14-003-1400	Baden	714	**G1**	0.800	**G1.2**	**0.912**	0.800	0.200	0.088	**0.912**	--	--
Thurgauer Weinbirne	14-003-742	Baden	715	**G2**	**0.945**	**G2.1**	**0.945**	0.055	**0.945**	--	--	0.643	0.357
Togge	14-003-743	Baden	716	**G1**	0.682	**G1.2**	**0.877**	0.682	0.318	0.123	**0.877**	--	--
Rotlängele	14-003-1456	Baden	717	**G1**	**0.942**	**G1.1**	**0.942**	**0.942**	0.058	**0.828**	0.172	--	--
Ottenbacher Schellerbirne	14-003-1083	Baden	718	**G1**	**0.816**	**G1.2**	**0.816**	**0.816**	0.184	0.192	**0.808**	--	--
unknown	14-003-1448	Büron	719	**G1**	**0.908**	**G1.1**	**0.918**	**0.908**	0.092	**0.918**	0.082	--	--
Thorbirne	14-003-764	Büron	720	**G2**	**0.972**	**G2.2**	**0.972**	0.028	**0.972**	--	--	0.498	0.502
Sommerblutbirne	14-003-728	Büron	721	**G2**	0.756	**G2.1**	**0.815**	0.244	0.756	--	--	**0.815**	0.185
Rheinlangäla	14-003-759	Büron	722	**G1**	0.734	**G1.2**	0.734	0.734	0.266	0.319	0.681	--	--
unknown	14-003-936	Büron	723	**G1**	**0.851**	**G1.2**	**0.902**	**0.851**	0.149	0.098	**0.902**	--	--
Honigbirne	14-003-829	Büron	724	**G2**	**0.971**	**G2.2**	**0.971**	0.029	**0.971**	--	--	0.361	0.639
unknown	14-003-1314	Büron	725	**G1**	**0.970**	**G1.1**	**0.970**	**0.970**	0.030	**0.926**	0.074	--	--
Gelbbirne	14-003-974	Büron	726	**G2**	**0.960**	**G2.2**	**0.960**	0.040	**0.960**	--	--	0.103	**0.897**
unknown	14-003-1072	Büron	727	**G2**	**0.834**	**G2.2**	**0.844**	0.166	**0.834**	--	--	0.156	**0.844**
unknown	14-003-1027	Büron	728	**G1**	**0.950**	**G1.1**	**0.950**	**0.950**	0.050	**0.936**	0.064	--	--
Glockenbirne	14-003-1026	Büron	729	**G2**	**0.811**	**G2.2**	**0.811**	0.189	**0.811**	--	--	0.361	0.639
Heuerbirli	14-003-994	Büron	730	**G1**	0.740	**G1.2**	**0.988**	0.740	0.260	0.012	**0.988**	--	--
Heilämpen	14-003-985	Büron	731	**G2**	0.650	**G2.1**	**0.842**	0.350	0.650	--	--	**0.842**	0.158
unknown	14-003-925	Büron	732	**G2**	**0.977**	**G2.2**	**0.977**	0.023	**0.977**	--	--	0.353	0.647
Waldershüsler	14-003-923	Büron	733	**G2**	**0.904**	**G2.2**	**0.904**	0.096	**0.904**	--	--	0.368	0.632
Speierling	14-003-920	Büron	734	**G2**	**0.899**	**G2.2**	**0.911**	0.101	**0.899**	--	--	0.089	**0.911**
Häfelä	14-003-990	Büron	735	**G2**	**0.943**	**G2.2**	**0.943**	0.057	**0.943**	--	--	0.295	0.705
Merzelambirnen	14-003-949	Büron	736	**G2**	**0.943**	**G2.2**	**0.943**	0.057	**0.943**	--	--	0.422	0.578
Heulämpen	14-003-305	Büron	737	**G2**	**0.966**	**G2.1**	**0.966**	0.034	**0.966**	--	--	**0.850**	0.150
Hagenwiler	14-003-883	Büron	738	**G2**	**0.971**	**G2.2**	**0.971**	0.029	**0.971**	--	--	0.201	0.799
unknown	14-003-1478	Büron	739	**G2**	**0.945**	**G2.2**	**0.945**	0.055	**0.945**	--	--	0.311	0.689
Lehmbirne	14-003-976	Büron	740	**G2**	0.699	**G2.2**	0.699	0.301	0.699	--	--	0.387	0.613
Teerbirnen	14-003-821	Büron	741	**G2**	**0.974**	**G2.1**	**0.974**	0.026	**0.974**	--	--	0.647	0.353
Zitronenbirne	14-003-964	Büron	742	**G1**	**0.832**	**G1.2**	**0.905**	**0.832**	0.168	0.095	**0.905**	--	--
unknown	14-003-1005	Büron	743	**G2**	**0.904**	**G2.2**	**0.904**	0.096	**0.904**	--	--	0.392	0.608
Steinbirli	14-003-1002	Büron	744	**G2**	**0.973**	**G2.2**	**0.973**	0.027	**0.973**	--	--	0.268	0.732
Tiroler Spitzbirne	14-003-807	Büron	745	**G1**	0.523	**G1.2**	0.781	0.523	0.477	0.219	0.781	--	--
Butterbirne	14-003-822	Büron	746	**G1**	**0.978**	**G1.1**	**0.978**	**0.978**	0.022	**0.973**	0.027	--	--
Eierbirne	14-003-795	Büron	747	**G2**	**0.970**	**G2.2**	**0.970**	0.030	**0.970**	--	--	0.125	**0.875**
Herrenbirne	14-003-799	Büron	748	**G2**	**0.958**	**G2.1**	**0.958**	0.042	**0.958**	--	--	0.733	0.267
unknown	14-003-1435	Büron	749	**G1**	**0.956**	**G1.1**	**0.956**	**0.956**	0.044	**0.890**	0.110	--	--
Brünnlerbirne	14-003-863	Büron	750	**G2**	**0.977**	**G2.2**	**0.977**	0.023	**0.977**	--	--	0.250	0.750
Schwizer Birne	14-003-1486	Büron	751	**G2**	**0.973**	**G2.2**	**0.973**	0.027	**0.973**	--	--	0.427	0.573
Rosaletenbirne	14-003-895	Büron	752	**G1**	**0.963**	**G1.2**	**0.974**	**0.963**	0.037	0.026	**0.974**	--	--
unknown	14-003-711	Büron	753	**G2**	**0.971**	**G2.1**	**0.971**	0.029	**0.971**	--	--	0.756	0.244
Rosalettli	14-003-966	Büron	754	**G1**	**0.939**	**G1.2**	**0.939**	**0.939**	0.061	0.204	0.796	--	--
Hüehnerbeere	14-003-953	Büron	755	**G2**	**0.869**	**G2.2**	**0.869**	0.131	**0.869**	--	--	0.274	0.726
unknown	14-003-1010	Büron	756	**G1**	0.747	**G1.1**	0.747	0.747	0.253	0.521	0.479	--	--
Eierbirne	14-003-996	Büron	757	**G2**	**0.868**	**G2.1**	**0.922**	0.132	**0.868**	--	--	**0.922**	0.078

unknown	14-003-1488	Büron	758	**G1**	**0.940**	**G1.2**	**0.940**	**0.940**	0.060	0.356	0.644	--	--
Chärbeli	14-003-1000	Büron	759	**G2**	**0.948**	**G2.1**	**0.948**	0.052	**0.948**	--	--	**0.820**	0.180
Wiedlibiere	14-003-957	Büron	760	**G2**	**0.832**	**G2.2**	**0.926**	0.168	**0.832**	--	--	0.074	**0.926**
Winterisler	14-003-1417	Büron	761	**G2**	**0.808**	**G2.2**	**0.808**	0.192	**0.808**	--	--	0.447	0.553
Frauenbirne	14-003-1389	Büron	762	**G2**	**0.919**	**G2.2**	**0.919**	0.081	**0.919**	--	--	0.191	**0.809**
Kugelbirne	14-003-1416	Büron	763	**G2**	**0.813**	**G2.2**	**0.813**	0.187	**0.813**	--	--	0.232	0.768
unknown	14-003-1476	Büron	764	**G1**	0.530	**G1.2**	**0.972**	0.530	0.470	0.028	**0.972**	--	--
Butterbirne	14-003-969	Büron	765	**G1**	**0.940**	**G1.1**	**0.940**	**0.940**	0.060	**0.861**	0.139	--	--
Hougräbler	14-003-1414	Büron	766	**G2**	**0.961**	**G2.1**	**0.961**	0.039	**0.961**	--	--	0.758	0.242
Schiblerbirli	14-003-945	Büron	767	**G2**	0.621	**G2.2**	**0.801**	0.379	0.621	--	--	0.199	**0.801**
Wolfertswiler Blutbirne	14-003-854	Büron	768	**G1**	0.756	**G1.2**	**0.958**	0.756	0.244	0.042	**0.958**	--	--
Schnittholzbirne	14-003-847	Büron	769	**G2**	**0.965**	**G2.2**	**0.965**	0.035	**0.965**	--	--	0.255	0.745
Gemstler	14-003-1044	Büron	770	**G2**	**0.933**	**G2.2**	**0.933**	0.067	**0.933**	--	--	0.188	**0.812**
unknown	14-003-1483	Büron	771	**G1**	**0.900**	**G1.1**	**0.967**	**0.900**	0.100	**0.967**	0.033	--	--
Bettagsbirne	14-003-911	Büron	772	**G2**	**0.974**	**G2.1**	**0.974**	0.026	**0.974**	--	--	0.555	0.445
Süsse Theilersbirne	14-003-620	Büron	773	**G2**	0.615	**G2.2**	0.615	0.385	0.615	--	--	0.432	0.568
Biessenhofer Birne	14-003-887	Büron	774	**G2**	0.731	**G2.2**	0.799	0.269	0.731	--	--	0.201	0.799
Rütibirne	14-003-867	Büron	775	**G1**	**0.974**	**G1.2**	**0.974**	**0.974**	0.026	0.404	0.596	--	--
unknown	14-003-740	Büron	776	**G1**	0.611	**G1.1**	**0.855**	0.611	0.389	**0.855**	0.145	--	--
Rötelibirne	14-003-1009	Büron	777	**G2**	**0.852**	**G2.2**	**0.852**	0.148	**0.852**	--	--	0.340	0.660
Martinsbirne	14-003-1007	Büron	778	**G2**	**0.966**	**G2.2**	**0.966**	0.034	**0.966**	--	--	0.198	**0.802**
Feigenbirne	14-003-793	Büron	779	**G2**	**0.830**	**G2.2**	**0.830**	0.170	**0.830**	--	--	0.302	0.698
Eierbirne	14-003-833	Büron	780	**G1**	0.559	**G1.1**	**0.816**	0.559	0.441	**0.816**	0.184	--	--
Butterbirne	14-003-872	Büron	781	**G1**	**0.926**	**G1.1**	**0.969**	**0.926**	0.074	**0.969**	0.031	--	--
Gelbbirne	14-003-816	Büron	782	**G1**	0.612	**G1.2**	0.754	0.612	0.388	0.246	0.754	--	--
unknown	14-003-1455	Büron	783	**G2**	**0.924**	**G2.2**	**0.924**	0.076	**0.924**	--	--	0.078	**0.922**
Butterbirne	14-003-783	Büron	784	**G1**	**0.923**	**G1.1**	**0.923**	**0.923**	0.077	0.785	0.215	--	--
Brunnenbirne	14-003-88	Büron	785	**G2**	**0.967**	**G2.1**	**0.967**	0.033	**0.967**	--	--	0.567	0.433
Rostizer	14-003-820	Büron	786	**G2**	0.604	**G2.2**	0.604	0.396	0.604	--	--	0.470	0.530
Tessiner Birli	14-003-825	Büron	787	**G1**	0.544	**G1.1**	0.720	0.544	0.456	0.720	0.280	--	--
Zitronenbirne	14-003-903	Büron	788	**G1**	0.778	**G1.2**	**0.968**	0.778	0.222	0.032	**0.968**	--	--
Grüne Sommermagdalene	14-003-902	Büron	789	**G2**	**0.922**	**G2.2**	**0.922**	0.078	**0.922**	--	--	0.428	0.572
unknown	14-003-934	Büron	790	**G1**	**0.963**	**G1.1**	**0.963**	**0.963**	0.037	**0.959**	0.041	--	--
Thurgauerbirne	14-003-849	Büron	791	**G2**	**0.955**	**G2.1**	**0.955**	0.045	**0.955**	--	--	0.568	0.432
Schutzenbirne	14-003-778	Büron	792	**G2**	**0.984**	**G2.1**	**0.984**	0.016	**0.984**	--	--	0.694	0.306
Gascholere	14-003-845	Büron	793	**G2**	**0.969**	**G2.1**	**0.969**	0.031	**0.969**	--	--	**0.801**	0.199
Ankenbirne	14-003-1378	Büron	794	**G2**	0.714	**G2.2**	0.714	0.286	0.714	--	--	0.443	0.557
unknown	14-003-1372	Büron	795	**G2**	**0.975**	**G2.2**	**0.975**	0.025	**0.975**	--	--	0.098	**0.902**
Trybelbirne	14-003-1330	Büron	796	**G2**	**0.979**	**G2.2**	**0.979**	0.021	**0.979**	--	--	0.154	**0.846**
unknown	14-003-1428	Büron	797	**G1**	0.546	**G1.1**	0.568	0.546	0.454	0.568	0.432	--	--
Petersbirne	14-003-737	Büron	798	**G2**	**0.891**	**G2.1**	**0.917**	0.109	**0.891**	--	--	**0.917**	0.083
Frühhüngälä	14-003-701	Büron	799	**G2**	**0.813**	**G2.2**	**0.827**	0.187	**0.813**	--	--	0.173	**0.827**
Bambirli	14-003-979	Büron	800	**G2**	**0.977**	**G2.2**	**0.977**	0.023	**0.977**	--	--	0.127	**0.873**
unknown	14-003-1518	Büron	801	**G1**	**0.982**	**G1.1**	**0.982**	**0.982**	0.018	**0.977**	0.023	--	--
Rollibirne	14-003-980	Büron	802	**G2**	**0.962**	**G2.1**	**0.962**	0.038	**0.962**	--	--	**0.826**	0.174
Rosalette	14-003-992	Büron	803	**G1**	**0.965**	**G1.2**	**0.981**	**0.965**	0.035	0.019	**0.981**	--	--
Heubirne	14-003-858	Büron	804	**G1**	**0.963**	**G1.1**	**0.963**	**0.963**	0.037	**0.949**	0.051	--	--
Fischbächler	14-003-1074	Büron	805	**G2**	**0.886**	**G2.2**	**0.886**	0.114	**0.886**	--	--	0.138	**0.862**
unknown	14-003-1472	Büron	806	**G2**	**0.976**	**G2.2**	**0.976**	0.024	**0.976**	--	--	0.241	0.759
unknown	14-003-1533	Büron	807	**G1**	**0.962**	**G1.1**	**0.962**	**0.962**	0.038	**0.936**	0.064	--	--
Ernbirne	14-003-2568	Büron	808	**G1**	0.611	**G1.2**	**0.960**	0.611	0.389	0.040	**0.960**	--	--
Gägubirli	14-003-1515	Büron	809	**G1**	0.530	**G1.2**	**0.834**	0.530	0.470	0.166	**0.834**	--	--
unknown	14-003-1538	Büron	810	**G2**	**0.945**	**G2.2**	**0.945**	0.055	**0.945**	--	--	0.492	0.508
Zuckerbirne	14-003-998	Büron	811	**G2**	0.756	**G2.1**	0.756	0.244	0.756	--	--	0.523	0.477
unknown	14-003-1328	Büron	812	**G2**	0.753	**G2.1**	**0.846**	0.247	0.753	--	--	**0.846**	0.154
Kalchbühler	14-003-327	Büron	813	**G2**	**0.939**	**G2.2**	**0.939**	0.061	**0.939**	--	--	0.169	**0.831**
Tschupubirli	14-003-1231	Büron	814	**G2**	0.669	**G2.2**	0.669	0.331	0.669	--	--	0.375	0.625
Hämpelibire	14-003-1215	Büron	815	**G2**	**0.878**	**G2.1**	**0.878**	0.122	**0.878**	--	--	0.702	0.298
unknown	14-003-1532	Büron	816	**G2**	0.510	**G2.1**	0.577	0.490	0.510	--	--	0.577	0.423
Spalierbirne	14-003-1046	Büron	817	**G1**	**0.894**	**G1.1**	**0.894**	**0.894**	0.106	**0.877**	0.123	--	--
unknown	14-003-1225	Büron	818	**G1**	0.541	**G1.2**	**0.883**	0.541	0.459	0.117	**0.883**	--	--
Doyenné d'hiver	14-003-1528	Büron	819	**G1**	**0.954**	**G1.1**	**0.954**	**0.954**	0.046	**0.907**	0.093	--	--
Schweizer Heubirne	14-003-566	Dürrenäsch	820	**G2**	0.738	**G2.2**	**0.906**	0.262	0.738	--	--	0.094	**0.906**
Bühlbirne	14-003-90	Dürrenäsch	821	**G2**	**0.947**	**G2.2**	**0.947**	0.053	**0.947**	--	--	0.055	**0.945**
Mockenholzbirne	14-003-409	Knonau	822	**G2**	**0.943**	**G2.1**	**0.943**	0.057	**0.943**	--	--	0.576	0.424
Rotholzbirne	14-003-525	Knonau	823	**G2**	**0.980**	**G2.2**	**0.980**	0.020	**0.980**	--	--	0.221	0.779
Rosalettibirne	14-003-517	Knonau	824	**G1**	**0.962**	**G1.1**	**0.962**	**0.962**	0.038	**0.917**	0.083	--	--
Kalberrädler	14-003-326	Knonau	825	**G2**	0.735	**G2.2**	0.735	0.265	0.735	--	--	0.378	0.622
Entenbirne	14-003-185	Knonau	826	**G2**	**0.978**	**G2.1**	**0.978**	0.022	**0.978**	--	--	**0.933**	0.067
Berner Dornbirne	14-003-48	Knonau	827	**G2**	**0.966**	**G2.2**	**0.966**	0.034	**0.966**	--	--	0.080	**0.920**
Le Lectier	14-003-362	Knonau	828	**G1**	**0.949**	**G1.1**	**0.949**	**0.949**	0.051	**0.804**	0.196	--	--
Poire Steinmüribirne	14-003-594	Schaffhausen	829	**G2**	**0.918**	**G2.1**	**0.918**	0.082	**0.918**	--	--	0.761	0.239
Poire Steinmüribirne	14-003-594	Schaffhausen	829	**G2**	**0.918**	**G2.1**	**0.918**	0.082	**0.918**	--	--	0.761	0.239
Hosui	Reference 5	Uni Reading	830	**G2**	**0.803**	**G2.2**	**0.803**	0.197	**0.803**	--	--	0.367	0.633
Sülibirne	14-003-611	Bözberg-Vierlinden	831	**G2**	**0.906**	**G2.2**	**0.906**	0.094	**0.906**	--	--	0.291	0.709
Abbe Fetel	Reference 1	Uni Reading	832	**G1**	**0.977**	**G1.1**	**0.977**	**0.977**	0.023	**0.976**	0.024	--	--
Chanticleer	Reference 2	Uni Reading	833	**G1**	**0.826**	**G1.2**	**0.843**	**0.826**	0.174	0.157	**0.843**	--	--
Passe Crassane	Reference 6	Uni Reading	834	**G1**	**0.984**	**G1.1**	**0.984**	**0.984**	0.016	**0.975**	0.025	--	--
Pendula	Reference 7	Uni Reading	835	**G2**	0.590	**G2.2**	**0.837**	0.410	0.590	--	--	0.163	**0.837**
Wasserbirne	14-003-653	Höri	837	**G2**	0.530	**G2.2**	**0.814**	0.470	0.530	--	--	0.186	**0.814**
Mockenholzbirne	14-003-409	Höri	838	**G2**	**0.898**	**G2.2**	**0.898**	0.102	**0.898**	--	--	0.104	**0.896**
Birne Unterlage M	rootstock M	Höri	839	**G2**	**0.966**	**G2.1**	**0.966**	0.034	**0.966**	--	--	**0.850**	0.150
unknown	-	privat	840	**G2**	**0.942**	**G2.1**	**0.942**	0.058	**0.942**	--	--	**0.873**	0.127
