# Supplementary material for: Integration of expert knowledge in the definition of Swiss pear core collection
Source: Sci Rep. 2019 Jun 20;9:8934. doi: 10.1038/s41598-019-44871-3 (PMC6586639; doi:10.1038/s41598-019-44871-3)
Supplement: Supplementary file 2 — Supplementary Figures and Texts [file 41598_2019_44871_MOESM2_ESM.pdf]

SUPPLEMENTARY INFORMATION

Integration of expert knowledge in the definition of Swiss pear core collection

Urrestarazu J[1]*, Kägi C[2]*, Bühlmann A[3], Gassmann J[3], Santesteban LG[1], Frey JE[3], Kellerhals M[3], and Miranda C[1]

*These authors contributed equally to this work

Corresponding author: Jorge Urrestarazu (jorge.urrestarazu@unavarra.es)
Department of Agronomy, Biotechnology and Food Science, Public University of Navarre, 31006 Pamplona, Spain. Phone number: +34 948169127

[1] Department of Agronomy, Biotechnology and Food Science, Public University of Navarre, 31006 Pamplona, Spain.

[2] Federal Office for Agriculture, 3003 Bern, Switzerland.

[3] Agroscope, 8820 Wädenswil, Switzerland.

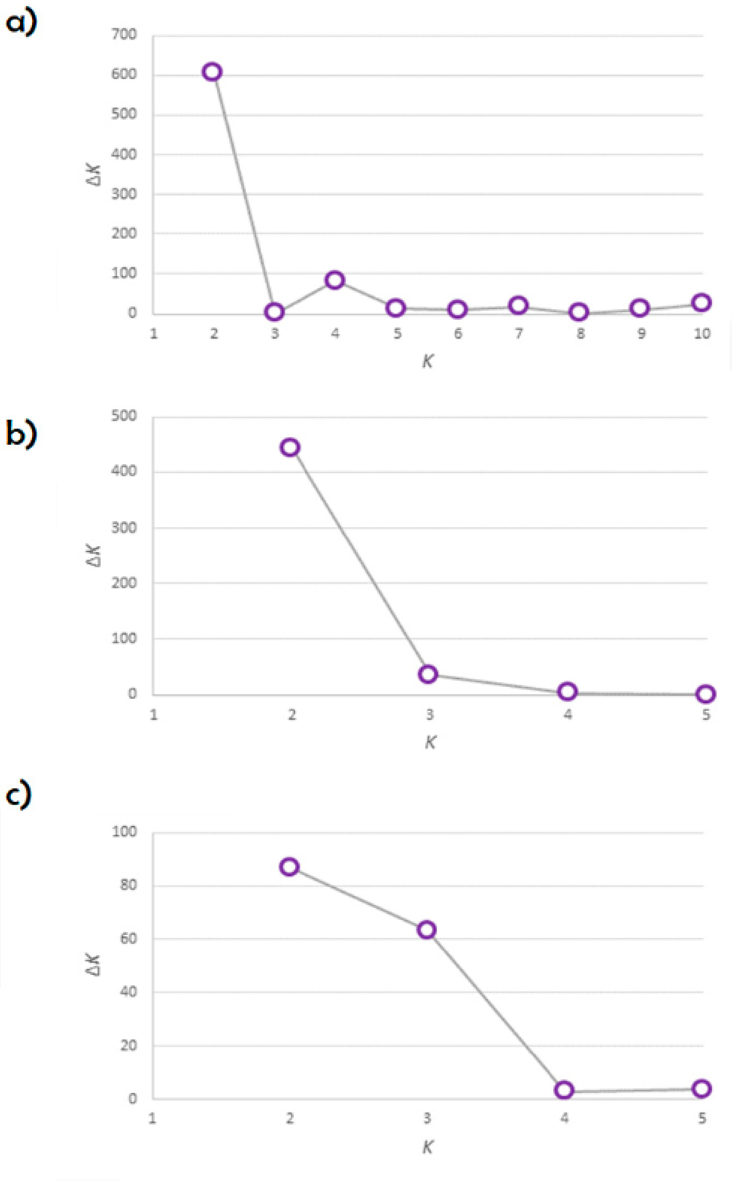

Supplementary Figure S1 Exploration of *K* value for STRUCTURE analysis of pear germplasm by estimates of the rate of change of the slope of the log likelihood curve (Δ*K*) calculated according to Evanno et al. (2005) plotted against *K*. a) Plot for the analysis on the 841 unique genotypes. b) Plot for the internal analysis of G1. c) Plot for the internal analysis of G2.

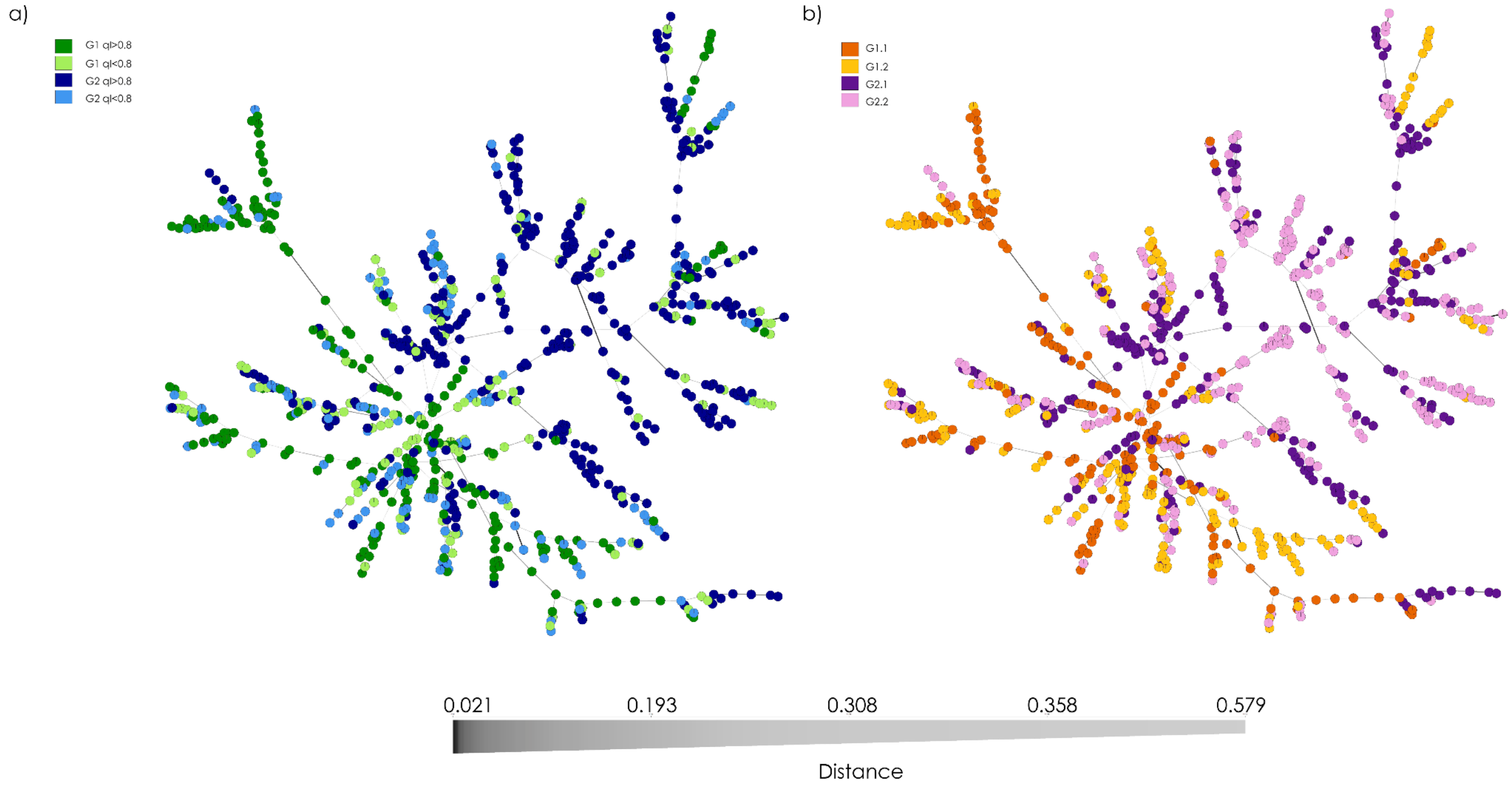

Supplementary Figure S2 Minimum spanning networks (MSN) performed on Bruvo's distances for all genotypes clustered in the groups defined by STRUCTURE at *K* = 2 (a), and for the internal analyses of G1 and G2 (b). Each node represents one genotype. Edge thickness and color are proportional to genetic distance, while edge lengths are arbitrary.

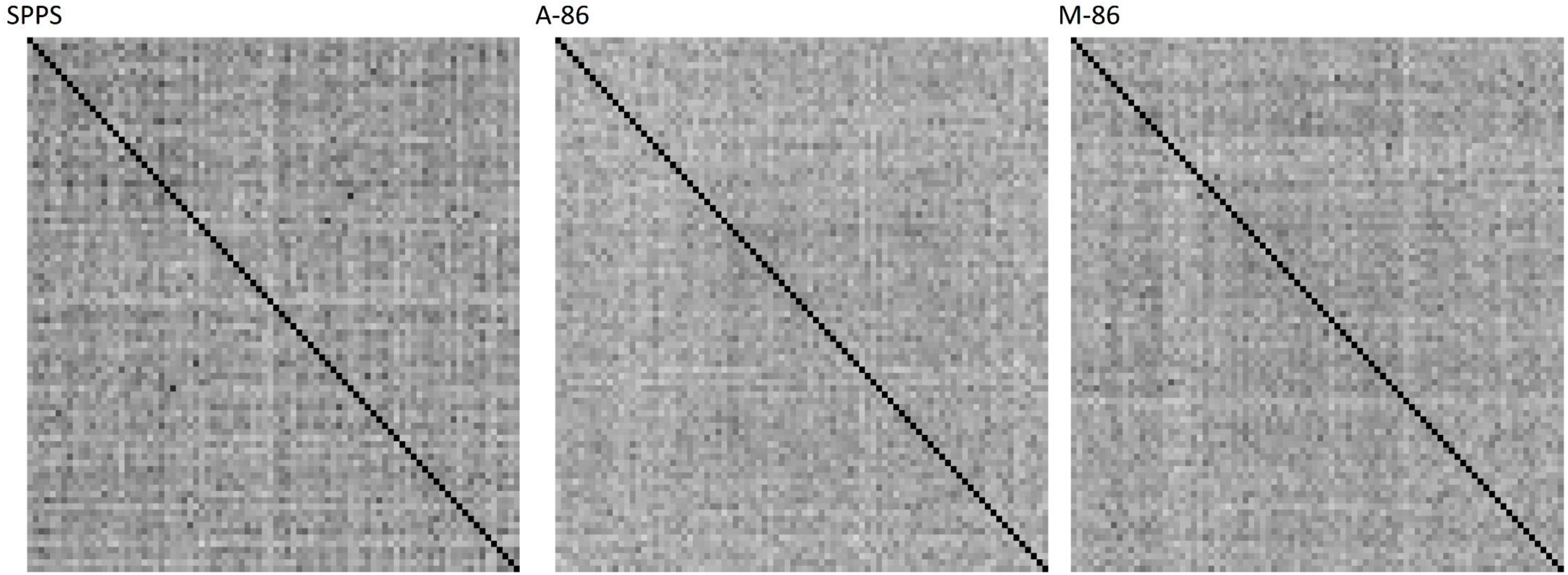

Supplementary Figure S3 Heat maps based on Bruvo’s genetic distance matrix between the genotypes included in SPPS, A-86 and M-86. Color scales indicate genetic distance between genotypes. Black indicates identical, whereas white indicates distantly related.

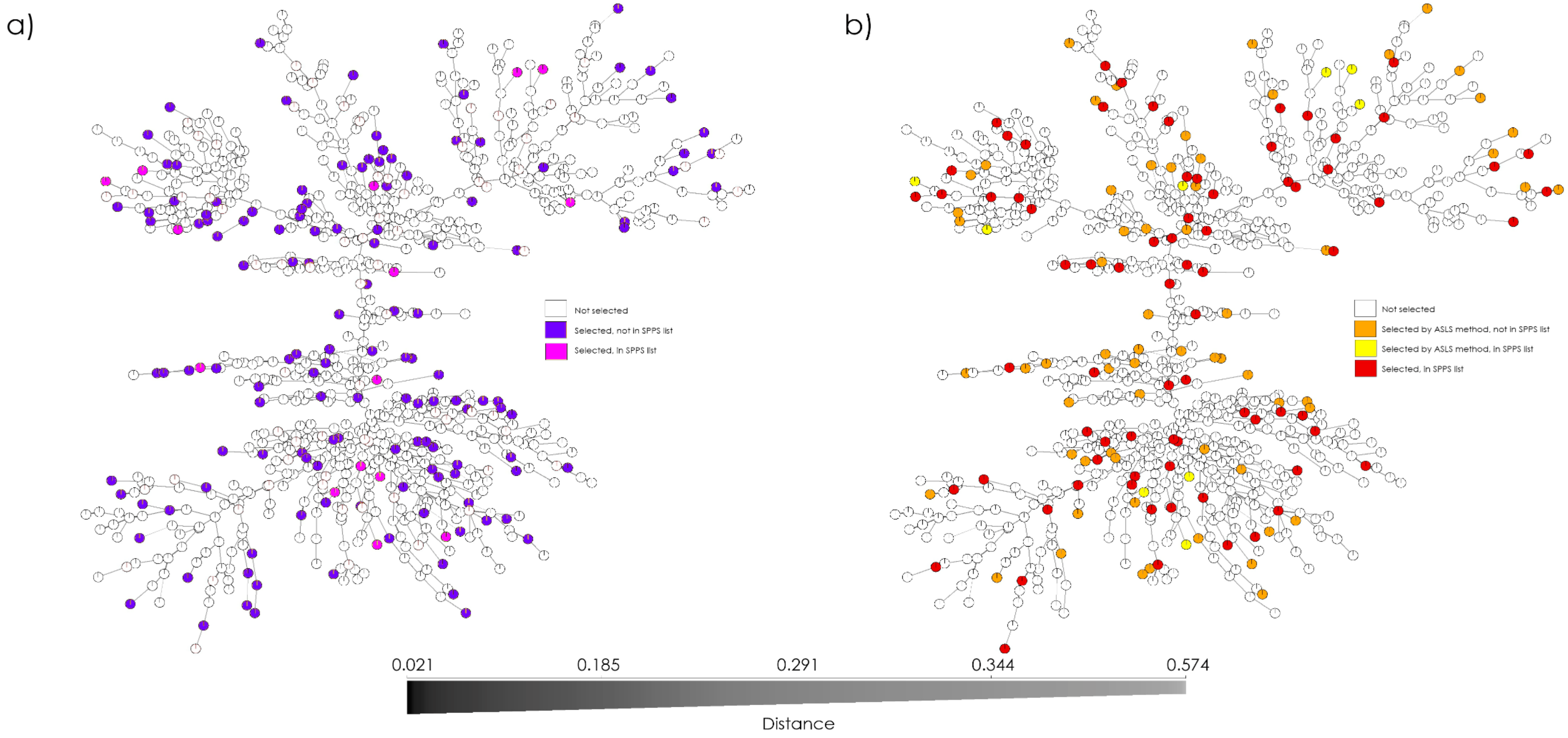

Supplementary Figure S4 Minimum spanning networks (MSN) performed on Bruvo's distances for all genotypes, highlighting genotypes sampled in A-FNK$_{All}$ (a), and in A-OMin$_{All}$ (b). Different colors in the Figure indicate if the genotype selected was included or not in the SPPS list. Each node represents one genotype. Edge thickness and color are proportional to genetic distance, while edge lengths are arbitrary.

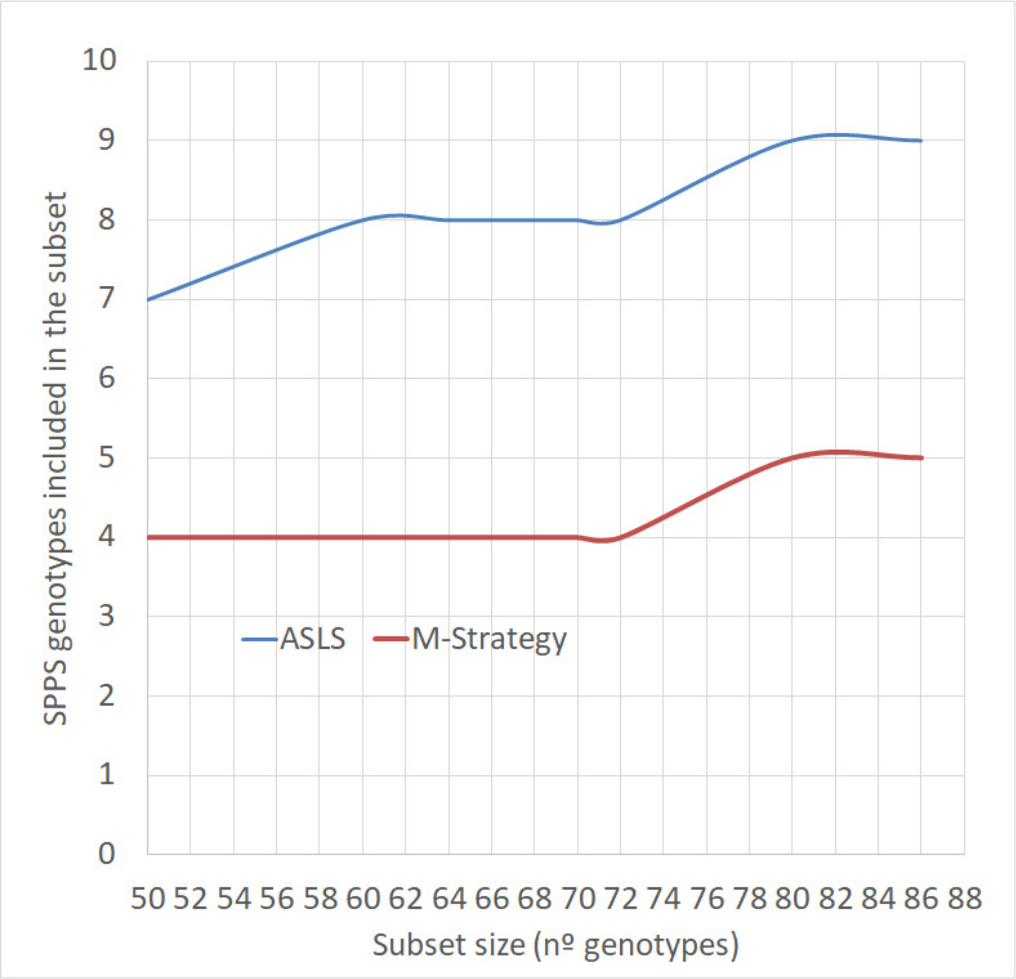

Supplementary Figure S5 Number of genotypes of the SPPS list selected by ASLS and M-Strategies depending on the target subset size.

Text S1. Genetic analyses of the Swiss National Pear Inventory

SSR polymorphism – accession identification and redundancy

Comparison of multilocus SSR profiles among the 1198 accessions allowed identifying 186 groups of accessions that had the same SSR profile (Supplementary Table S1), leading to the removal of 457 duplicated accessions before further analyses (35% of redundancy). The size of those groups varied in number from two to 21 accessions. The accessions identified as duplicates appeared within and among collections. Although some duplicates were expected, as some of the accessions had identical or very similar names, e.g., 'Fernatte'-'Poire Fernatte'-'Poire Fernattes'-'Poire Fernotte' (group 86), 'Couenla'-'Poire Couëla'-'Poire Couenla' (group 122), 'Biesson'-'Poire Bièchon'-'Poire Biesson' (group 125), or 'Muscat'-'Poire Mouchca'-'Poire Muscat'-'Poire muscat Blessens' (group 174), most groups of duplicates comprised accessions with different names. Regarding the most numerous groups of duplicates, it is worth noting that some of them comprise accessions mentioned in Pfau-Schellenberg's pomology[1], one of the most comprehensive compilation focusing on pear and apple varieties traditionally grown in Switzerland, including valuable information on putative synonymies and geographical distribution of the most relevant cultivars. One example is the 'Gelbe Mostbirne', which originates from the very eastern part of Switzerland and was widespread in the 19th century and was still found seven times in the inventory. In many cases, one pear variety had several local names as e.g. 'Längler', which is reported to be also called 'Kannenbirne', 'Wadelbirne' or 'Würgbirne' and which was found more than ten times under these different names. The present analysis would not be expected to distinguish between clones and 'sports' of cultivars with potential morphological/agronomical differences. For instance, the group 16 comprised four and three accessions named 'Poire blanc' and 'Poire gris', respectively, whereas the group 123 comprised 'Botzi jaune' and 'Botzi rouge' accessions. In these two examples, it is evident that in those groups, despite their accessions shared the same SSR profile, their names clearly indicate they differ in the color of the fruits. This large-scale study in pear constitutes a good example of the efficiency of coordinated actions based on standardized methodologies to improve the knowledge of diversity conserved at a national level. The results obtained offer a valuable step to encourage actions towards optimizing the management of pear germplasm in Switzerland.

Genetic diversity

All the SSR loci amplified in this study were polymorphic. Due to complex scoring and unreliable microsatellite profiles of locus CH05c06 under our experimental conditions, we decided to exclude it of the study. This does not imply that this marker is not suitable for fingerprinting pear accessions, in fact CH05c06 has proven to be an efficient marker in other studies carried out in pear[2,3]. A summary of the SSR allelic polymorphism revealed in the Swiss pear NPA-PGRFA collection is shown in Supplementary Table S3.

Supplementary Table S3. Genetic parameters of the 15 SSR markers used to characterize the Swiss pear NPA-PGRFA collection.

SSR locus	Size range (pb)	N_A	N_B		N_E	H_E
			$p < 0.05$	$p < 0.01$		
EMPc11	123 - 191	21	12	10	8.54	0.88
GD147	121 - 175	26	19	17	4.37	0.76
CH05f06	141 - 189	19	14	8	3.61	0.72
CH01d08	239 - 308	21	17	14	4.36	0.76
CH04c07	81 - 187	34	29	12	12.68	0.92
CH01f07a	173 - 285	28	20	16	9.28	0.89
CH01h01	101 - 127	13	11	9	2.33	0.57
CH01f03b	144 - 276	34	28	20	4.62	0.78
CH01h10	99 - 149	23	20	10	5.06	0.80
GD96	119 - 209	32	28	16	6.75	0.85
GD142	115 - 196	30	22	10	14.58	0.93
EMPc117	93 - 155	30	24	14	10.42	0.90
CHO2b10	97 - 191	35	27	18	11.01	0.91
CH03g07	166 - 276	35	28	17	8.96	0.88
CH01d09	121 - 185	31	23	13	13.31	0.92
Mean	–	27.47	21.47	13.60	7.73	0.83

N_A = number of alleles; N_B = number of alleles occurring in a frequency below 5% and 1%; N_E = number of effective alleles; H_E = expected heterozygosity

A total of 412 alleles was identified in the 841 unique genotypes across the 15 SSR markers, 78.6% and 50.0% found at frequencies below 5% and 1%, respectively. The average number of alleles per locus was 27.4 (ranging from 13 for CH01h01 to 35 for CH02b10 and CH03g07), but dropped to 5.9 and 13.7 when not considering the alleles occurring at frequencies below 5% and 1%, respectively. The mean number of effective

alleles per locus (N_E) was 7.73. Averaged over SSR loci, H_E was 0.83, and a large variation was found between SSR markers (range = 0.57-0.93; SD = 0.10).

Inference of genetic structure

Estimate of the number of hypothetical genetic groups

The 841 unique genotypes were analyzed using STRUCTURE, and the analysis of the rate of change ΔK over the range of K values showed a clear maximum for $K = 2$ ($\Delta K = 604.8$; Figure S1a). This clustering corresponds to an asymmetric partition of the germplasm in two main groups, one with 349 genotypes (G1) and a second with 492 (G2). The mean probability of assignment (qI) of the genotypes to these two groups was 0.84, and 68% of the genotypes were strongly assigned to a group, as their probability of assignment was $qI \geq 0.80$. Genetic discrimination between the two groups obtained using STRUCTURE was supported through a Minimum Spanning Network (MSN) based on the Bruvo's genetic distance between the genotypes (Figure S2a). G1 genotypes were located mostly to the left part of the plot, whereas G2 genotypes occurred mostly to the right part of the plot. The genetic differentiation among the groups defined by the Bayesian model-based clustering accounted for 3% of the variation, 5.4% when considering only the genotypes strongly assigned to the groups ($qI > 0.80$). A secondary peak at $K = 4$ (ΔK = 82.1) was observed, suggesting that the diversity could be sub-structured. Thus, a second-level (nested) application of the Structure software was applied separately on each of K groups defined in the first analysis. To assess the strength of the hypothetical subdivisions within each group (i.e. subgroups), simulations for each K value were examined, paying attention to the mean assignment probability of genotypes for the inferred subgroups, and the proportion of accessions strongly assigned ($qI \geq 0.80$)[3,4,5].

The strength of the signal for the subdivision of G1 was very much stronger than that obtained for G2. The analysis of the relationship between K and ΔK for G1 revealed a most likely subdivision at $K_{G1} = 2$ (ΔK_{G1} = 443.4) (Figure S1b). The mean assignment probability for the genotypes clustered in these two subgroups of G1 was 0.85, and 71% of the genotypes were strongly assigned. Regarding G2, two possible subdivisions were identified at $K_{G2} = 2$ ($\Delta K_{G2\text{-}2} = 100.4$) and $K_{G2} = 3$ ($\Delta K_{G2\text{-}3} = 63.2$) (Fig1c). The pattern of substructuring of G2 provided very similar results when considering $K_{G2} = 2$ and $K_{G2} = 3$, with mean assignment probabilities for the subgroups around 0.70, and proportions of strongly assigned genotypes around 25%. When different K values provide very similar

results, it is advisable to be conservative and to select the smallest level of division since captures the major substructure of the data[6]; accordingly, we adopted $\Delta K_{G2\text{-}2}$ as the most suitable level of partitioning of G2. The four subgroups (G1.1, G1.2, G2.1 and G2.2) are provided in Figure 2, as well as displayed using the same color codes in a MSN plot (Figure S2b). For these four subgroups the assignment of almost half of the genotypes (48%) to their respective subgroups was strong, the mean probability of assignment to the subgroups was 0.77, and the assignment of admixed accessions was consistent between runs. Most of the drying (82%) and cider/juice (79%) cultivars were included in G2, while the distribution of the dessert and cooking cultivars was more equilibrated between G1 and G2.

Distribution of the genotypes into the subgroups inferred

Using passport data and data of oral tradition that was collected during the inventory, as well as after reviewing old literature (i.e., national compilations, varietal catalogues, reports, etc.), we were able to classify a large part of the unique genotypes from the 841 genetic groups (ca. 70%) in four categories according to their primarily usage/aptitude (dessert, cooking, drying and cider/juice/hard liquor). The analysis of the frequency distribution of the usage/aptitude of the cultivars into the subgroups defined by STRUCTURE showed noticeable differences, and revealed interesting associations between the clustering in subgroups and the particular usage/aptitude of the cultivars (Supplementary Table S4). The two derived-subgroups from the division of G1 were mostly formed by dessert pears, especially G1.1 (72%), while the occurrence of pears of such usage/aptitude in G2.1 and G2.2 was considerably less frequent. By contrast, G2 mainly contained drying and cider/juice pears, with G2.1 having a focus on drying (42% drying pears, 26% cider/juice pears) and G2.2 on cider/juice pears (29% drying pears, 42% cider/juice pears). Chi-squared tests indicated significant differences in the distribution of the usage/aptitude of the cultivars between all the pairs of subgroups.

Supplementary Table S4. Frequency distribution of the usage/aptitude of the cultivars in then groups and subgroups defined by STRUCTURE

**Groups/subgroups**	**Usage/aptitude**			
	**Dessert**	**Cooking**	**Drying**	**Cider**
G1	59.1	15.5	12.3	13.2
G2	24.5	10.4	35.2	29.9
G1.1	71.8	12.0	9.4	6.8
G1.2	44.7	19.4	15.5	20.4
G2.1	26.4	10.7	41.6	21.3
G2.2	22.6	10.2	28.8	38.4

Intra-group variability and genetic differentiation

The four subgroups had a considerable difference in size, ranging from 161 (G1.1) to 256 (G2.2) genotypes, and variable proportion of accessions strongly assigned to the inferred subgroups (Supplementary Table S5). G1.1 and G1.2 showed 73% and 69% of strongly assigned genotypes, respectively, while the two derived-subgroups from G2 had much higher proportions of admixed genotypes (Supplementary Table S5). Accordingly, the mean assignment probability was considerably higher for the derived-subgroups of G1. With regards the genetic diversity of the subgroups, H_E varied between 0.78 (G1.1) and 0.83 (G1.2 and G2.2), and the proportion of alleles retained ranged between 65% (G1.1) and 77% (G1.2 and G2.2). Moreover, some exclusive alleles, i.e., those only found in one subgroup, were found in all subgroups, with a minimum number of 29 (G2.1) and a maximum of 94 (G1.2). Estimate of genetic differentiation showed that only 3.9% accounted for variation among subgroups, 7.4% when restricted to the genotypes strongly assigned ($qI \geq 0.80$) to the subgroups.

Supplementary Table S5. Descriptive information about the intra-group variability for pear genotypes clustered in each group/subgroup identified by the Bayesian model-based clustering method

Groups/Subgroups	Genotypes		average *qI*	H_E (range)	Alleles			
	Number	% (*qI*>0.80)			Total	*p*>0.05	Exclusive	Mean / locus
G1	349	60.17	0.82	0.82 (0.54 -0.92)	361	81	63	24.07
G2	492	73.17	0.86	0.82 (0.55-0.93)	347	87	49	23.13
G1.1	161	68.94	0.84	0.78 (0.50-0.88)	268	75	44	17.87
G1.2	188	72.87	0.86	0.83 (0.57-0.93)	318	83	94	21.20
G2.1	236	32.20	0.71	0.79 (0.51-0.92)	284	79	29	18.93
G2.2	256	39.69	0.72	0.83 (0.57-0.93)	319	85	64	21.27

1. Pfau-Schellenberg. Schweizerische Obstsorten. Schweizerischer Landwirtschaftlicher Zentralverein. St. Gallen, Wirz, 1863-1872 (1872).
2. Sehic J, Garkava-Gustavsson L, Fernández-Fernández F & Nybom, H. Genetic diversity in a collection of European pear (Pyrus communis) cultivars determined with SSR markers chosen by ECPGR. *Sci. Hortic*. **135**, 39─45 (2012).
3. Urrestarazu, J., Royo, J.B., Santesteban, L.G. & Miranda, C. Evaluating the influence of the microsatellite marker set on the genetic structure inferred in *Pyrus communis* L. *PLoS ONE* **10**, e0138417 (2015).
4. Urrestarazu, J. *et al*. Analysis of the genetic diversity and structure across a wide range of germplasm reveals prominent gene flow in apple at the European level. *BMC Plant Biol*. **16**, 130 (2016).
5. Urrestarazu, J., Errea, P., Miranda, C., Santesteban, L.G. & Pina, A. Genetic diversity of Spanish *Prunus domestica* L. germplasm reveals a complex genetic structure underlying. *PLoS ONE* **13**, e0195591 (2018).
6. Pritchard, J.K., Stephens, M. & Donnelly, P. Inference of population structure using multilocus genotype data. *Genetics* **155**, 945─59 (2000).

Text S2. Protocols for SSR genotyping.

Young leaf tissues of each accession were collected and stored at −20 °C until the DNA extractions were performed. Total genomic DNA was isolated using the Extract-N-Amp™ Plant PCR Kit (Sigma-Aldrich, St. Louis, MO, USA) according to the manufacturer's instructions. The DNA extractions were diluted 1:10 in water, and 2 μL were used as DNA template for the Polymerase chain reactions (PCR).

A set of 16 SSR primers developed by different research groups[7,8,9,10] was used to genotype the 1198 accessions of the Swiss pear NPA-PGRFA collection. The 16 SSR markers were amplified using five sets of multiplex PCR reactions (N1-N5) (Table S2). PCR reactions for the five multiplex PCRs were performed in a final volume of 11 μL, using 2 μL of the 1:10 diluted DNA extractions, 0.18 μM of each primer and 1× PCR Master mix of QIAGEN kit multiplex PCR (Qiagen, Hilden, Germany). PCR cycling conditions were as follows: initial denaturation for 15 min at 95 °C, followed by 40 cycles, each consisting of 40 s denaturing at 94 °C, 90 s at 55 °C, and 90 s elongation at 72 °C, the last cycle ending with a final 30-min extension at 60 °C. The PCR products were diluted 1:10 in H_2O, and 0.80 μL diluted PCR product was mixed with 15 μL Hi-di Formamide (Applied Biosystems, Foster City, CA, USA) and 0.25 μL 500-LIZ size standard (Applied Biosystems, Foster City, CA, USA). The fragment analyses were performed on an ABI3130 sequencing system (Applied Biosystems, Foster City, CA, USA) and PCR products analyzed and sized with GeneMapper v4.1 (Applied Biosystems, Foster City, CA, USA).

Table S2. Descriptive information of the 16 SSR markers used in this study with indication of the corresponding multiplex and dye

SSR locus	Dye	Forward primer sequence 5′→3′	Reverse primer sequence 5′→3′	Multiplex PCR	Tm (°C)
CH05c06[9]	NED	ATTGGAACTCTCCGTATTGTGC	ATCAACAGTAGTGGTAGCCGGT	N1	60.1
EMPc11[10]	VIC	GCGATTAAAGATCAATAAACCCATA	AAGCAGCTGGTTGGTGAAAT	N1	59.2
GD147[8]	PET	TCCCGCCATTTCTCTGC	GTTTAAACCGCTGCTGCTGAAC	N1	54.9
CH01d08[9]	VIC	CTCCGCCGCTATAACACTTC	TACTCTGGAGGGTATGTCAAAG	N2	60.5
CH01f07a[9]	PET	CCCTACACAGTTTCTCAACCC	CGTTTTTGGAGCGTAGGAAC	N2	61.2
CH04c07[9]	NED	GGCCTTCCATGTCTCAGAAG	CCTCATGCCCTCCACTAACA	N2	60.5
CH05f06[9]	6-FAM	TTAGATCCGGTCACTCTCCACT	TGGAGGAAGACGAAGAAGAAAG	N2	62.1
CH01f03b[9]	NED	GAGAAGCAAATGCAAAACCC	CTCCCCGGCTCCTATTCTAC	N3	56.4
CH01h01[9]	6-FAM	GAAAGACTTGCAGTGGGAGC	GGAGTGGGTTTGAGAAGGTT	N3	60.5
CH01h10[9]	PET	TGCAAAGATAGGTAGATATATGCCA	AGGAGGGATTGTTTGTGCAC	N3	60.9
EMPc117[10]	PET	GTTCTATCTACCAAGCCACGCT	CGTTTGTGTGTTTTACGTGTTG	N4	62.1
GD142[8]	NED	GGCACCCAAGCCCCTAA	GGAACCTACGACAGCAAAGTTACA	N4	57.3
GD96[8]	6-FAM	CGGCGGAAAGCAATCACCT	GCCAGCCCTCTATGGTTCCAGA	N4	59.5
CH01d09[9]	PET	GCCATCTGAACAGAATGTGC	CCCTTCATTCACATTTCCAG	N5	58.4
CH02b10[7]	VIC	CAAGGAAATCATCAAAGATTCAAG	CAAGTGGCTTCGGATAGTTG	N5	58.3
CH03g07[9]	NED	AATAAGCATTCAAAGCAATCCG	TTTTTCCAAATCGAGTTTCGTT	N5	56.4

7. Gianfranceschi, L., Seglias, N., Tarchini, R., Komjanc, M. & Gessler, C. Simple sequence repeats for the genetic analysis of apple. *Theor. Appl. Genet.* **96**, 1069–1076 (1998).
8. Hokanson SC, Szewc-McFadden AK, Lamboy WF, & McFerson, J.R. Microsatellite (SSR) markers reveal genetic identities, genetic diversity and relationships in a *Malus* x *domestica* Borkh. core subset collection. *Theor. Appl. Genet.* **97**, 671–683 (1998).
9. Liebhard, R. *et al*. Development and characterisation of 140 new microsatellites in apple (*Malus* x *domestica* Borkh.). *Mol. Breed.* **10**, 217–241 (2002).
10. Fernández-Fernández, F., Harvey, N.G. & James, C.M. Isolation and characterization of polymorphic microsatellite markers from European pear (*Pyrus communis* L.). *Mol. Ecol. Notes* **6**, 1039–1041 (2006).
